# Supplementary figures and images for: Machine learning-based identification of contrast-enhancement phase of computed tomography scans
Source: PLoS One. 2024 Feb 2;19(2):e0294581. doi: 10.1371/journal.pone.0294581 (PMC10836663; doi:10.1371/journal.pone.0294581)

A) LR

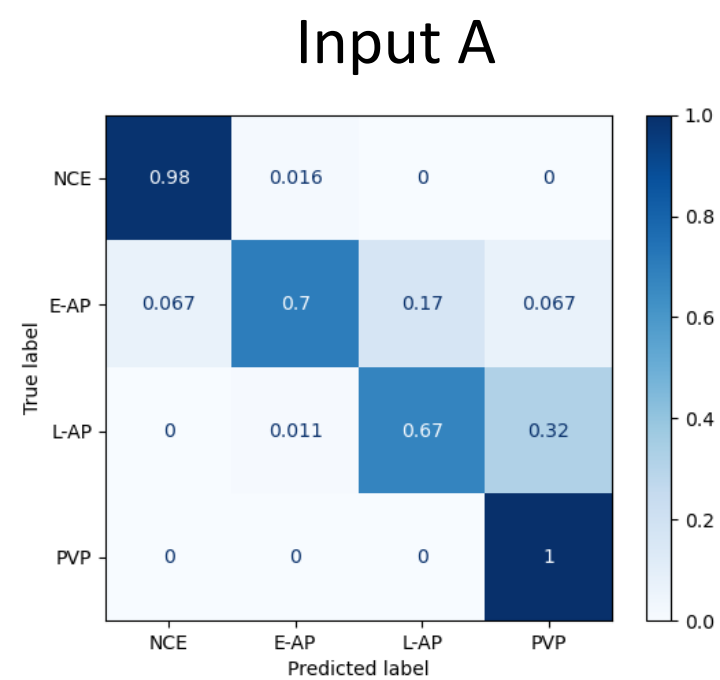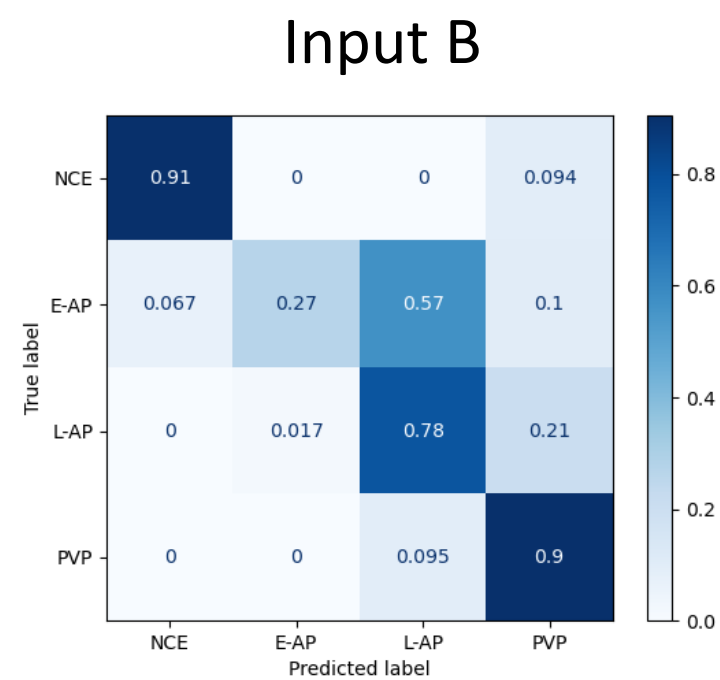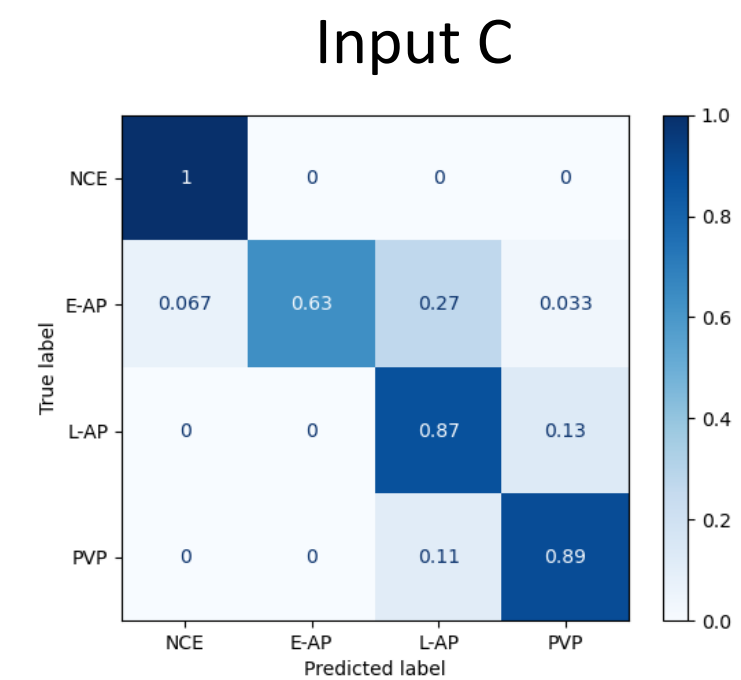

B) SVM

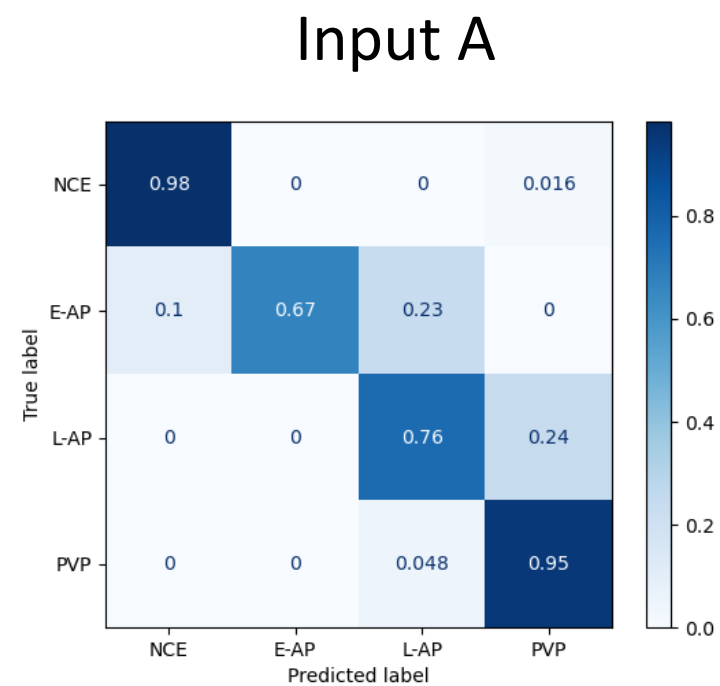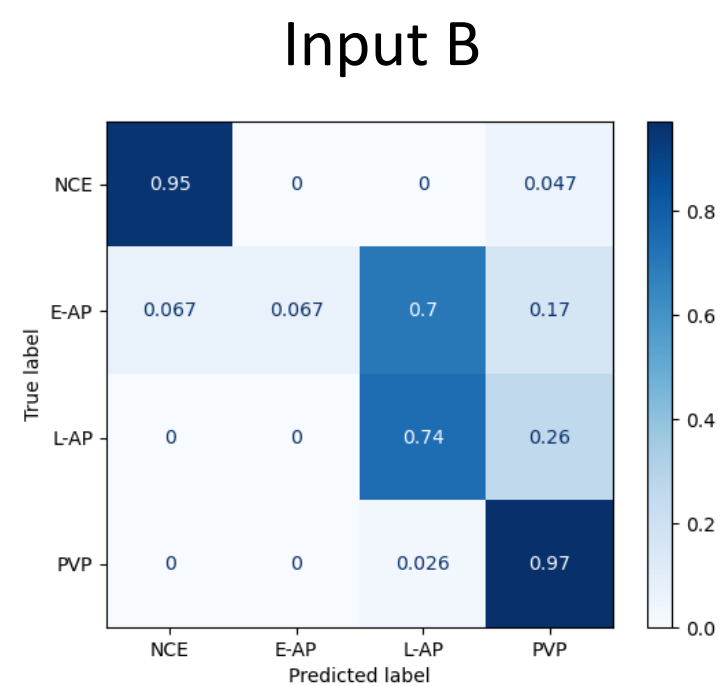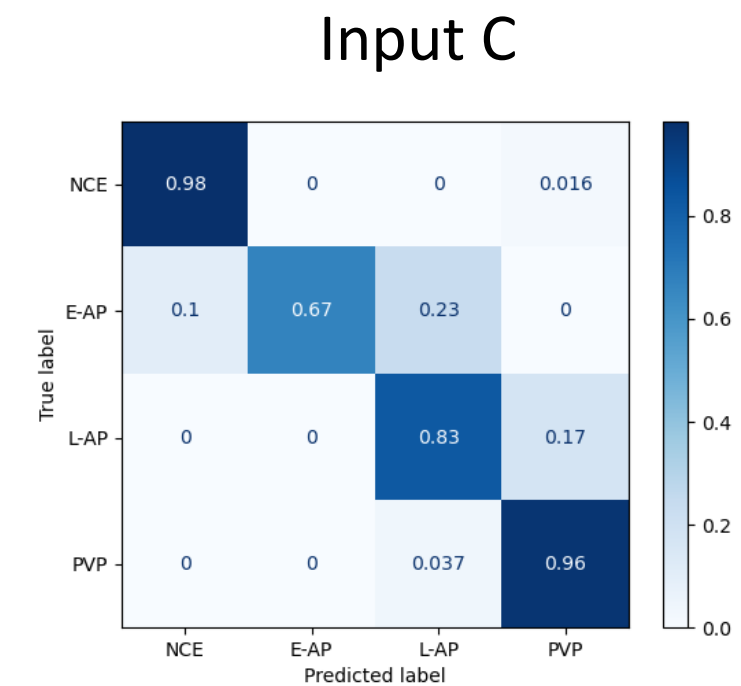

C) DT

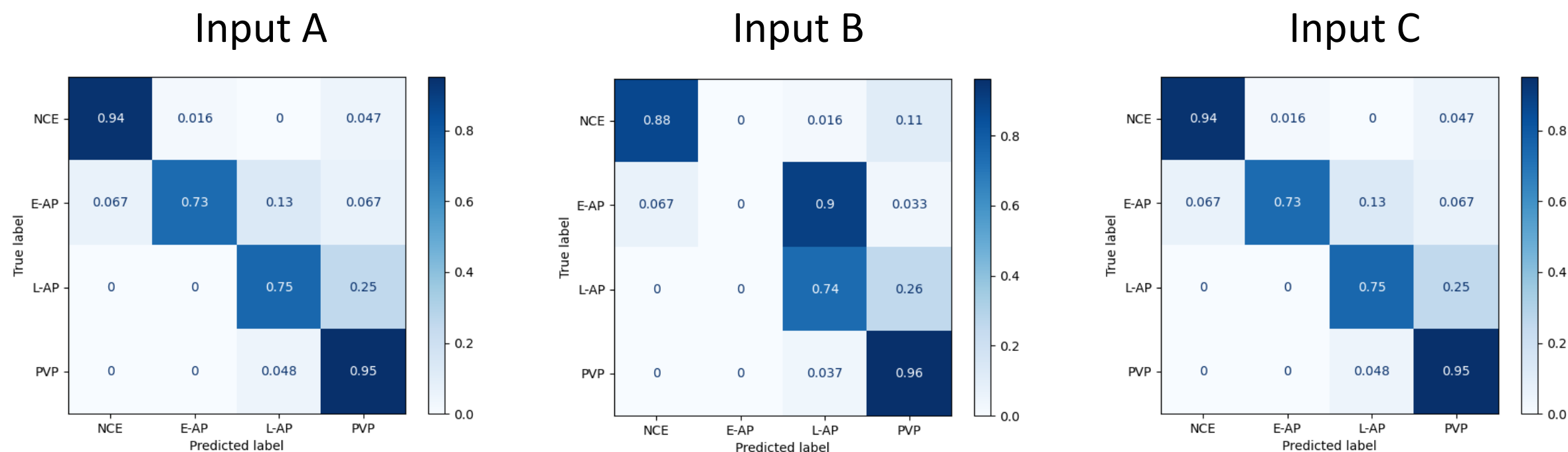

D) RF

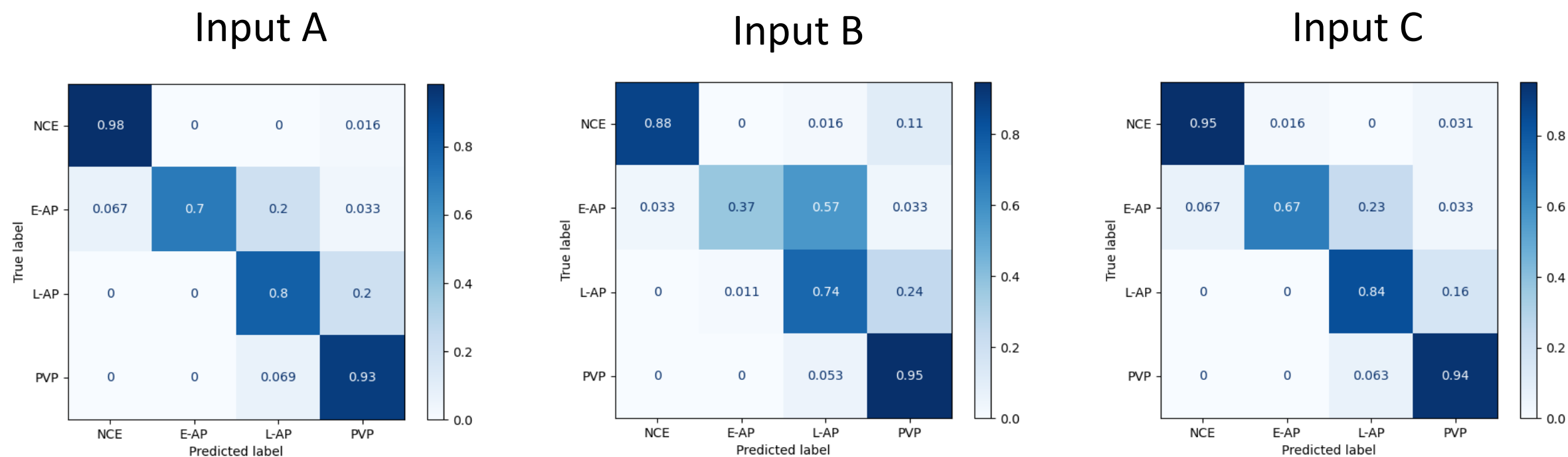

E) GBDT

Input A

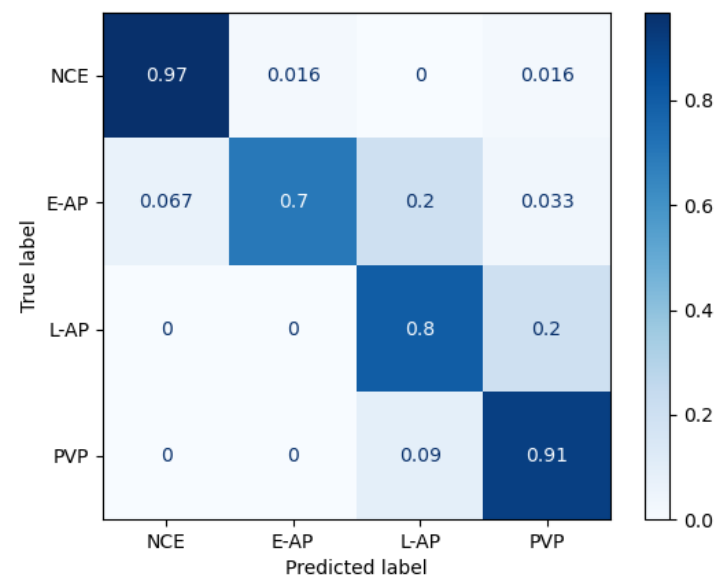

Input B

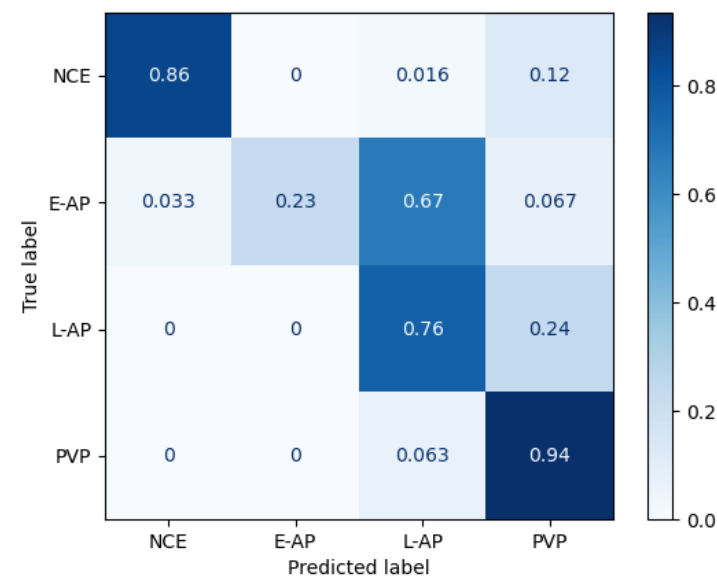

Input C

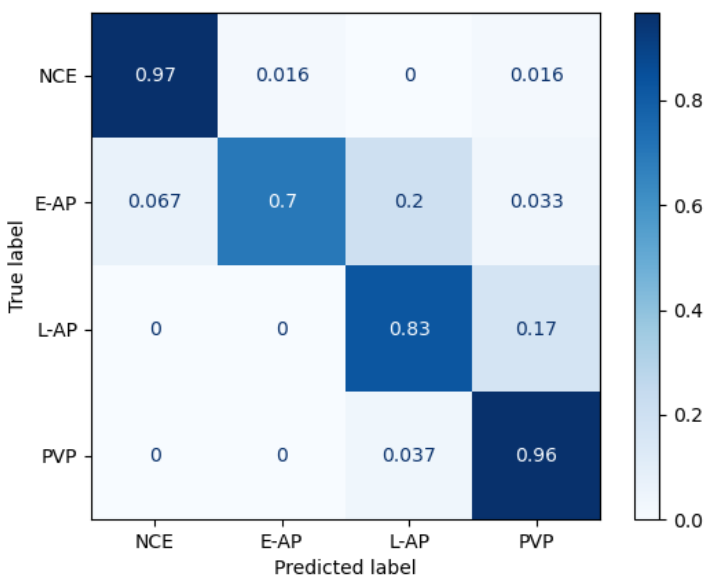

Supplement: S1 Fig — (A), (B), (C), (D) and (E) display the normalized confusion matrices for the logistic regression (LR), support vector machine (SVM), decision tree (DT), random forest (RF), and gradient-boosted decision tree (GBDT) models, respectively. Each matrix shows the performance on the same selected instance of cross-validation for models trained with Inputs A, B, and C from left to right. The greatest difficulty the models faced was in distinguishing early arterial phase (E-AP) from late arterial phase (L-AP) and late arterial phase (L-AP) from portal venous phase (PVP). Models trained using Input B have significantly greater difficulty correctly identifying E-AP than the models trained on the other two inputs. (PDF) [file pone.0294581.s001.pdf]

# A) LR OvR

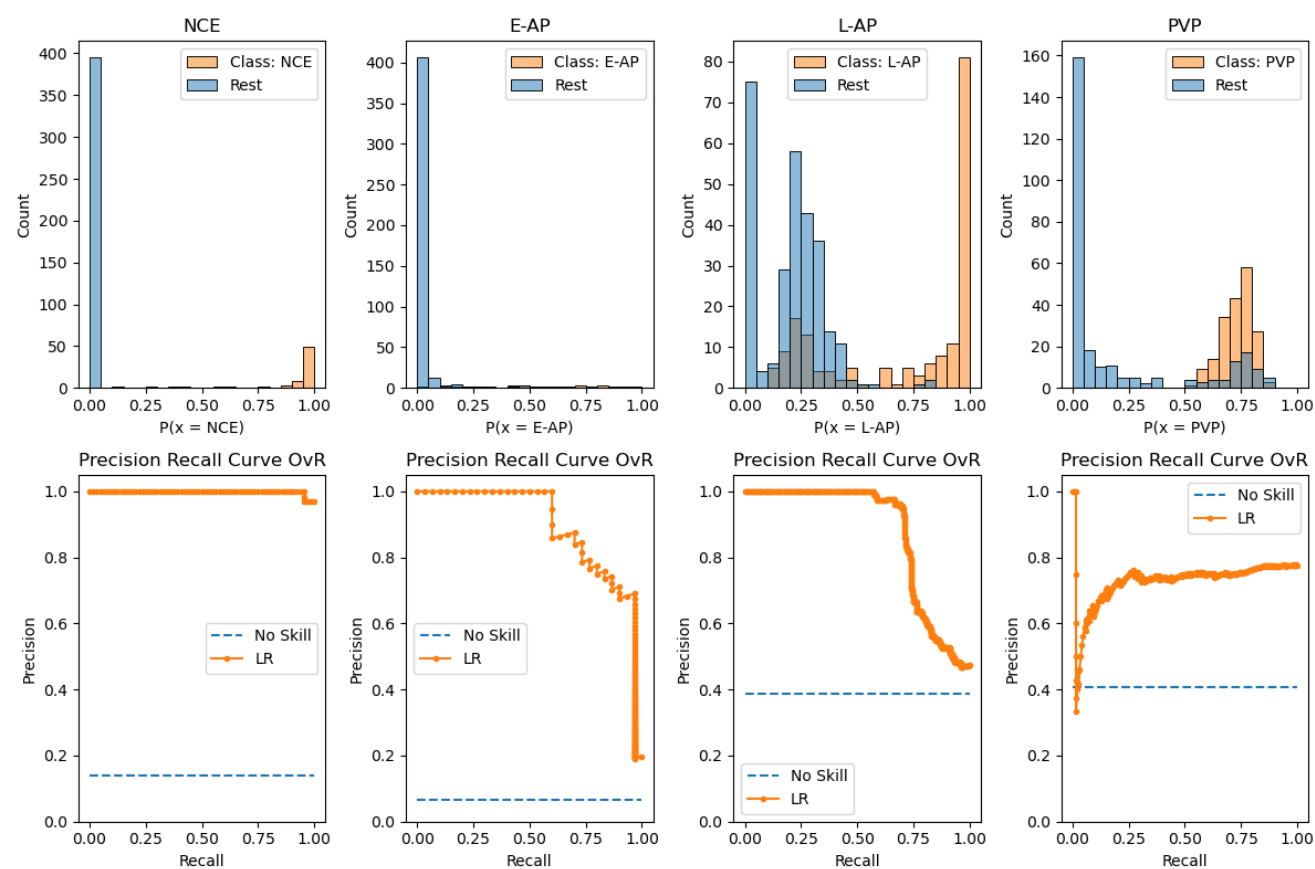

# LR OvO

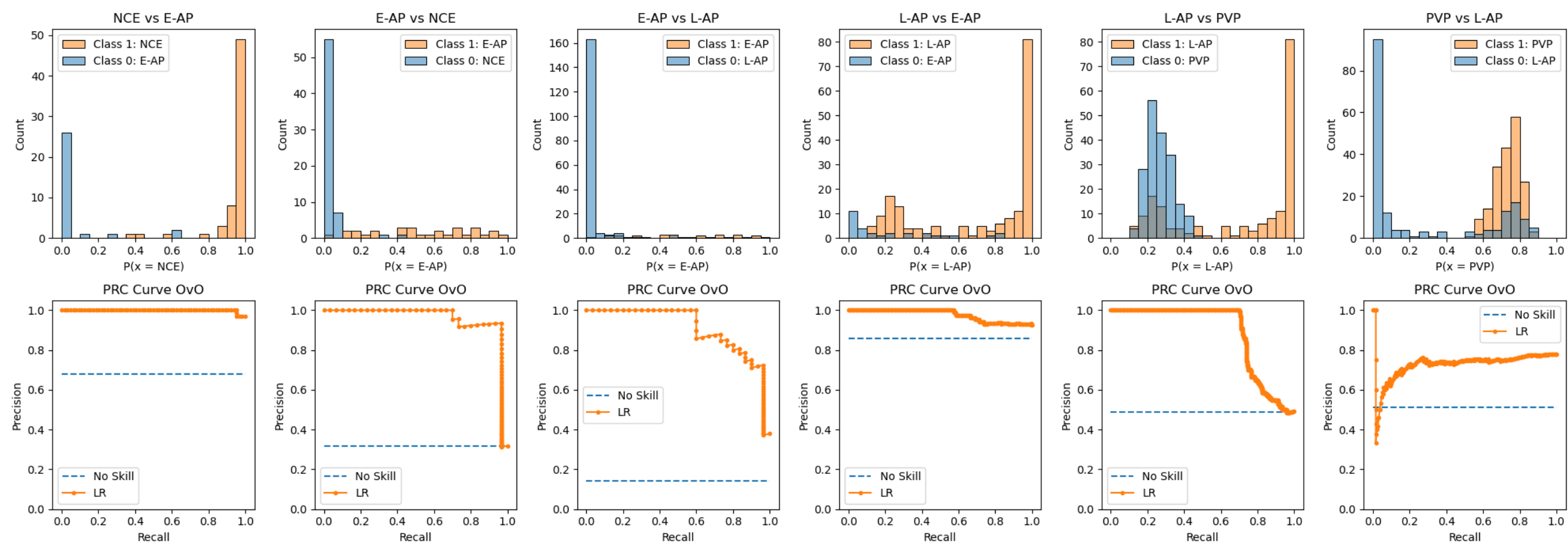

## B) SVM OvR

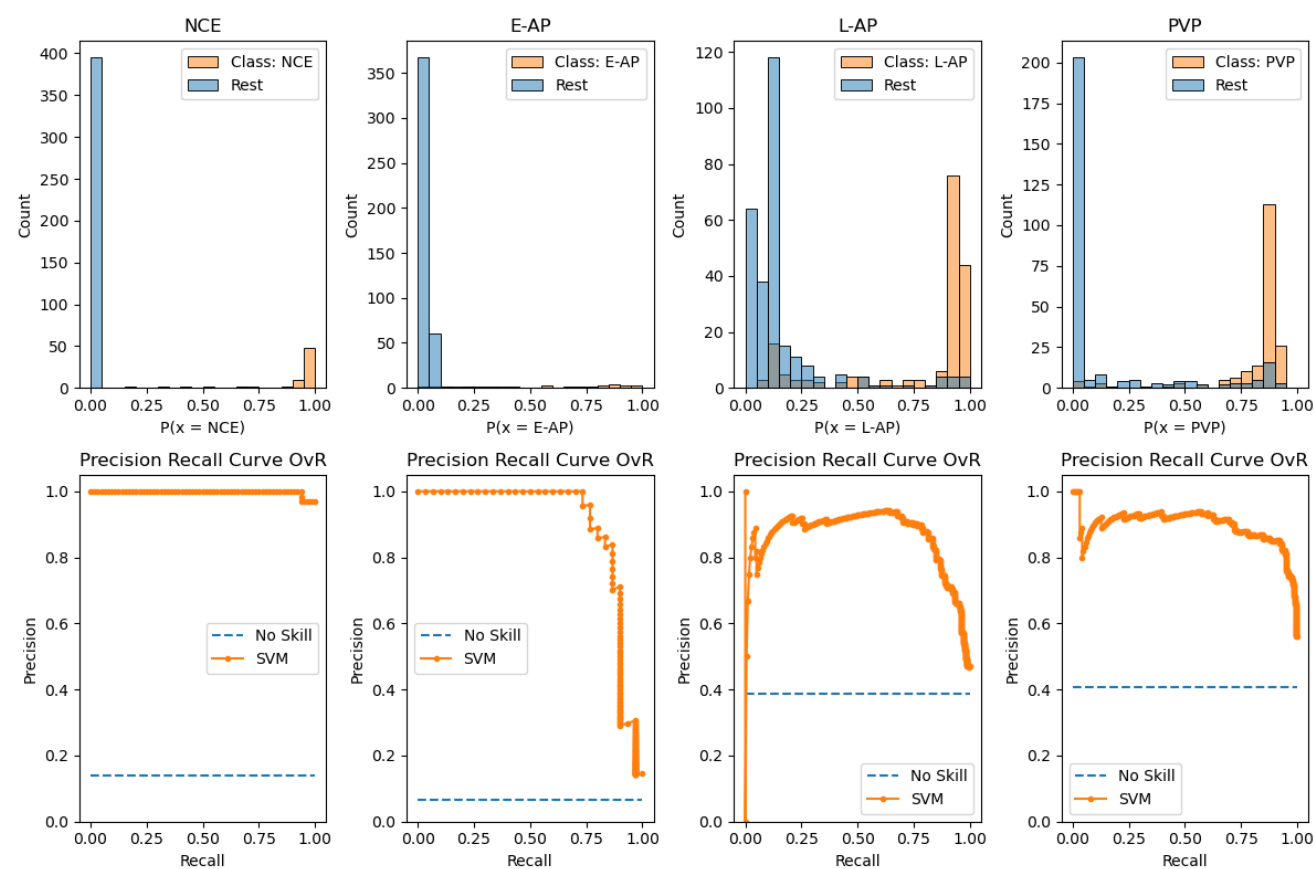

## SVM OvO

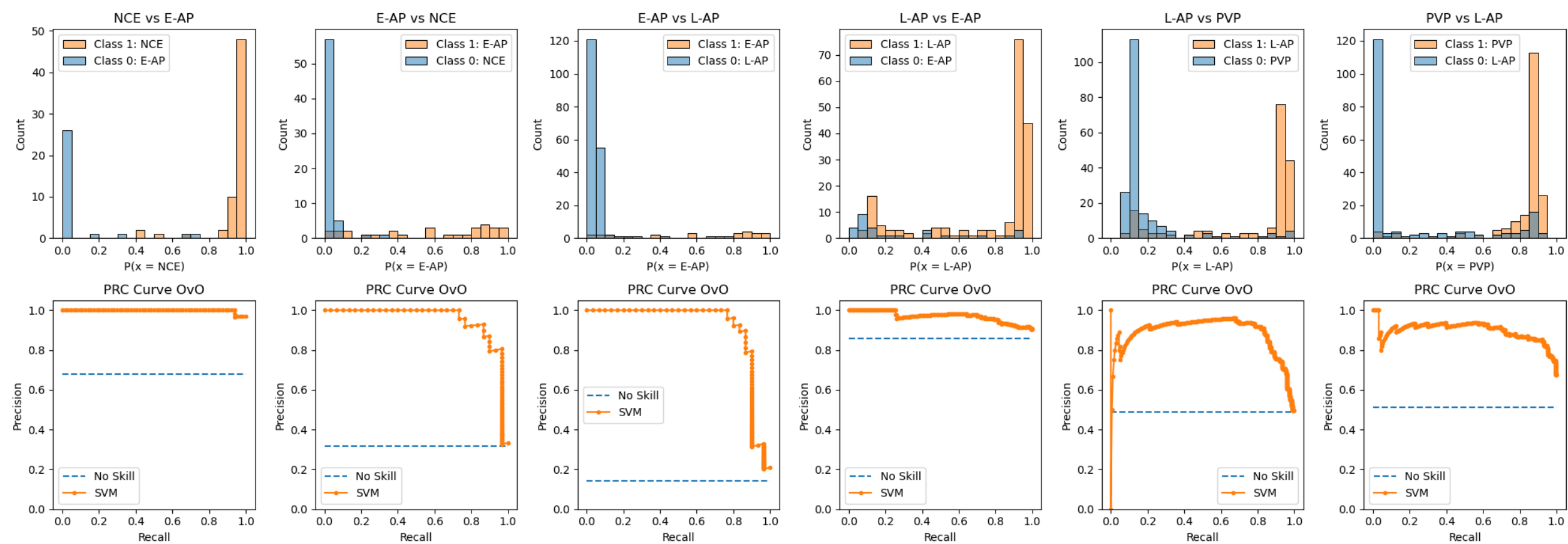

# C) DT OvR

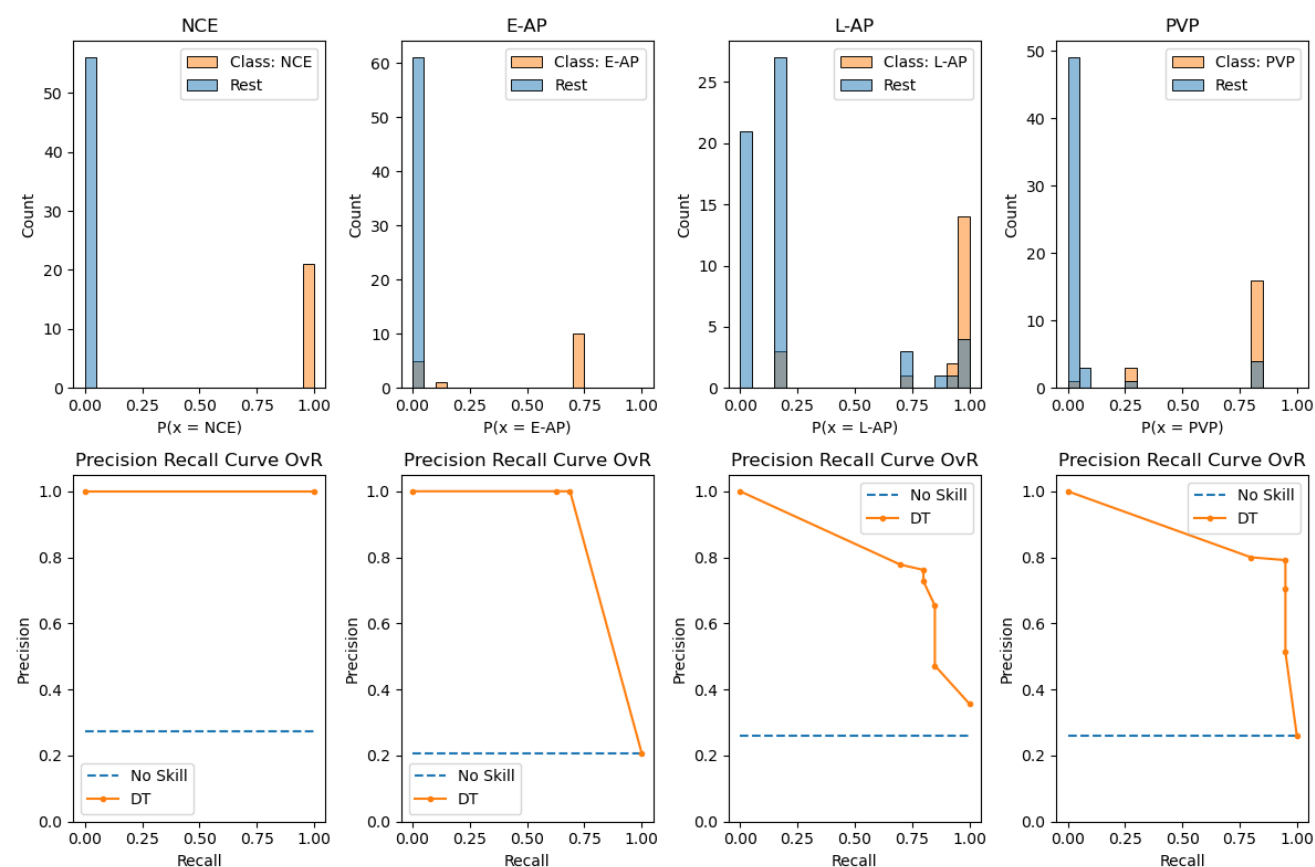

# DT OvO

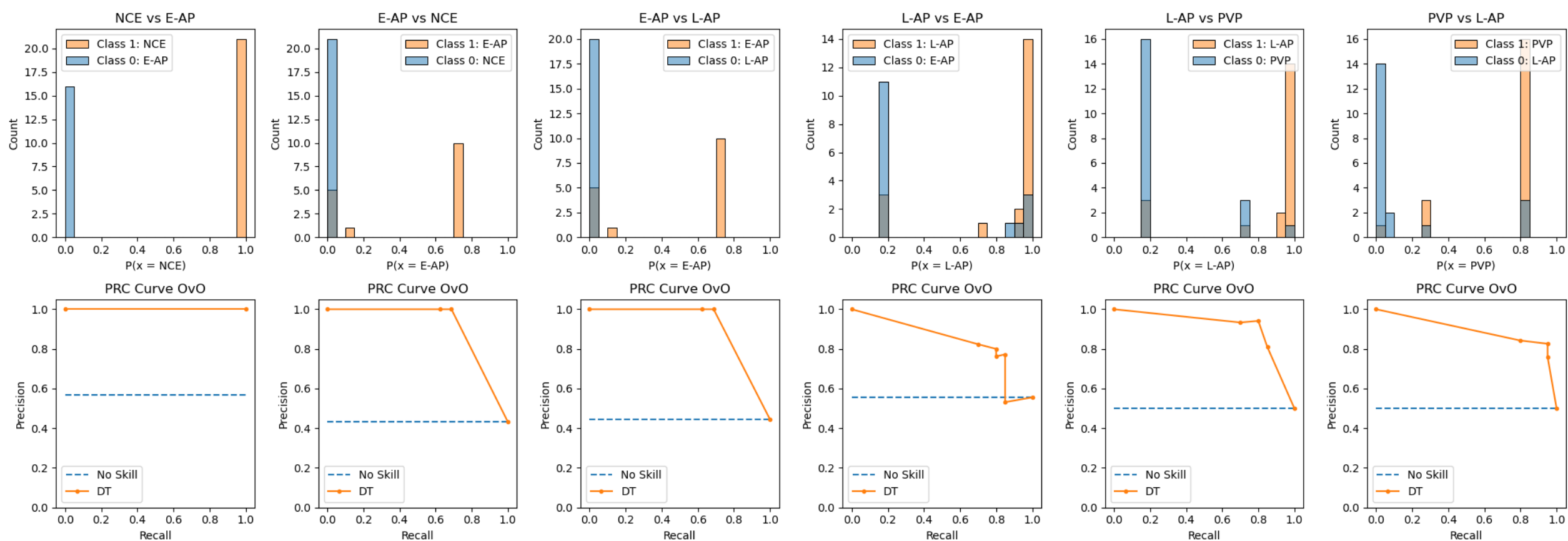

## D) RF OvR

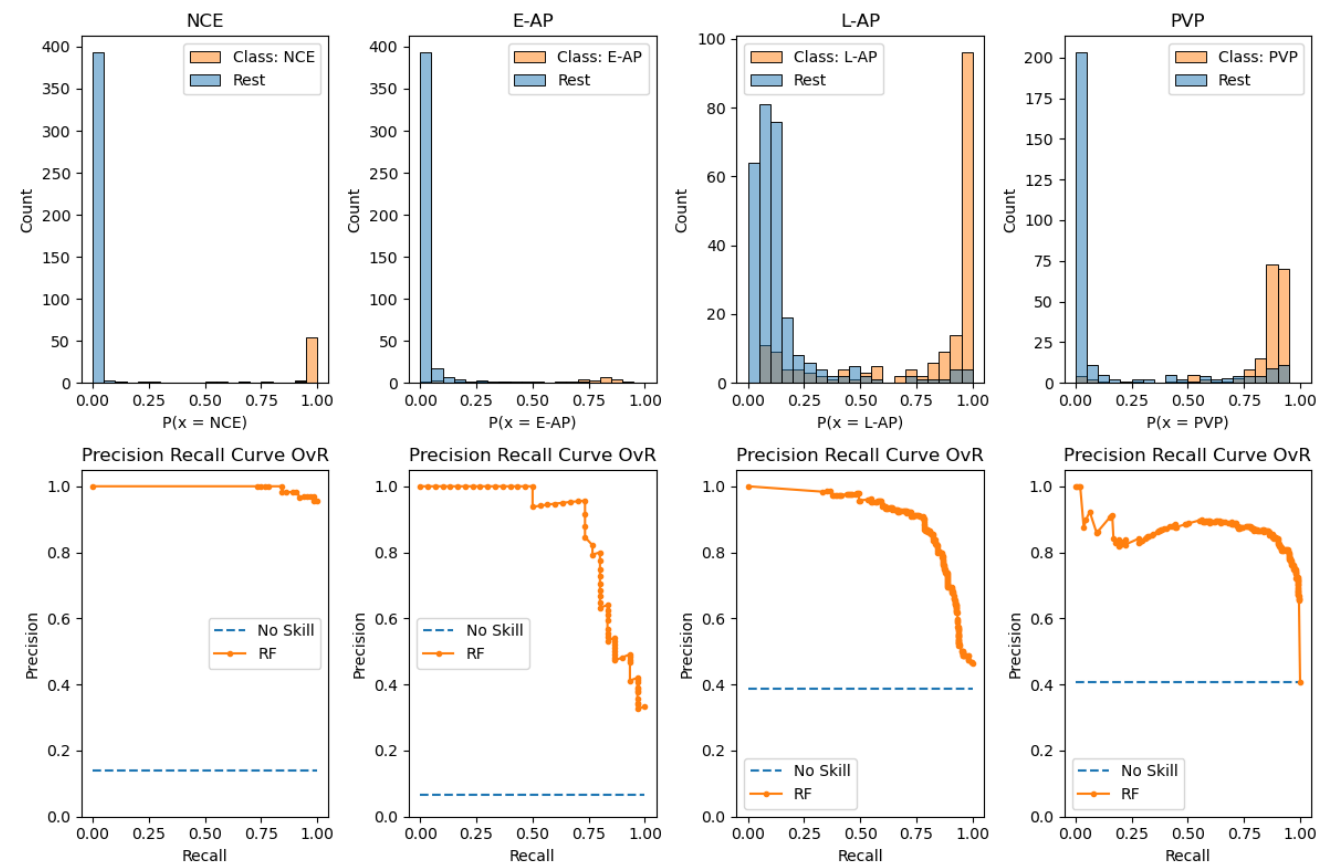

## RF OvO

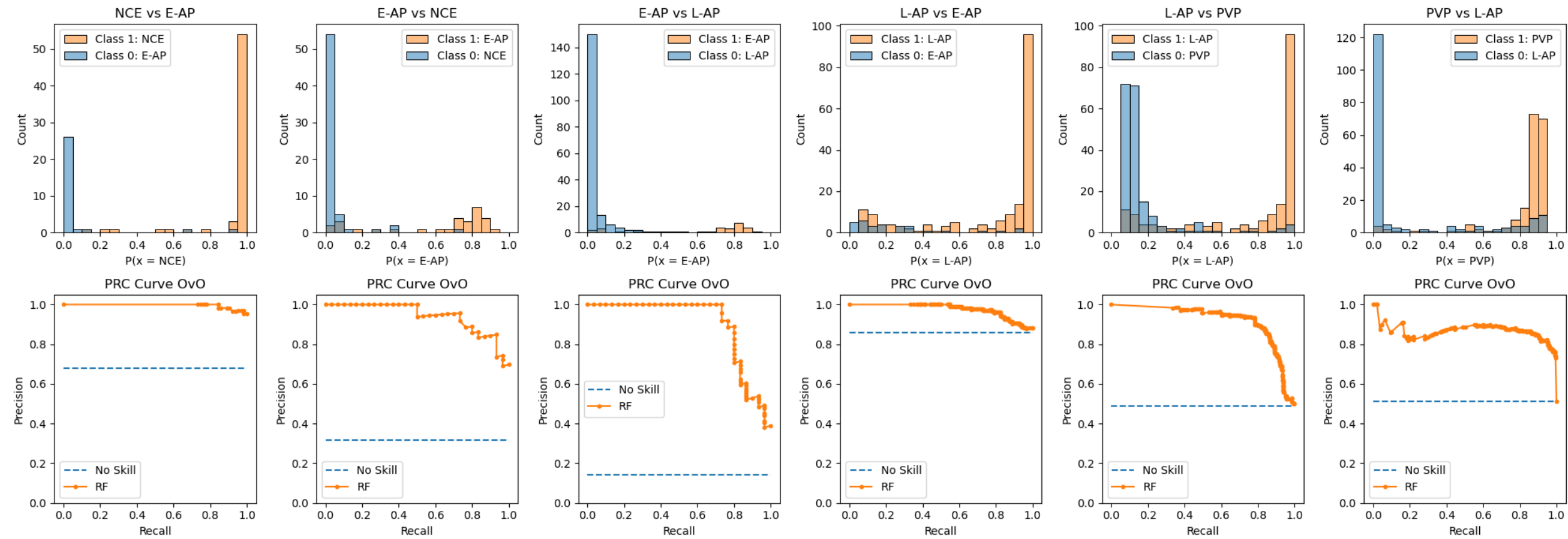

## E) GBDT OvR

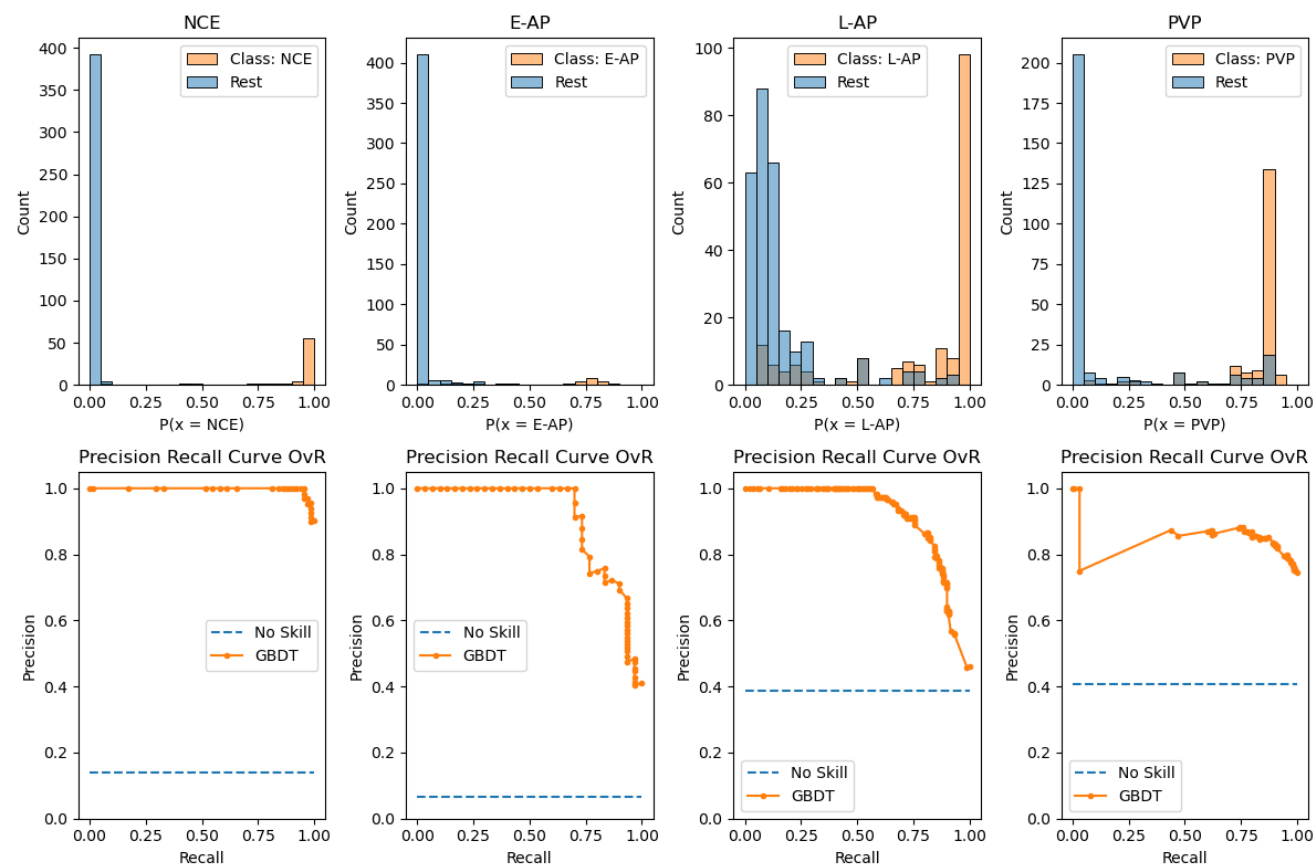

## GBDT OvO

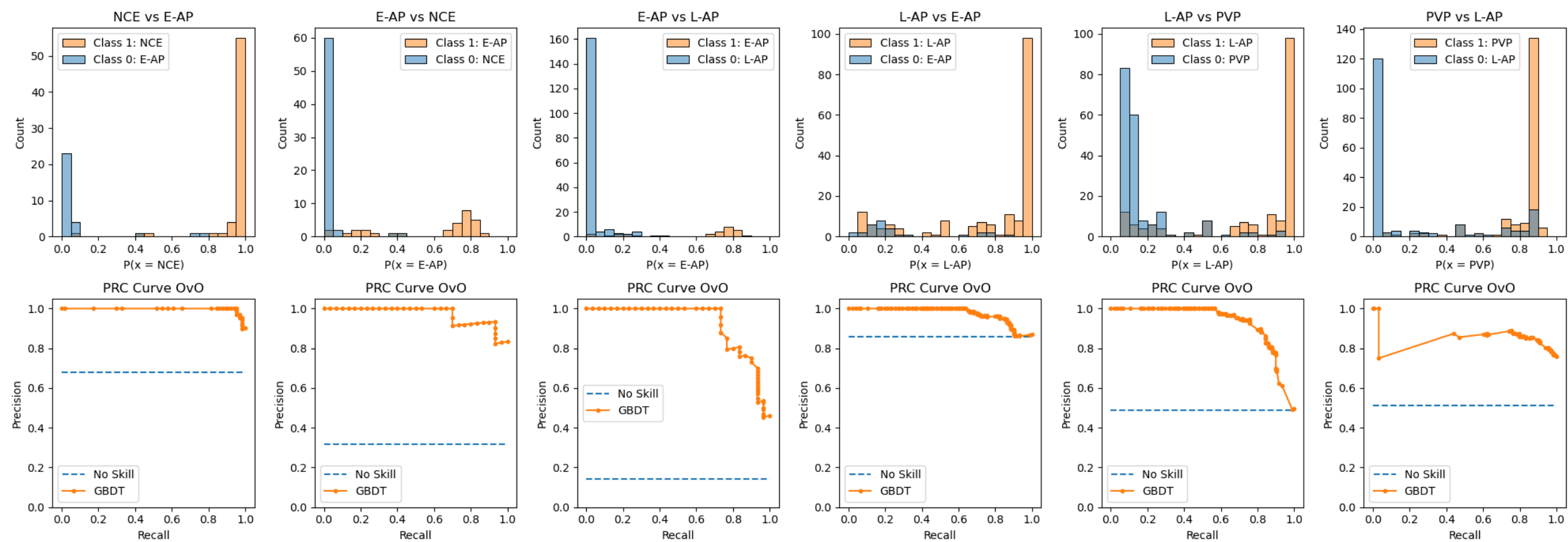

Supplement: S2 Fig — (A), (B), (C), (D), and (E) display the PRCs for the logistic regression (LR), support vector machine (SVM), decision tree (DT), random forest (RF), and gradient-boosted decision tree (GBDT) models, respectively. For each model, the graphs evaluated using a One vs. Rest (OvR) approach are shown on the top and a One vs. One (OvO) approach are shown on the (note that only the OvO PRCs for consecutive phases are shown). For each pair of graphs, the top row displays histograms of the probability calculated by the model that a given scan is the target label. If that probability is greater than 0.5, then the model will classify it as the target label. Scans with the target label with probabilities greater than 0.5 and scans not of the target label (“Rest” or “Class 0”) with probabilities less than 0.5 are correctly classified. The bottom row of graphs in each pair displays the PRCs. A no-skill classifier is displayed as a horizontal line of the number of scans of the target label divided by the total number of scans. (PDF) [file pone.0294581.s002.pdf]

# A) LR OvR

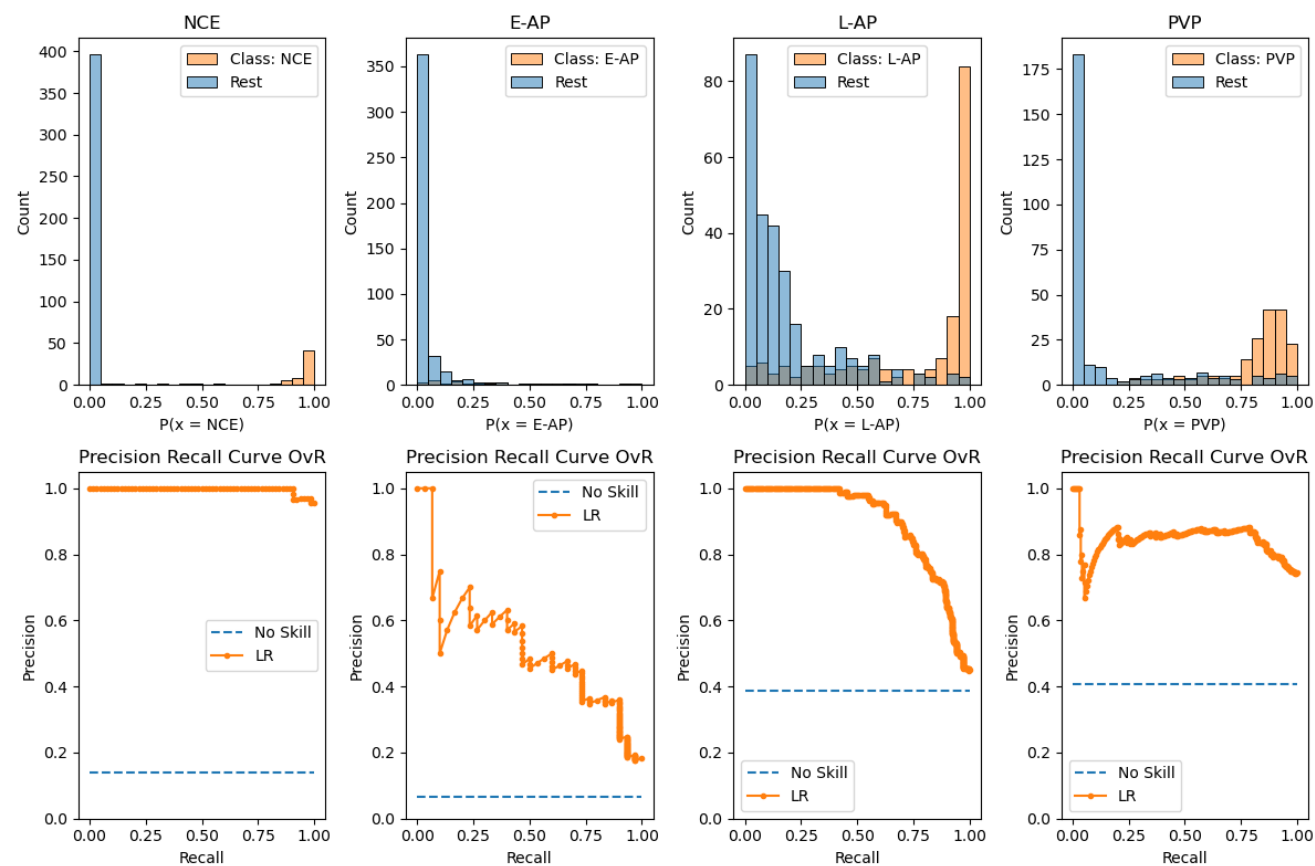

# LR OvO

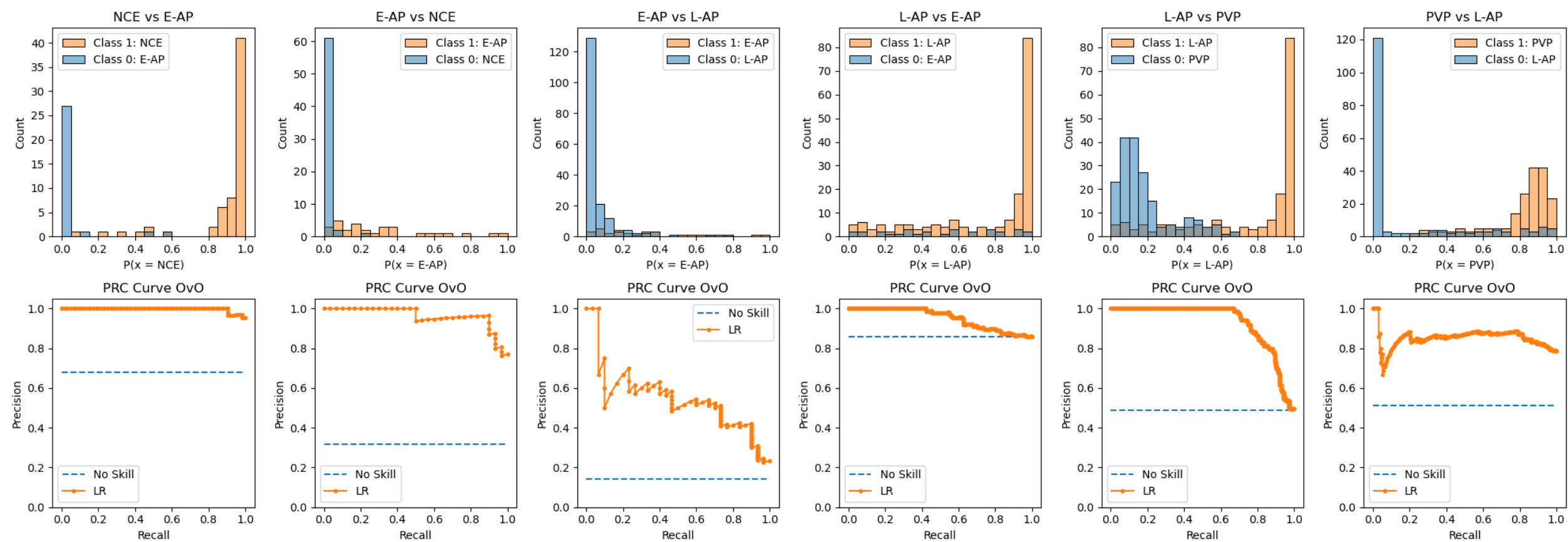

## B) SVM OvR

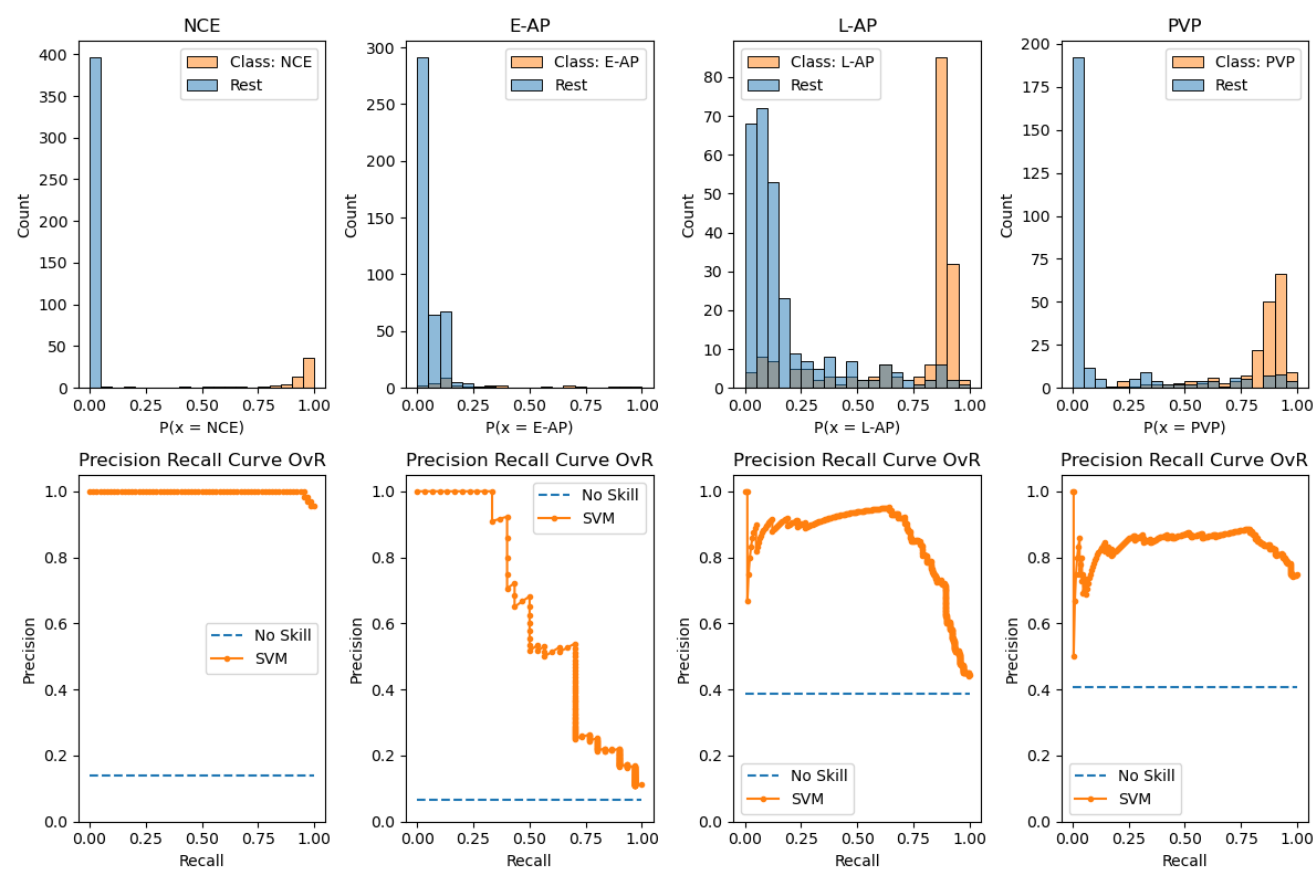

## SVM OvO

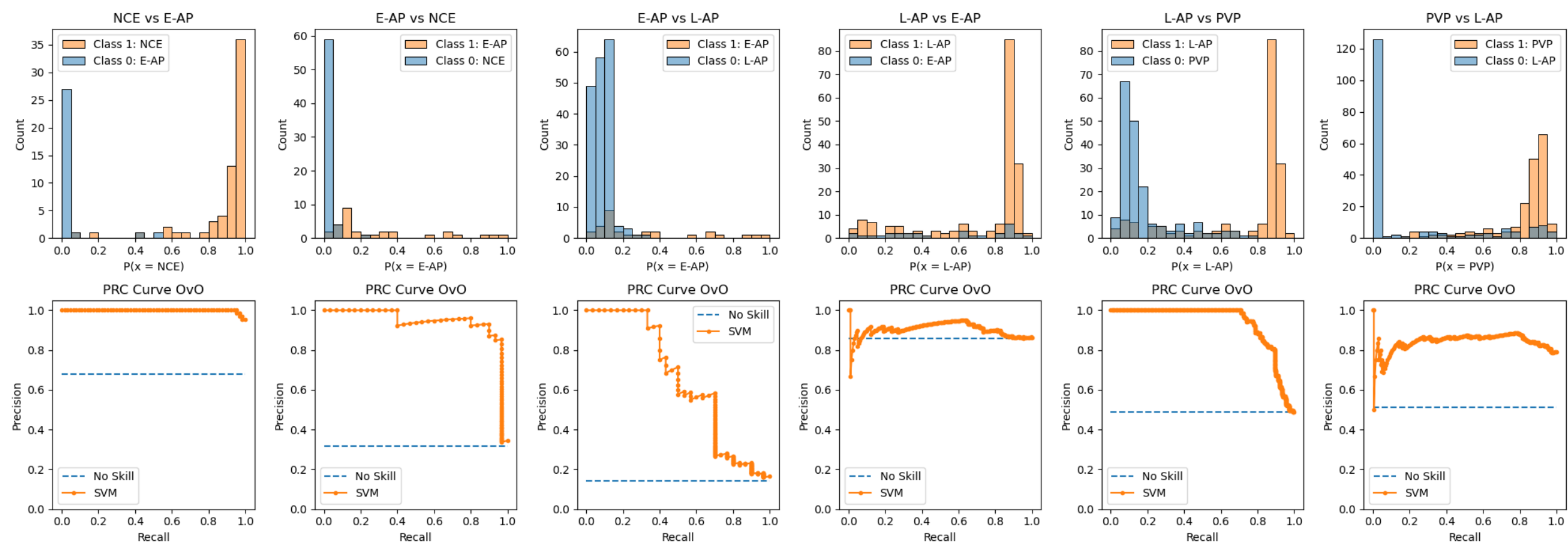

# C) DT OvR

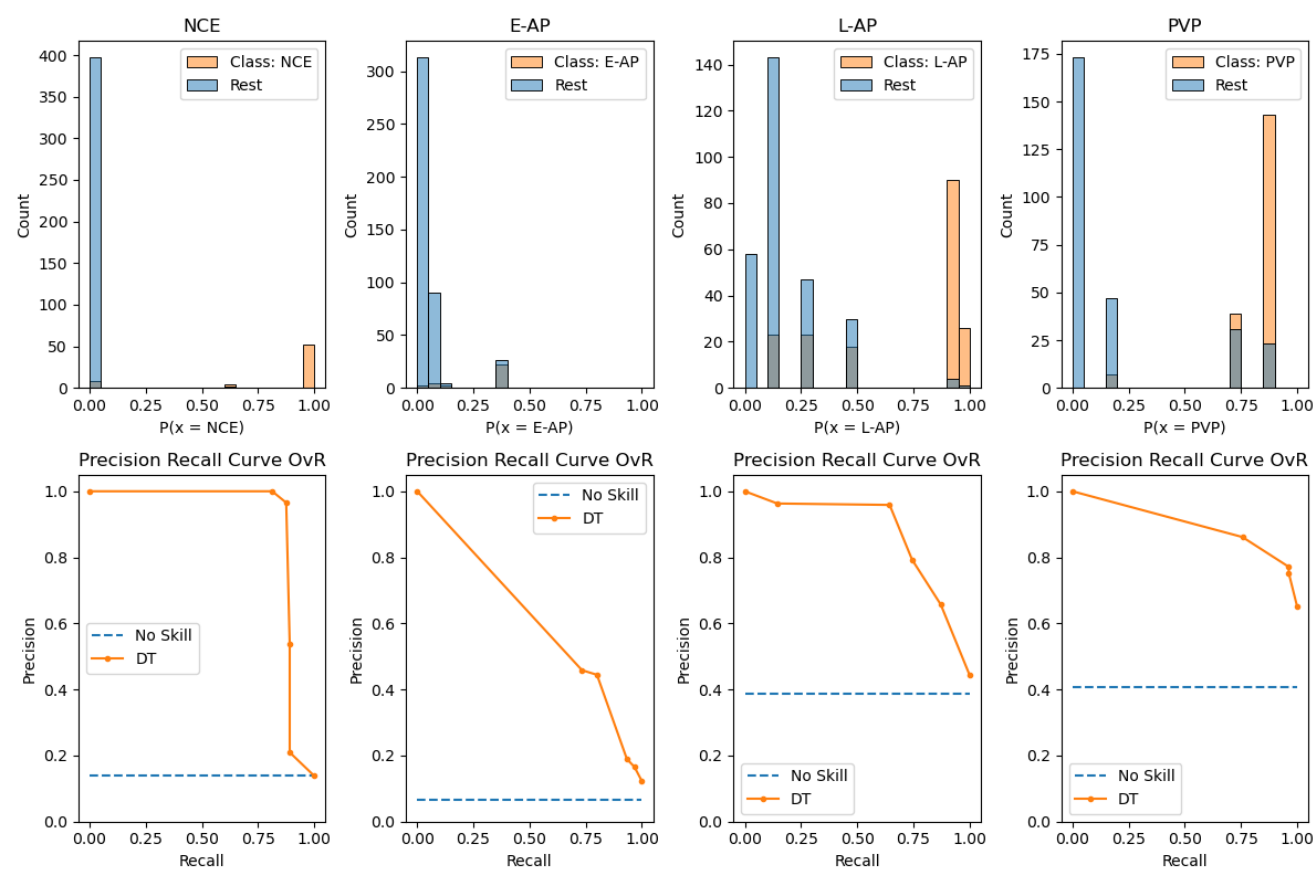

# DT OvO

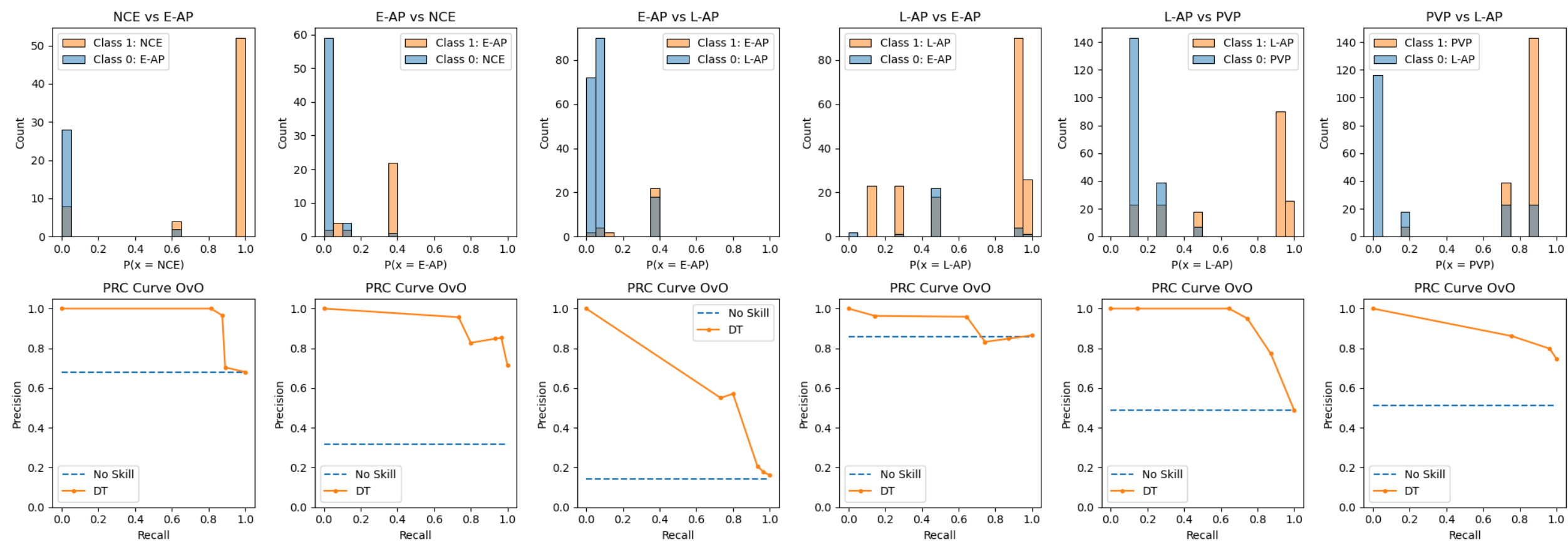

## D) RF OvR

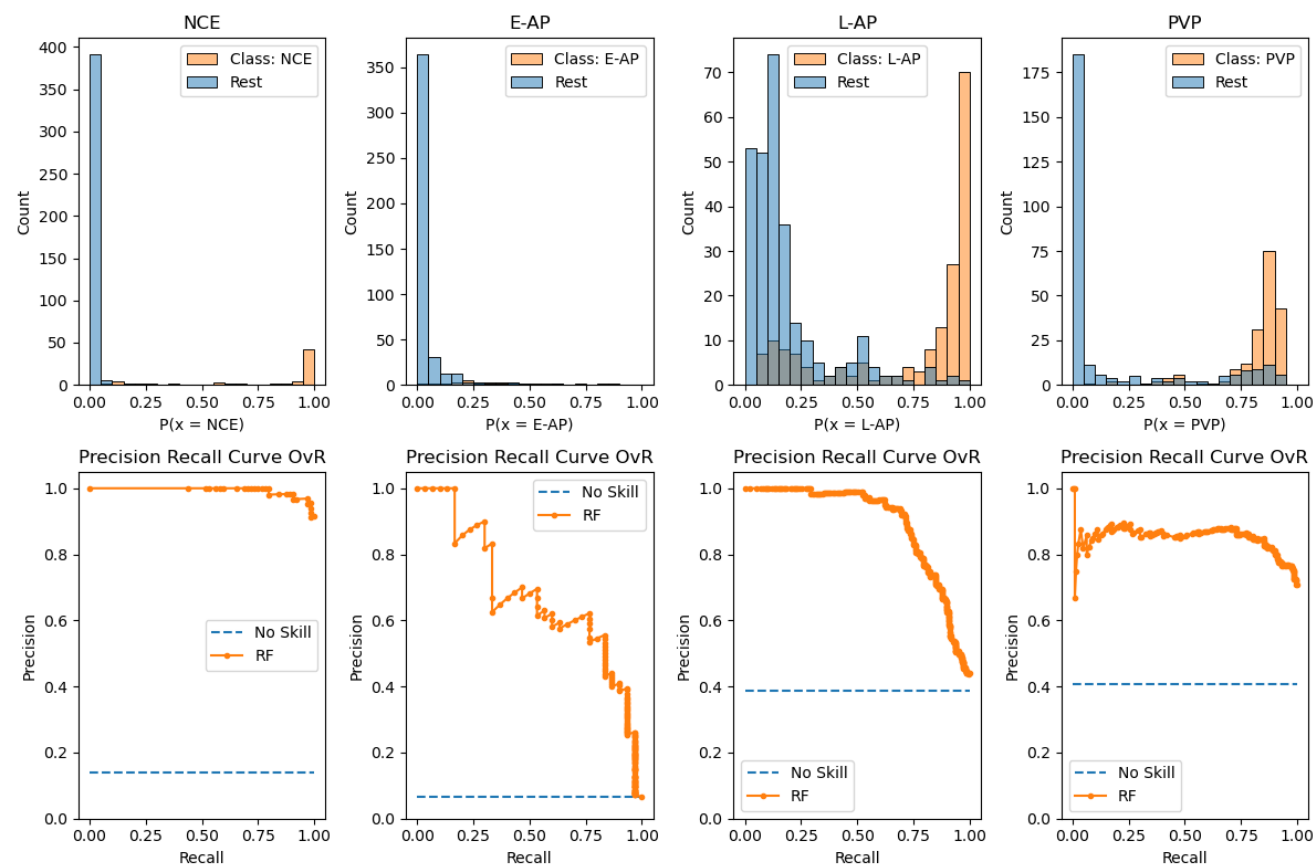

## RF OvO

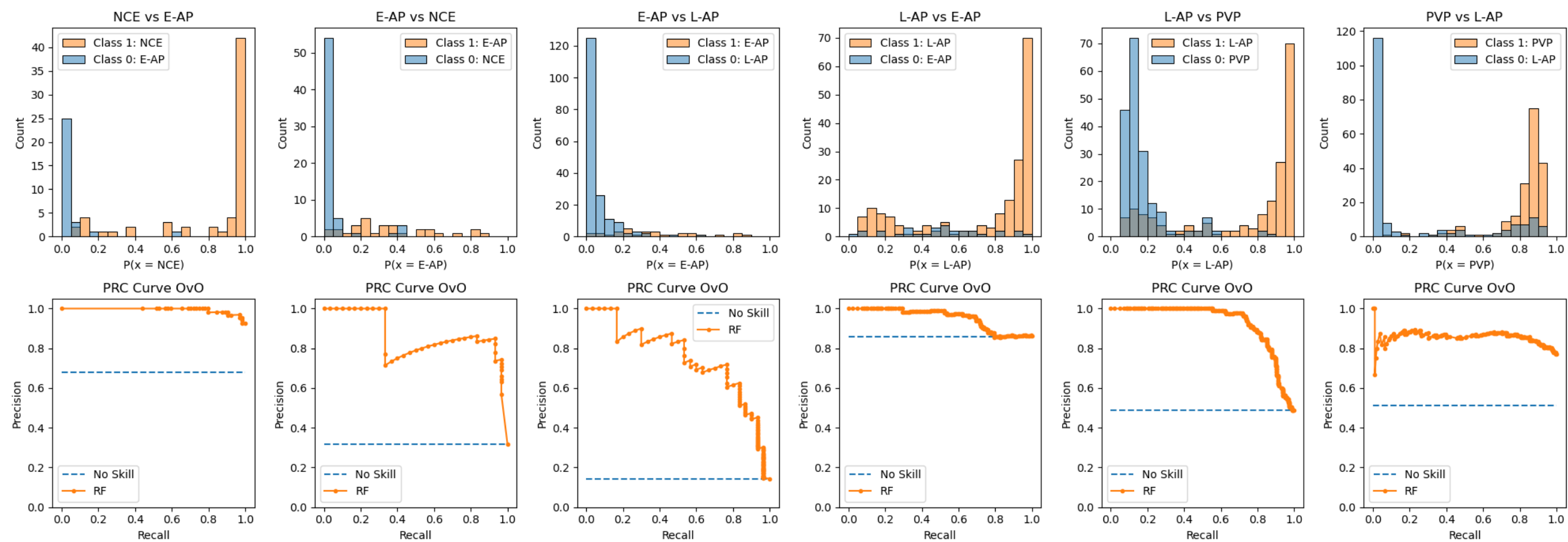

## E) GBDT OvR

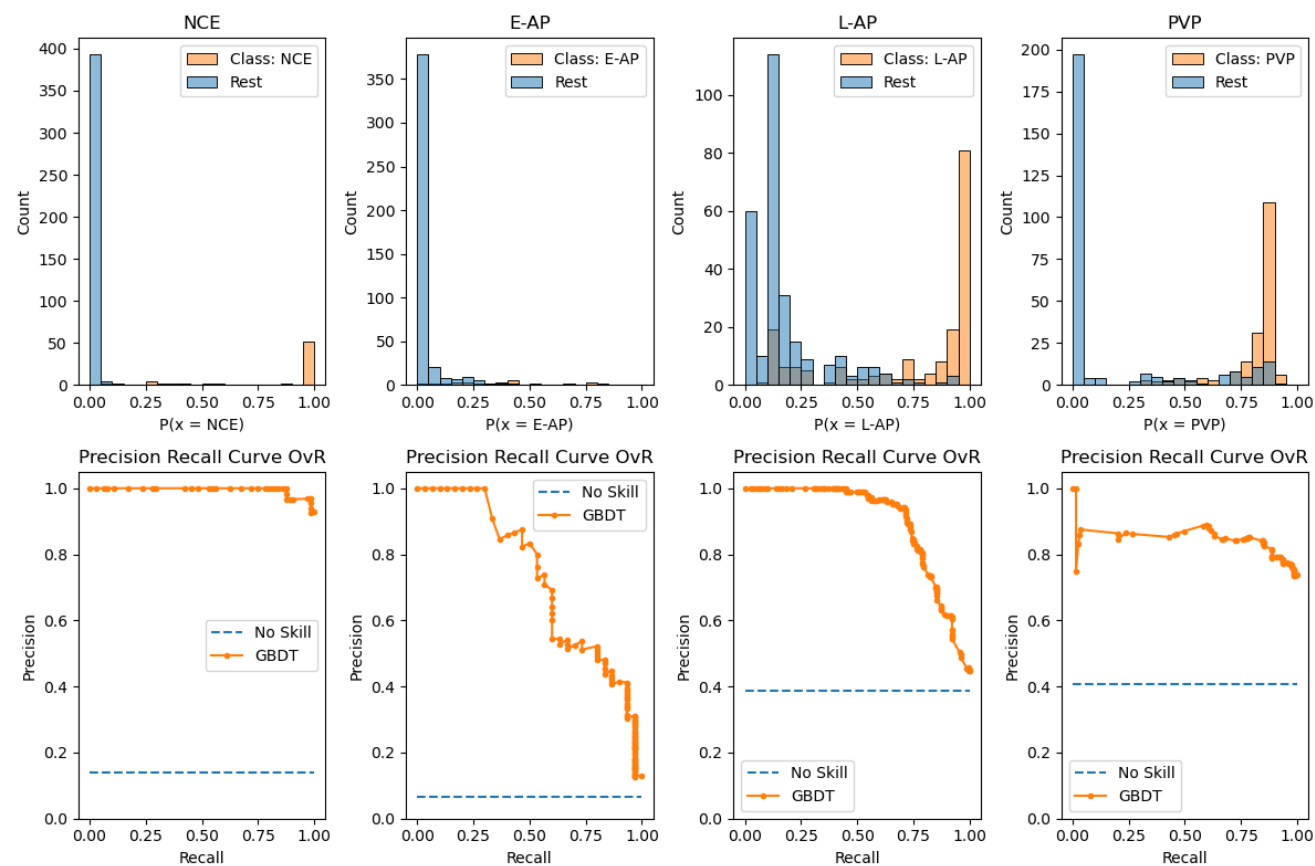

## GBDT OvO

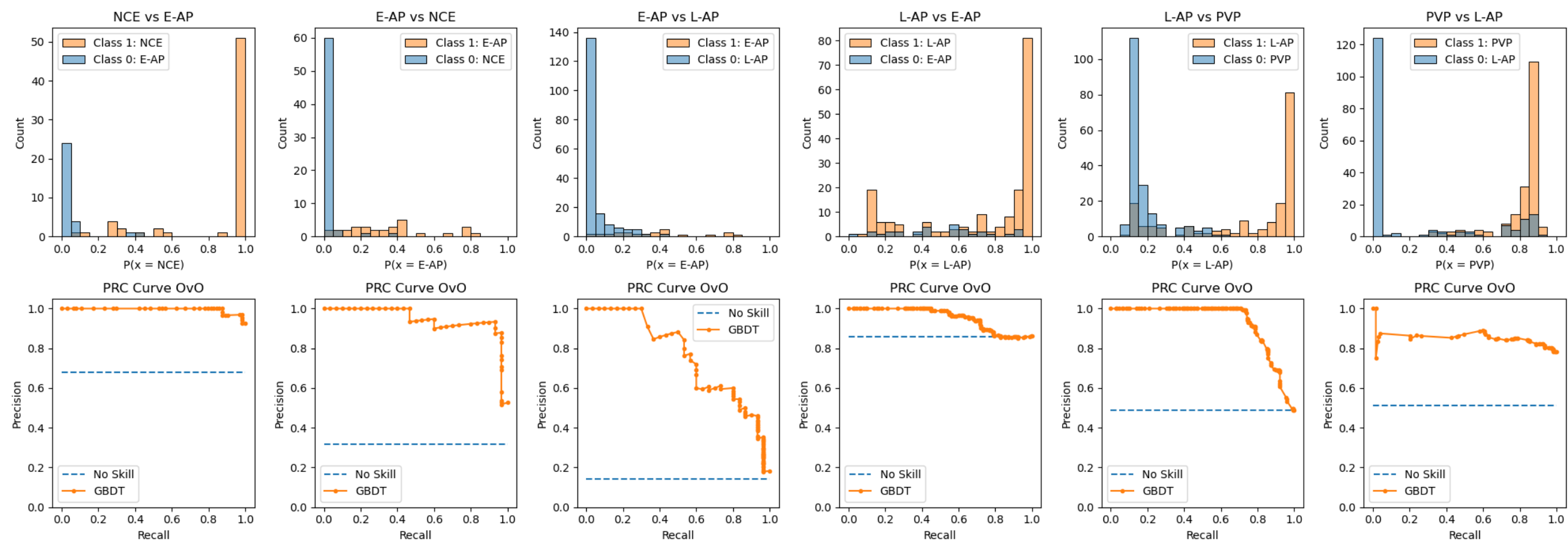

Supplement: S3 Fig — (A), (B), (C), (D) and (E) display the PRCs for the logistic regression (LR), support vector machine (SVM), decision tree (DT), random forest (RF), and gradient-boosted decision tree (GBDT) models, respectively. For each model, the graphs evaluated using a One vs. Rest (OvR) approach are shown on the top and a One vs. One (OvO) approach are shown on the bottom (note that only the OvO PRCs for consecutive phases are shown). See S2 Fig for more details on their interpretation. (PDF) [file pone.0294581.s003.pdf]

# A) LR OvR

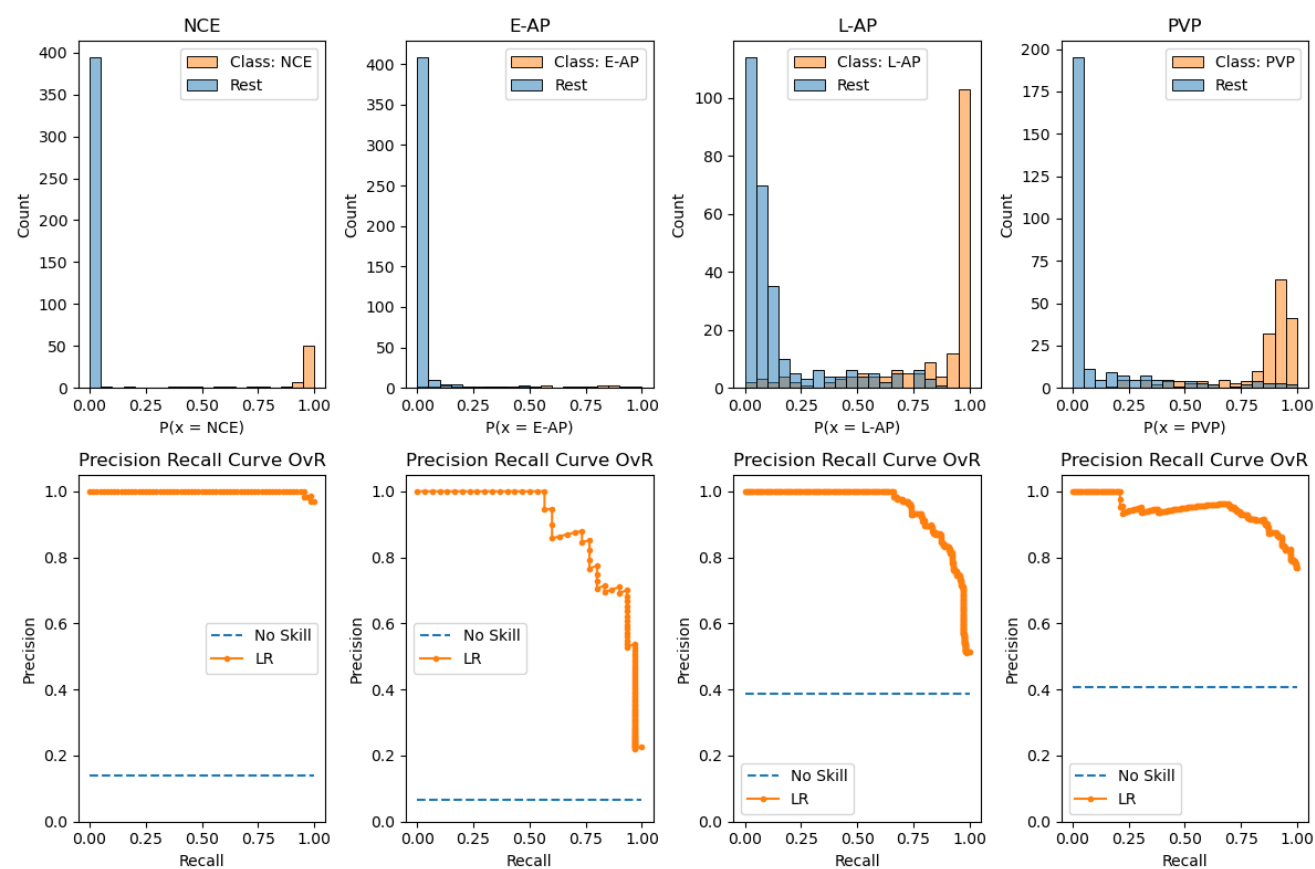

# LR OvO

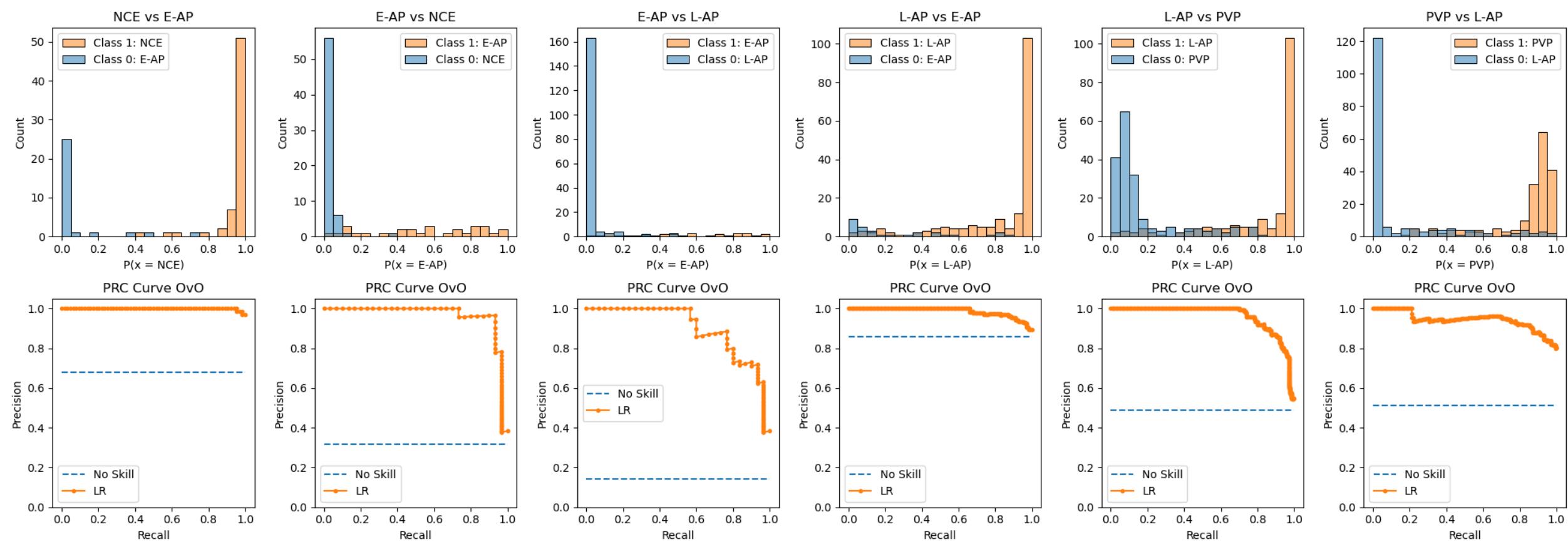

## B) SVM OvR

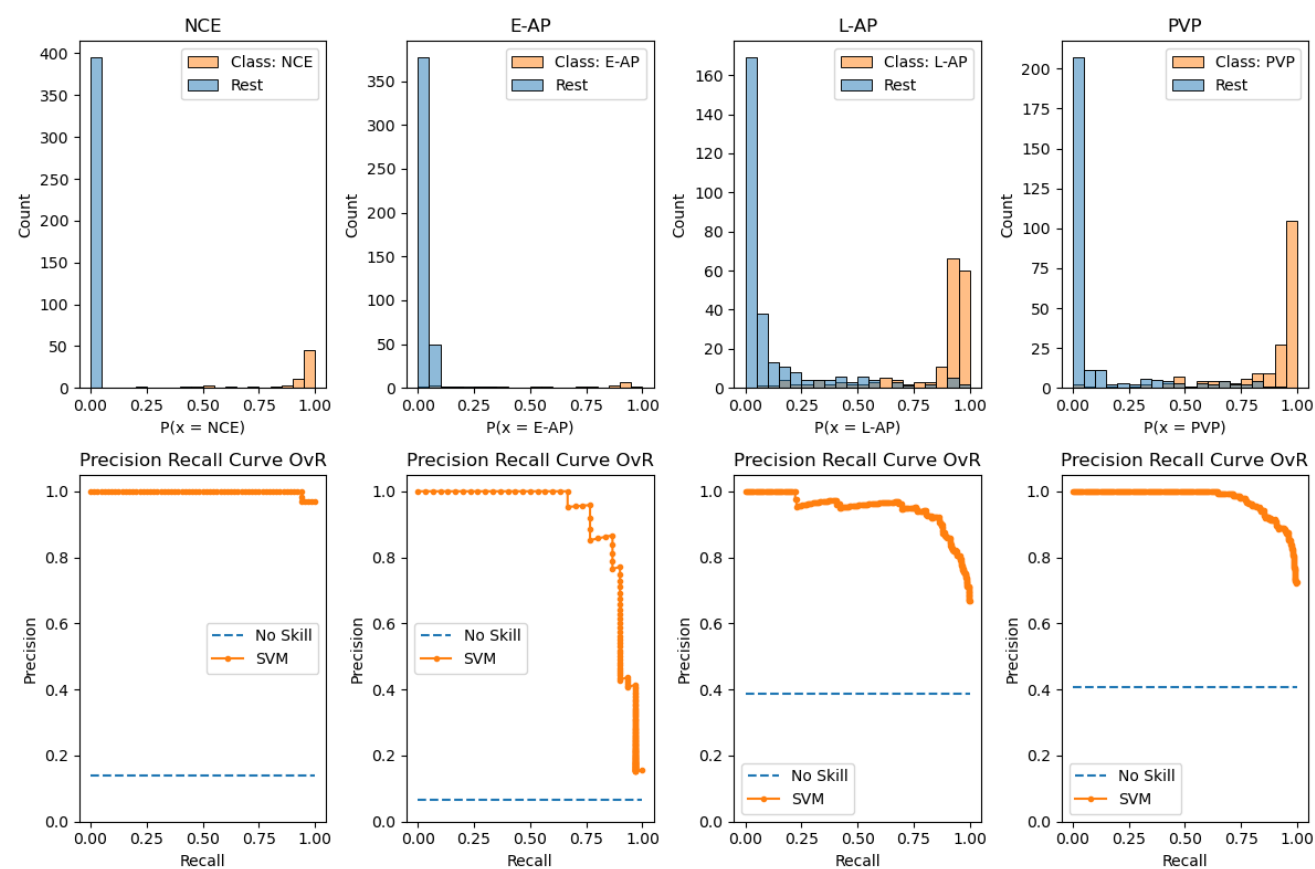

## SVM OvO

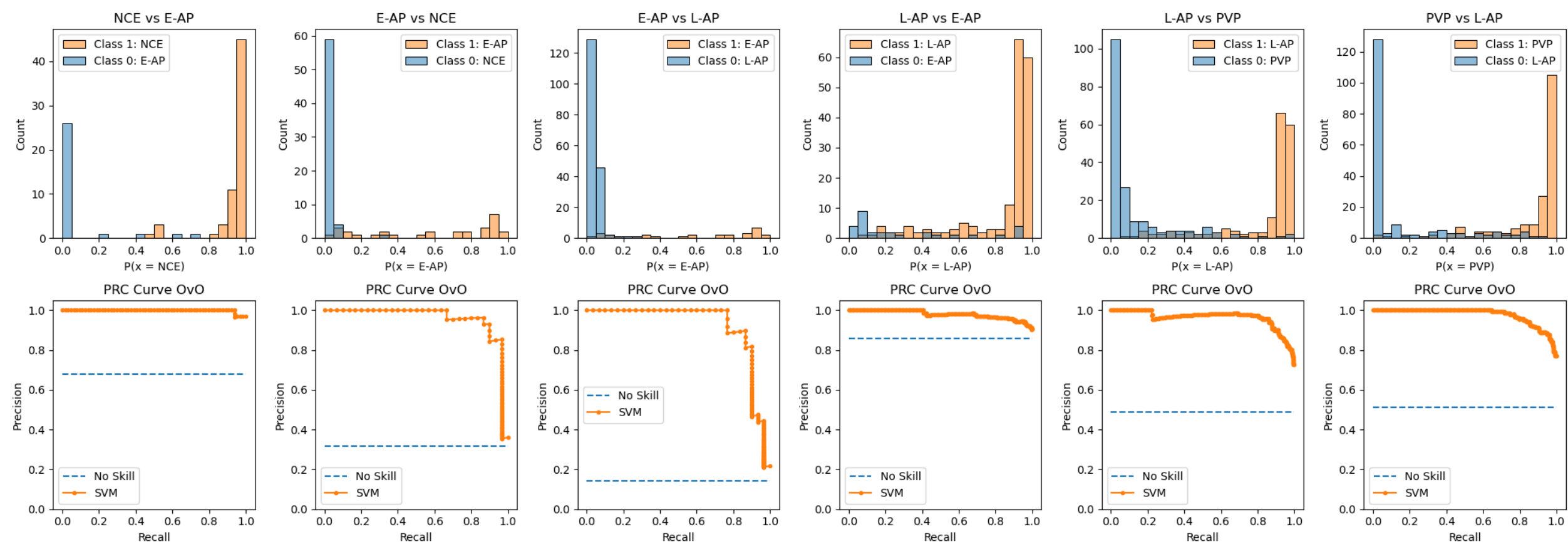

# C) DT OvR

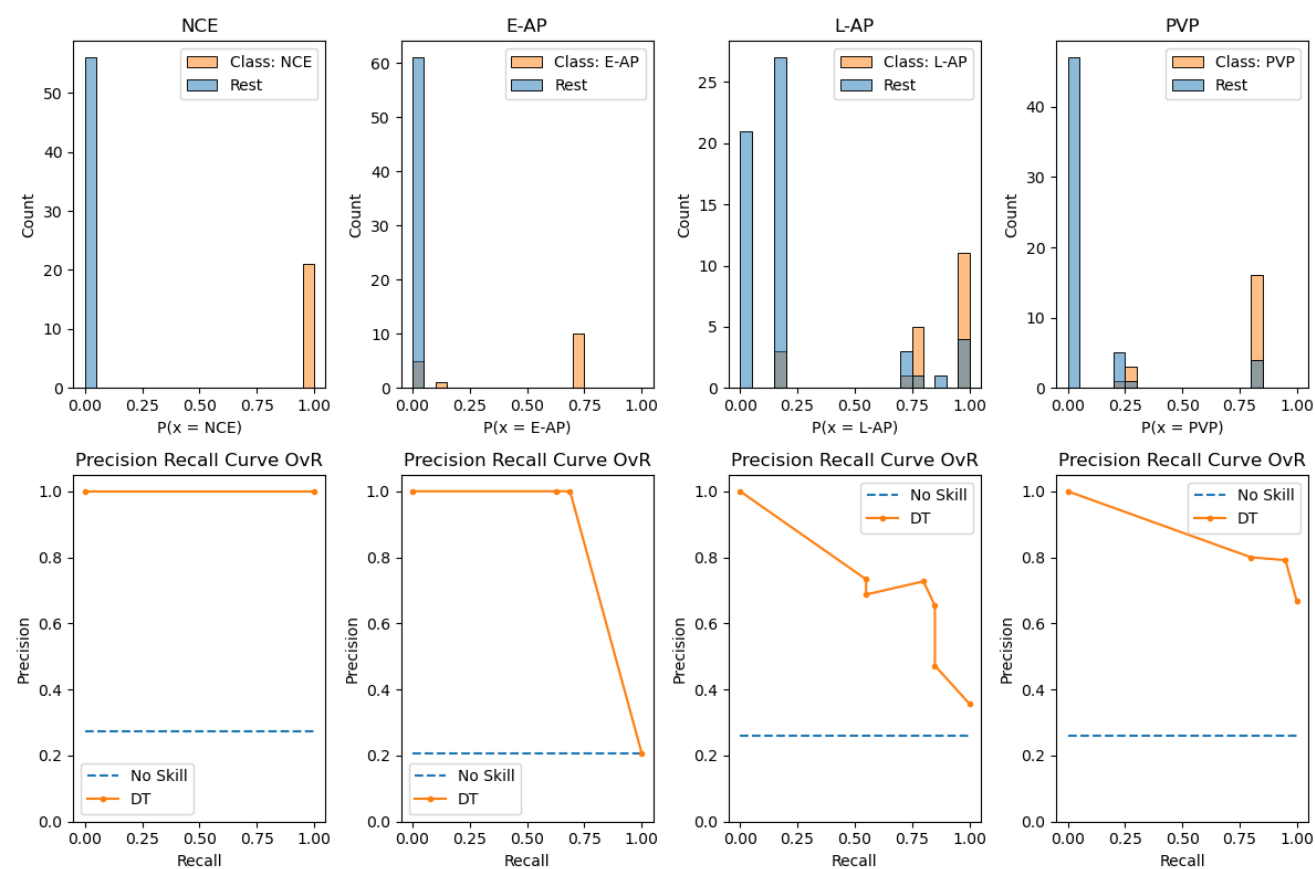

# DT OvO

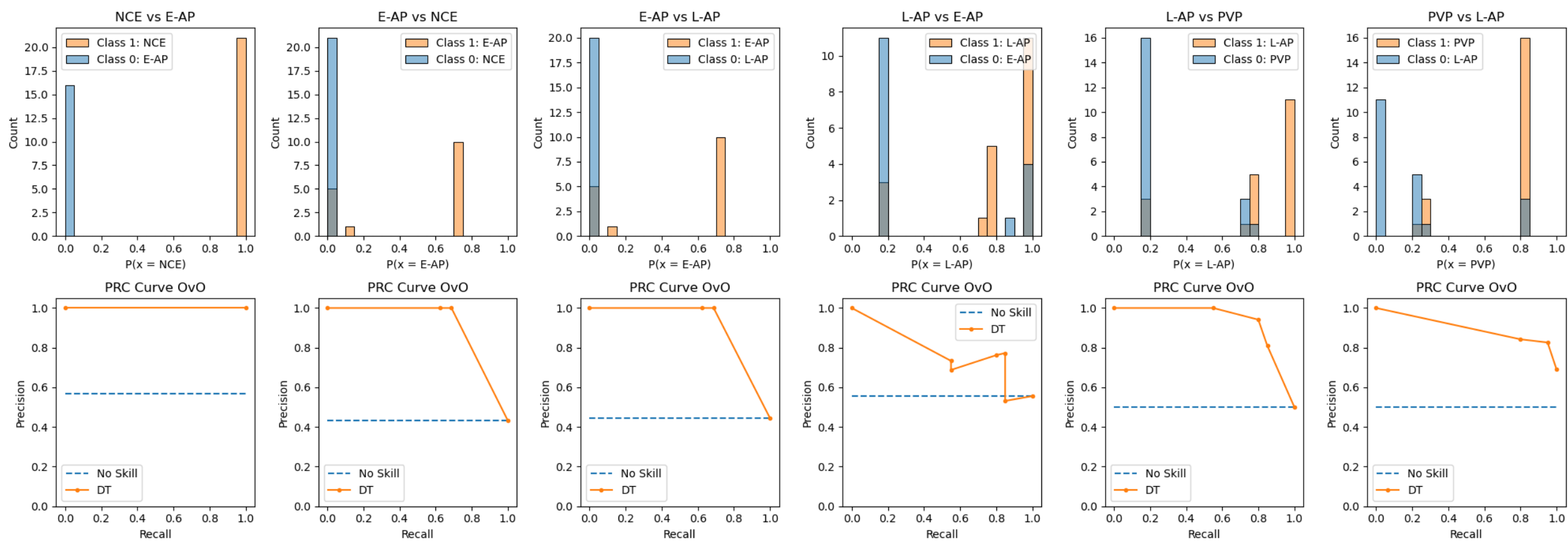

## D) RF OvR

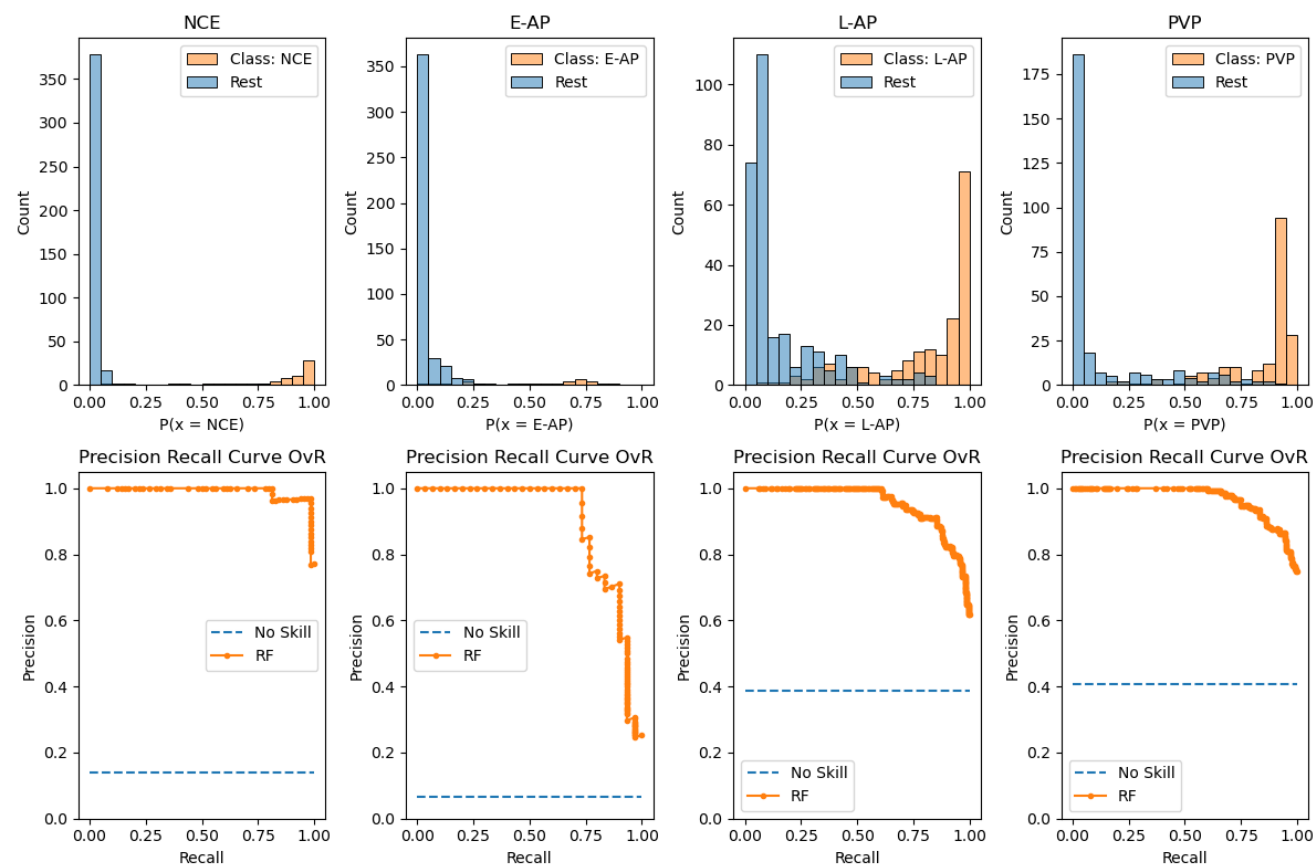

## RF OvO

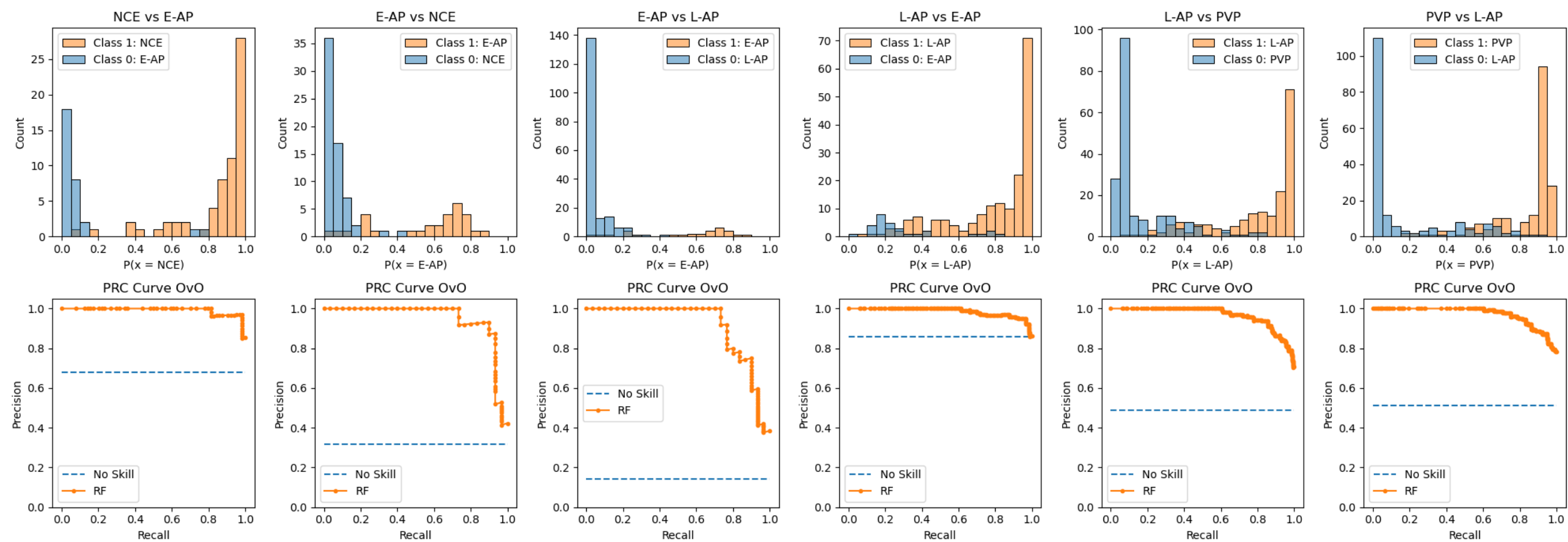

## E) GBDT OvR

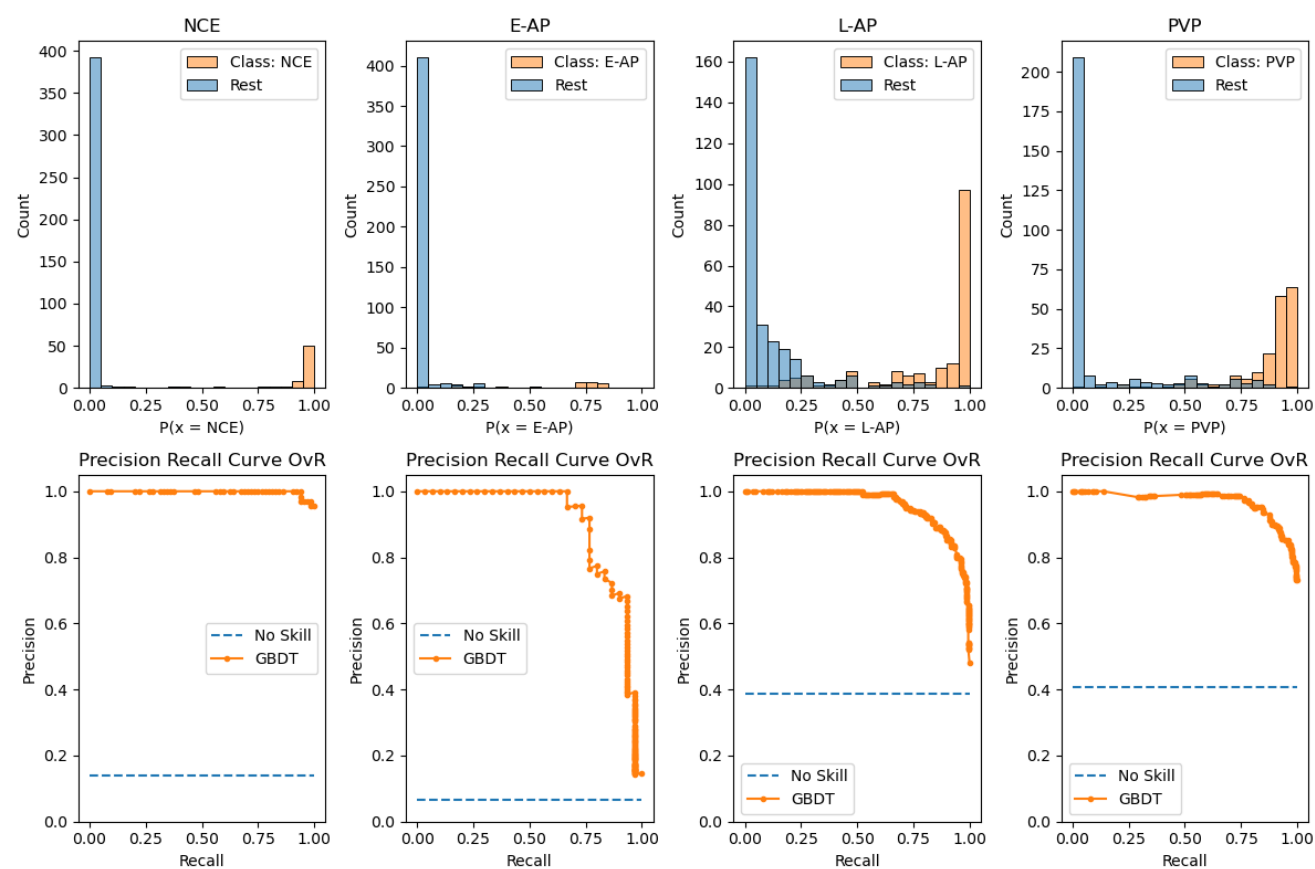

## GBDT OvO

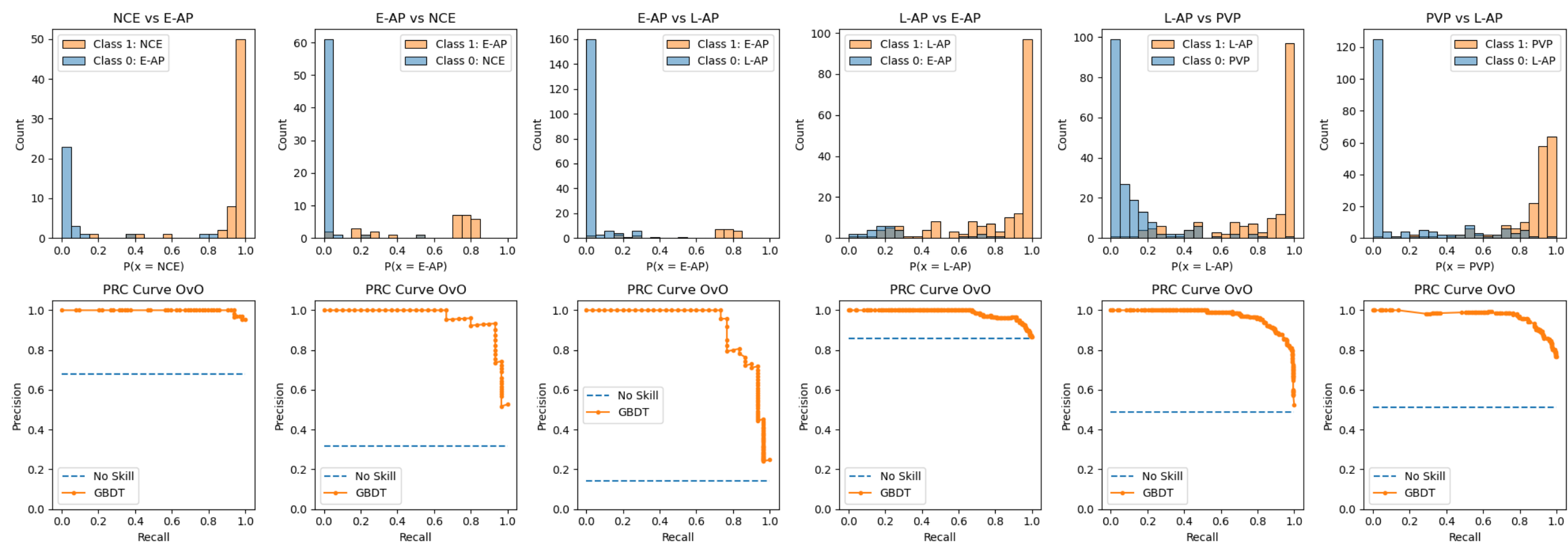

Supplement: S4 Fig — (A), (B), (C), (D) and (E) display the PRCs for the logistic regression (LR), support vector machine (SVM), decision tree (DT), random forest (RF), and gradient-boosted decision tree (GBDT) models, respectively. For each model, the graphs evaluated using a One vs. Rest (OvR) approach are shown on the top and a One vs. One (OvO) approach are shown on the bottom (note that only the OvO PRCs for consecutive phases are shown). See S2 Fig for more details on their interpretation. (PDF) [file pone.0294581.s004.pdf]

A) LR

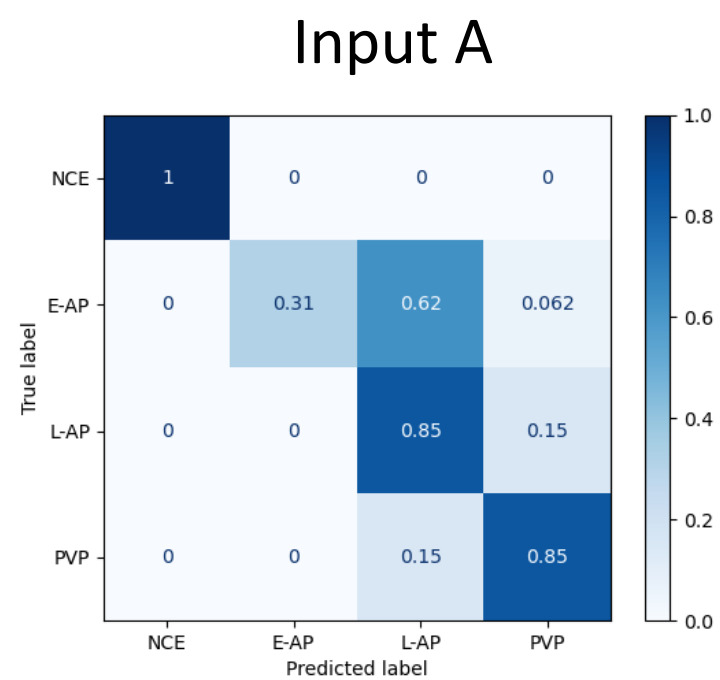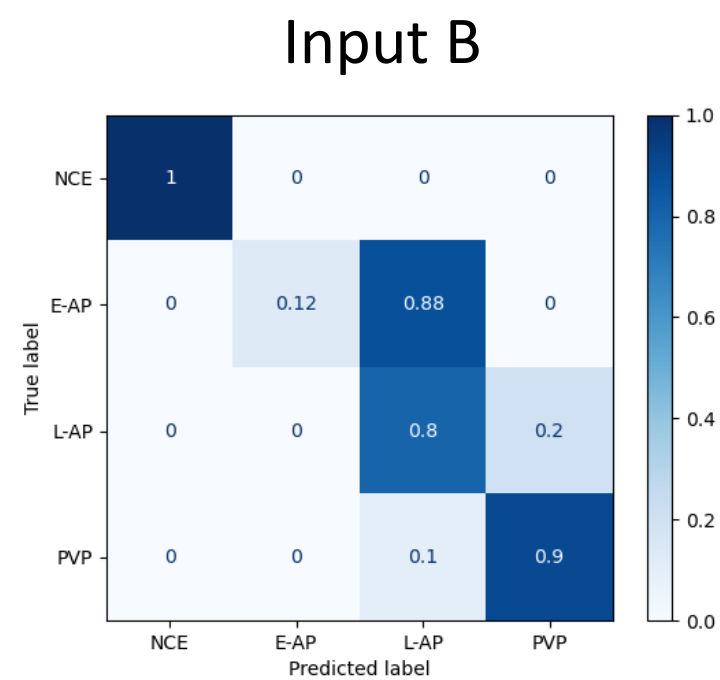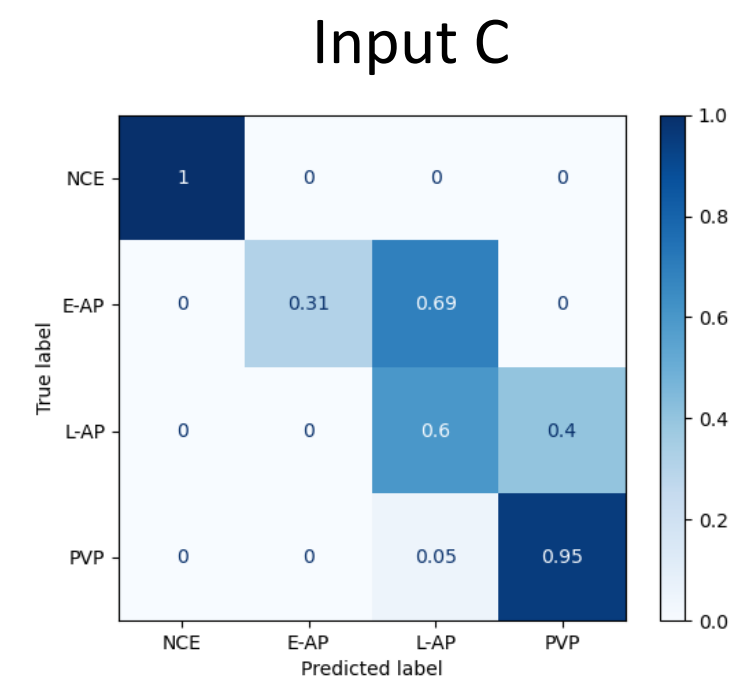

B) SVM

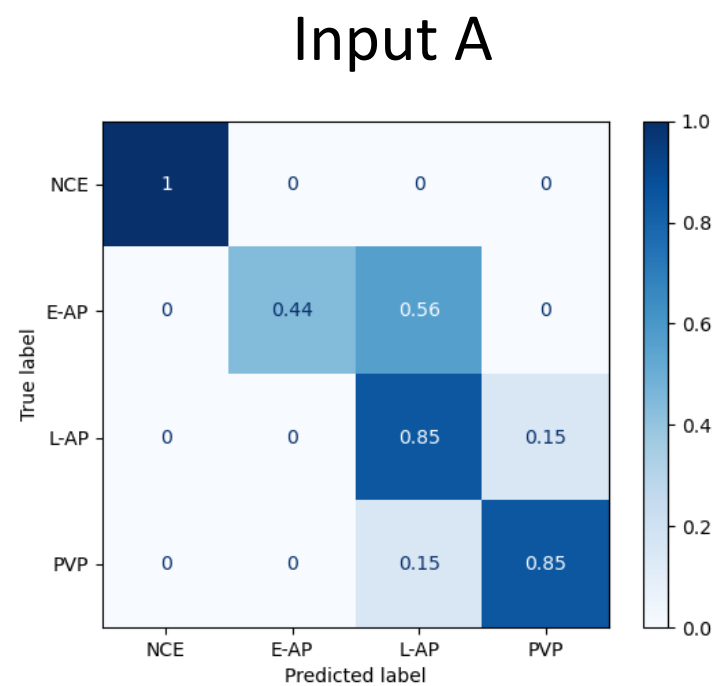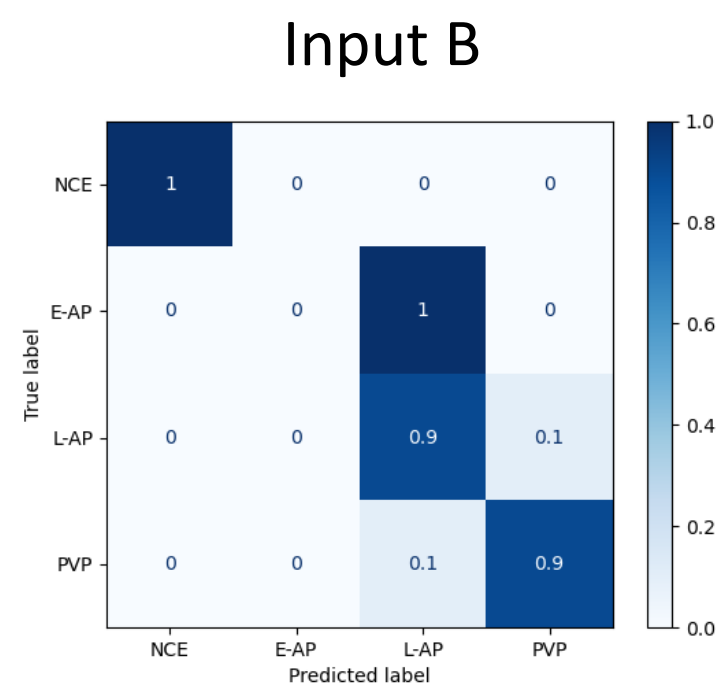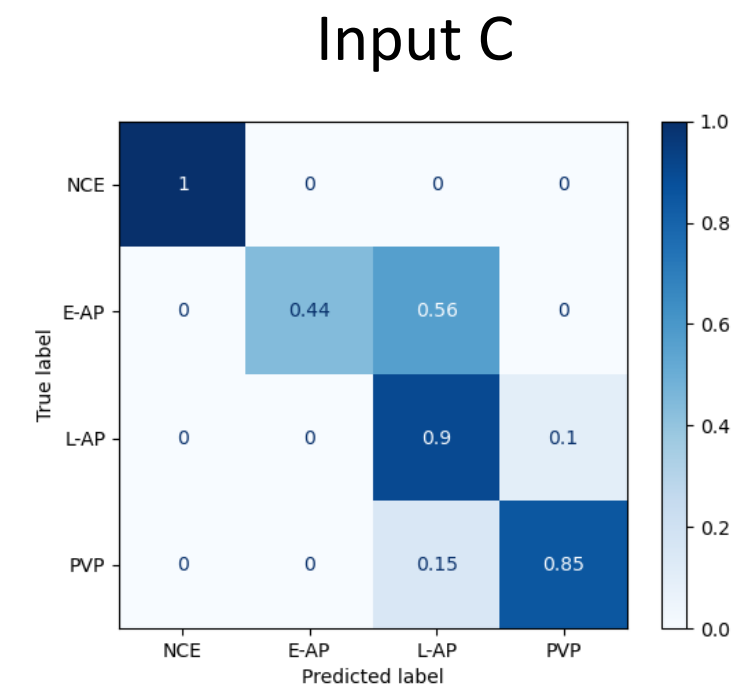

C) DT

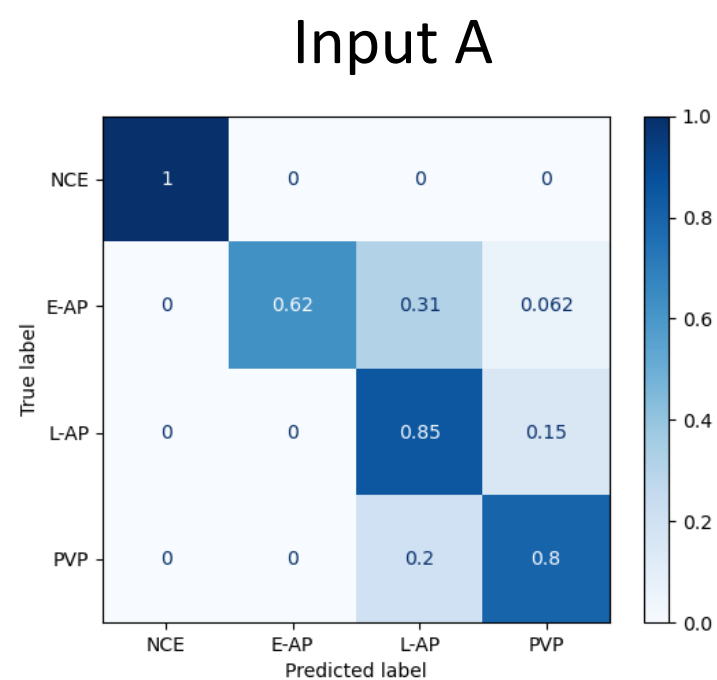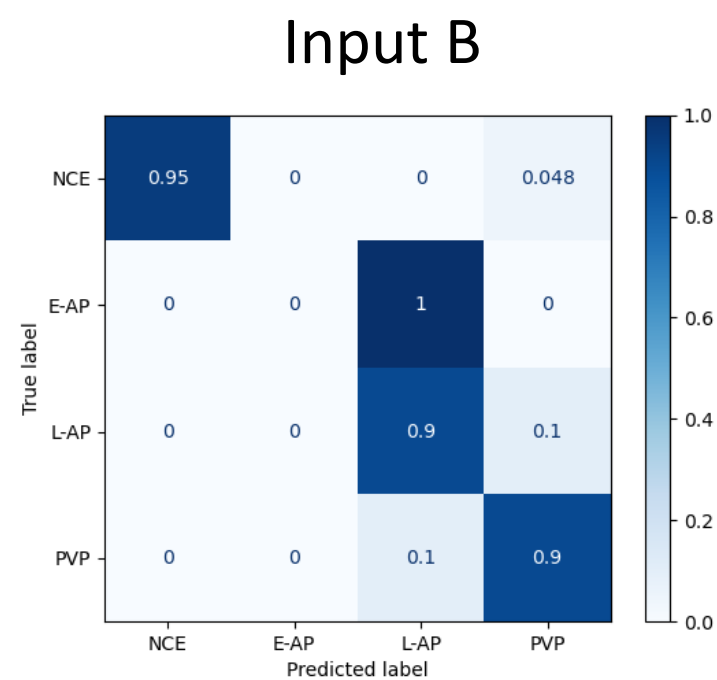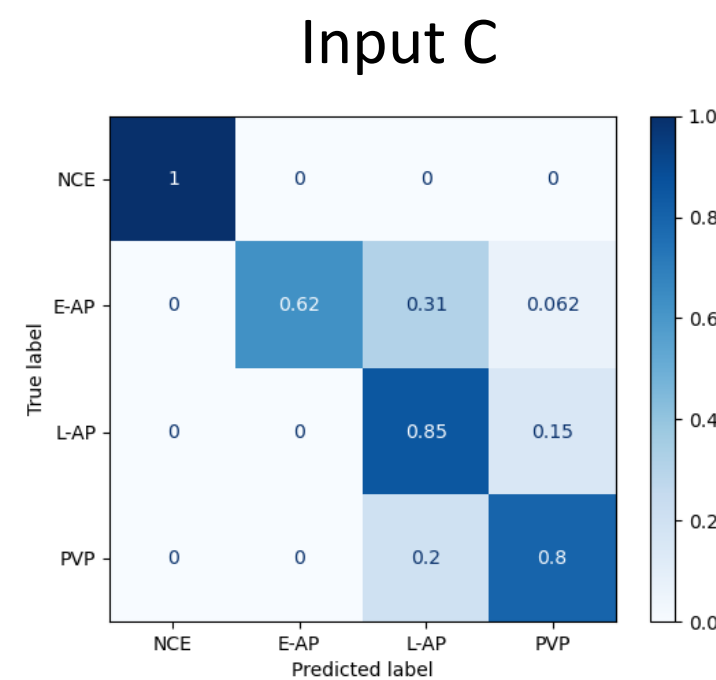

D) RF

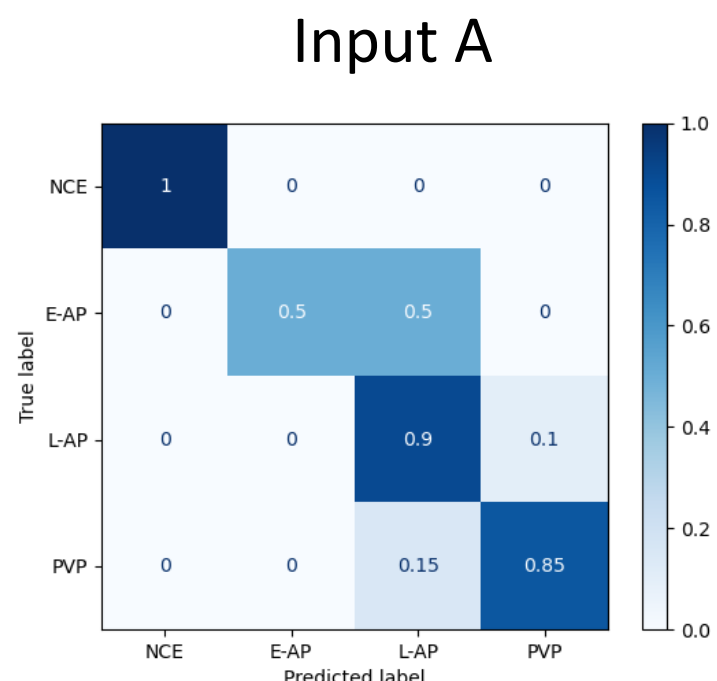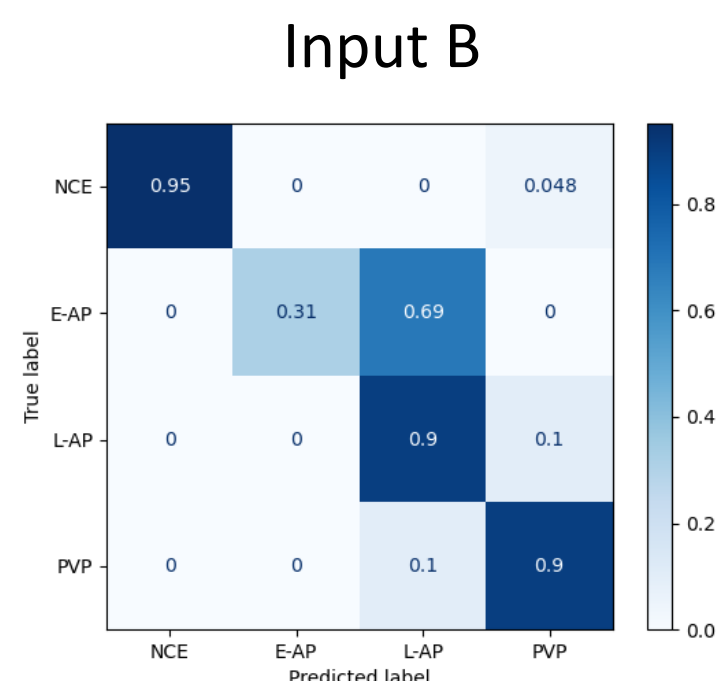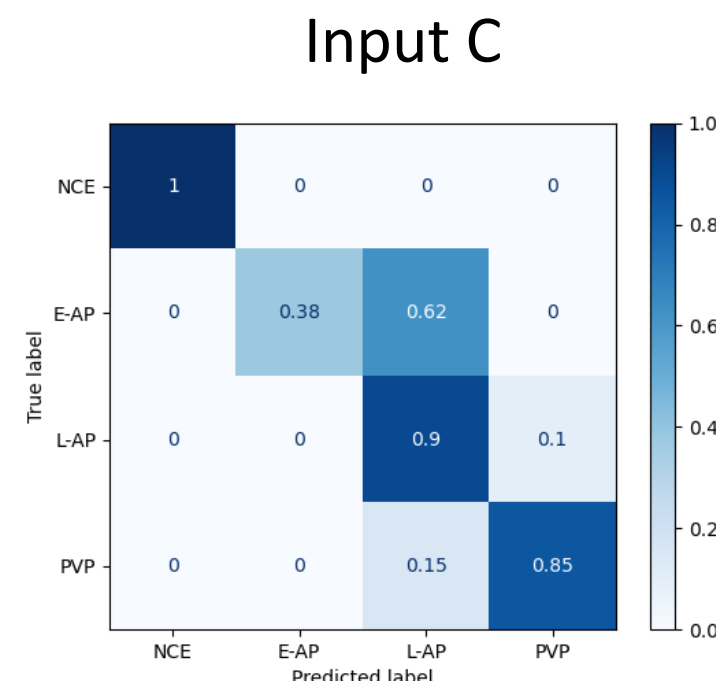

E) GBDT

Input A

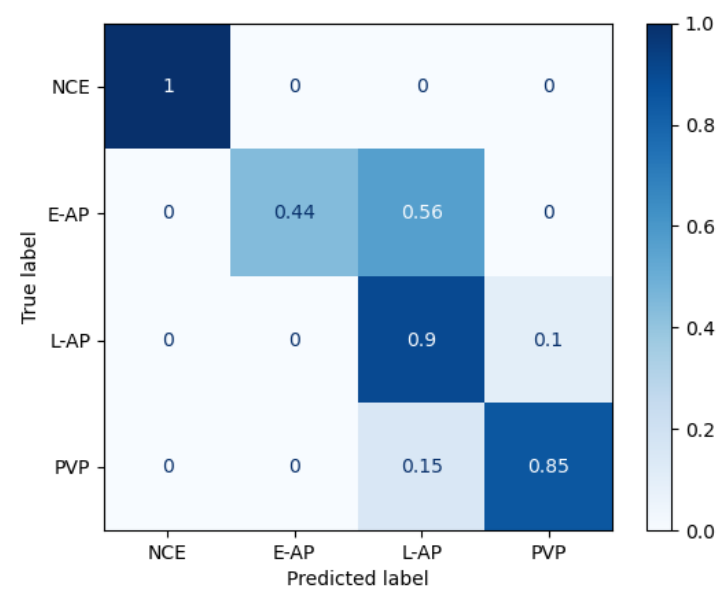

Input B

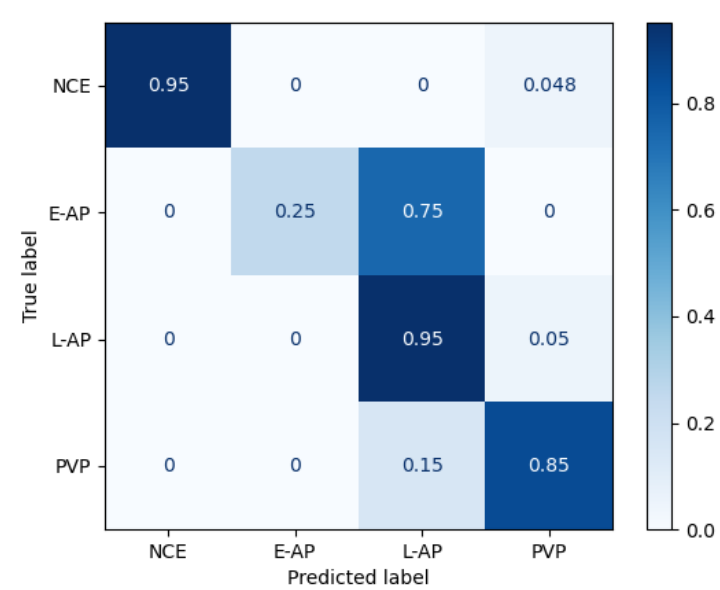

Input C

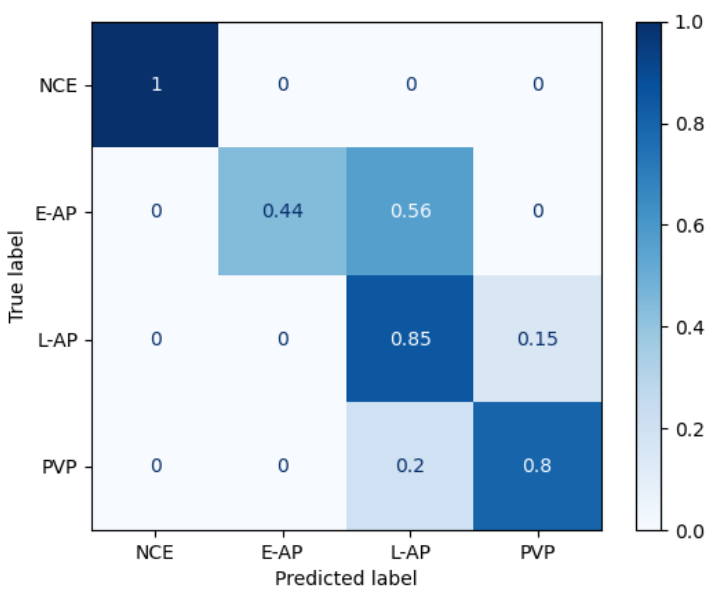

Supplement: S5 Fig — (A), (B), (C), (D) and (E) display the normalized confusion matrices for the logistic regression (LR), support vector machine (SVM), decision tree (DT), random forest (RF), and gradient-boosted decision tree (GBDT) models, respectively. Each matrix shows the performance on the same selected training-testing pair for models trained with Inputs A, B, and C from left to right. The models faced greater difficulty than in Cohort A in distinguishing early arterial phase (E-AP) from late arterial phase (L-AP), especially when trained with Input B. (PDF) [file pone.0294581.s005.pdf]

# A) LR OvR

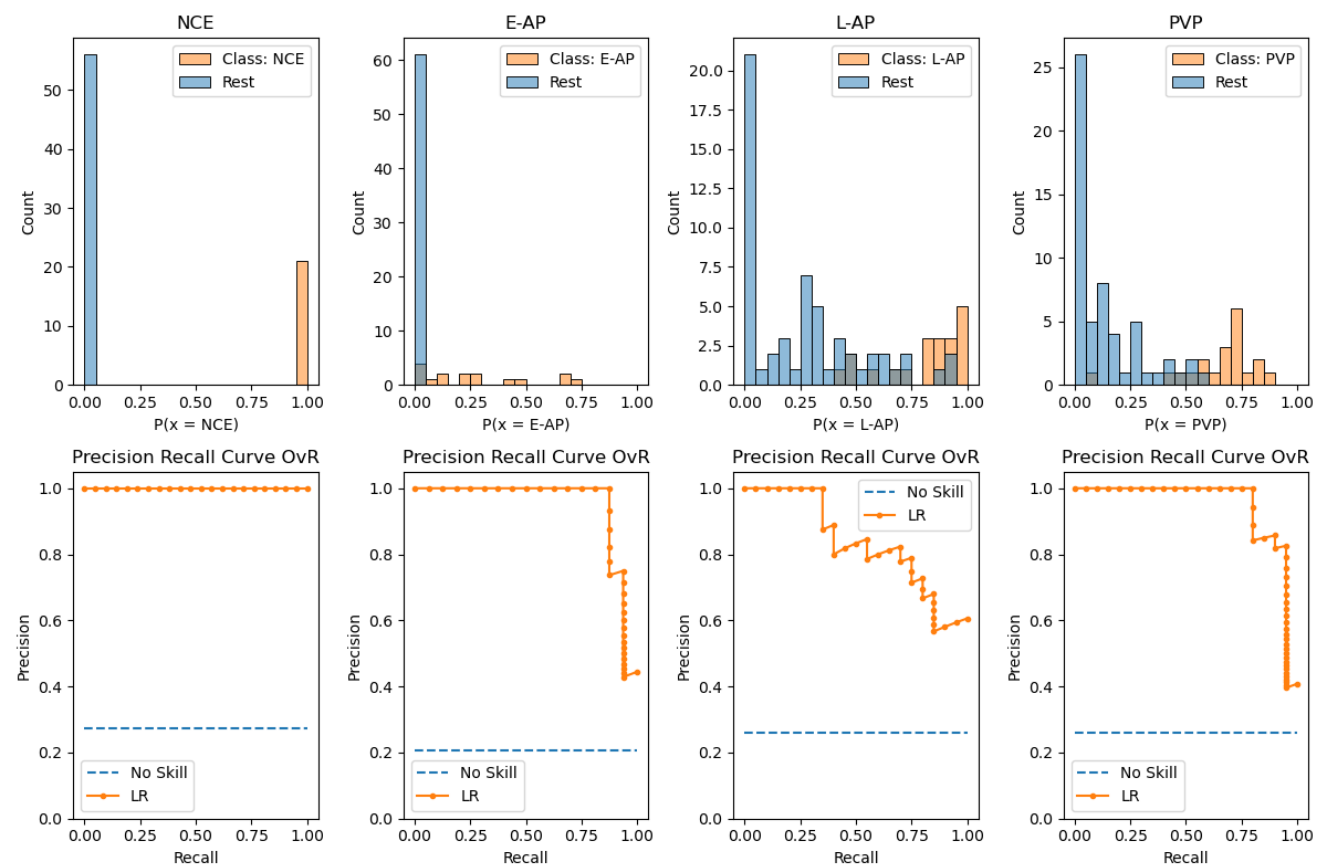

# LR OvO

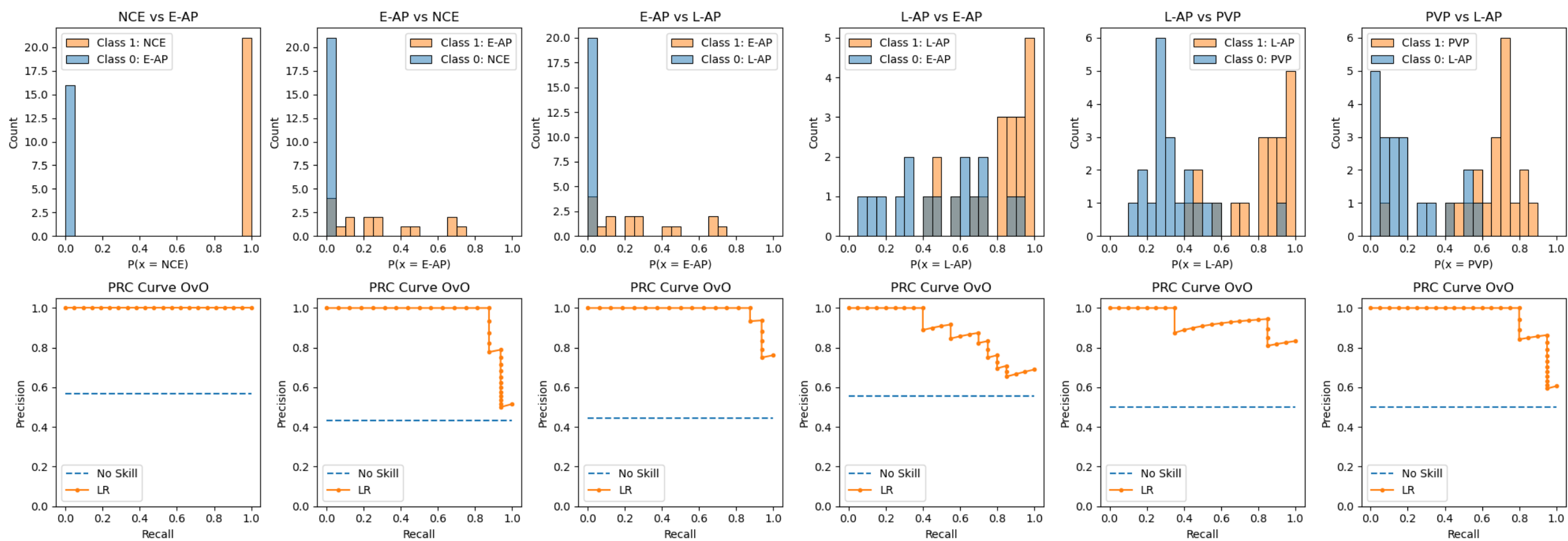

B) SVM OvR

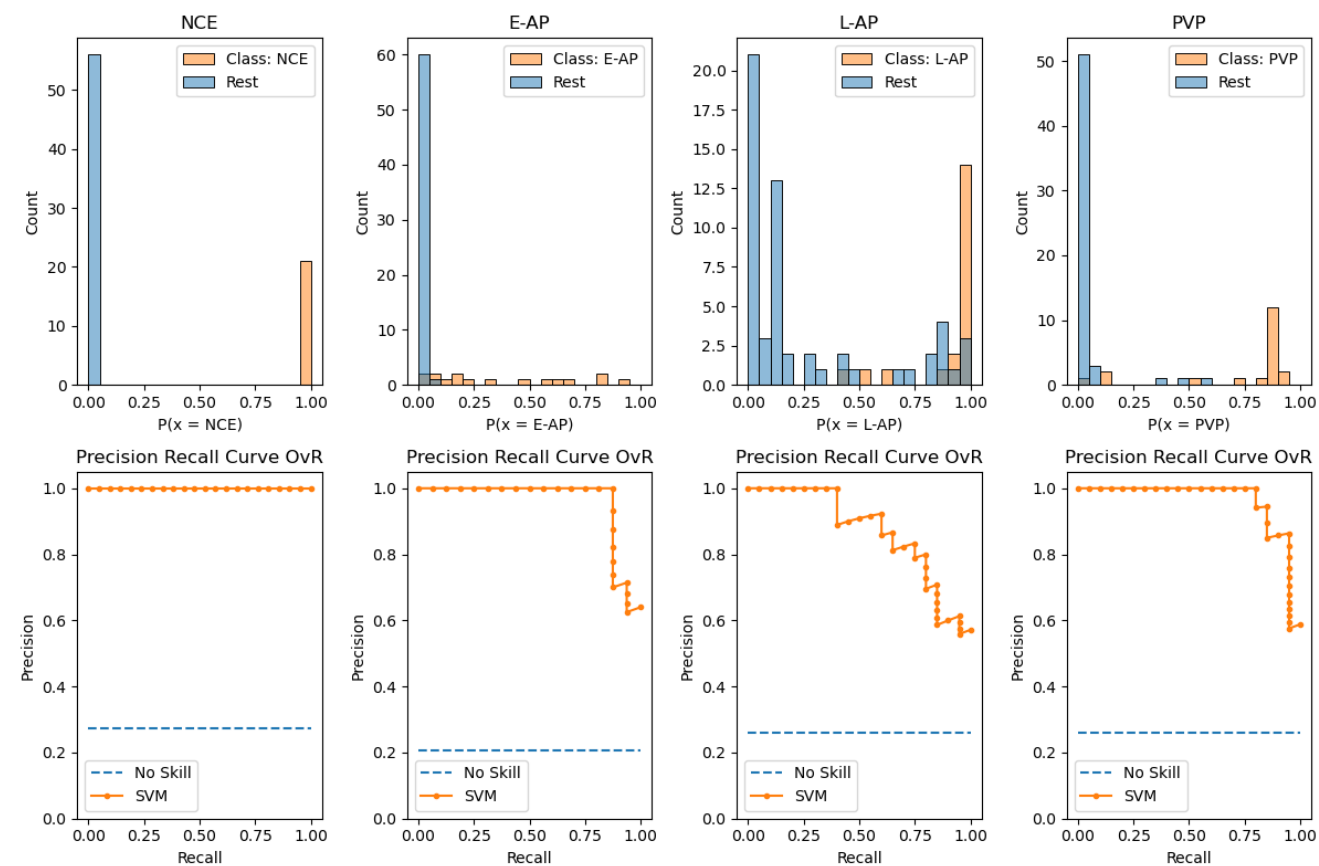

SVM OvO

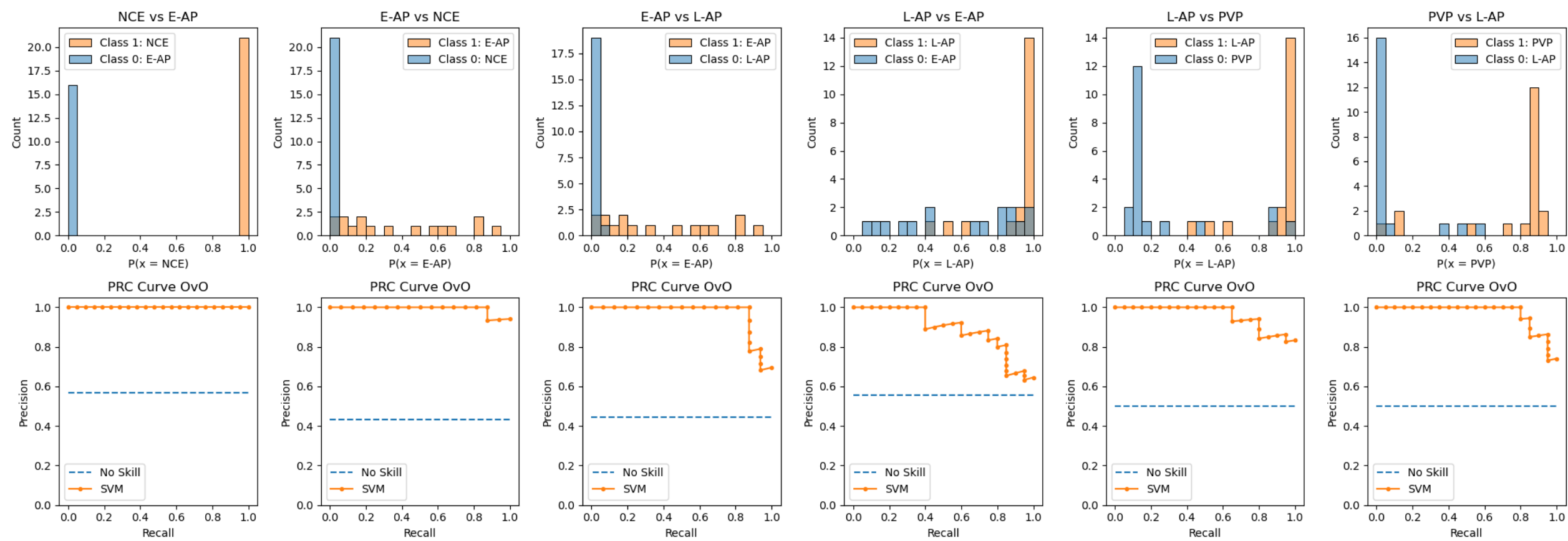

# C) DT OvR

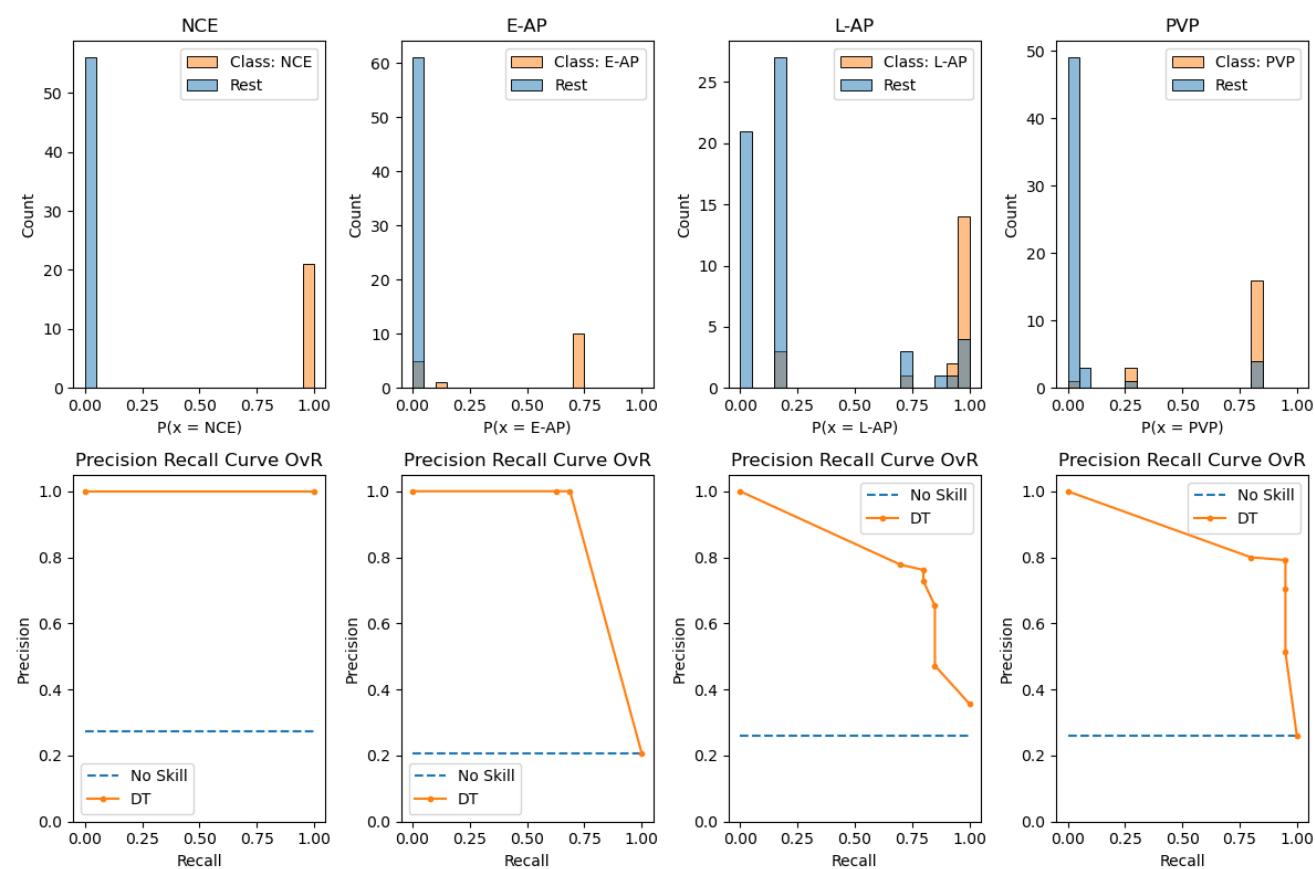

# DT OvO

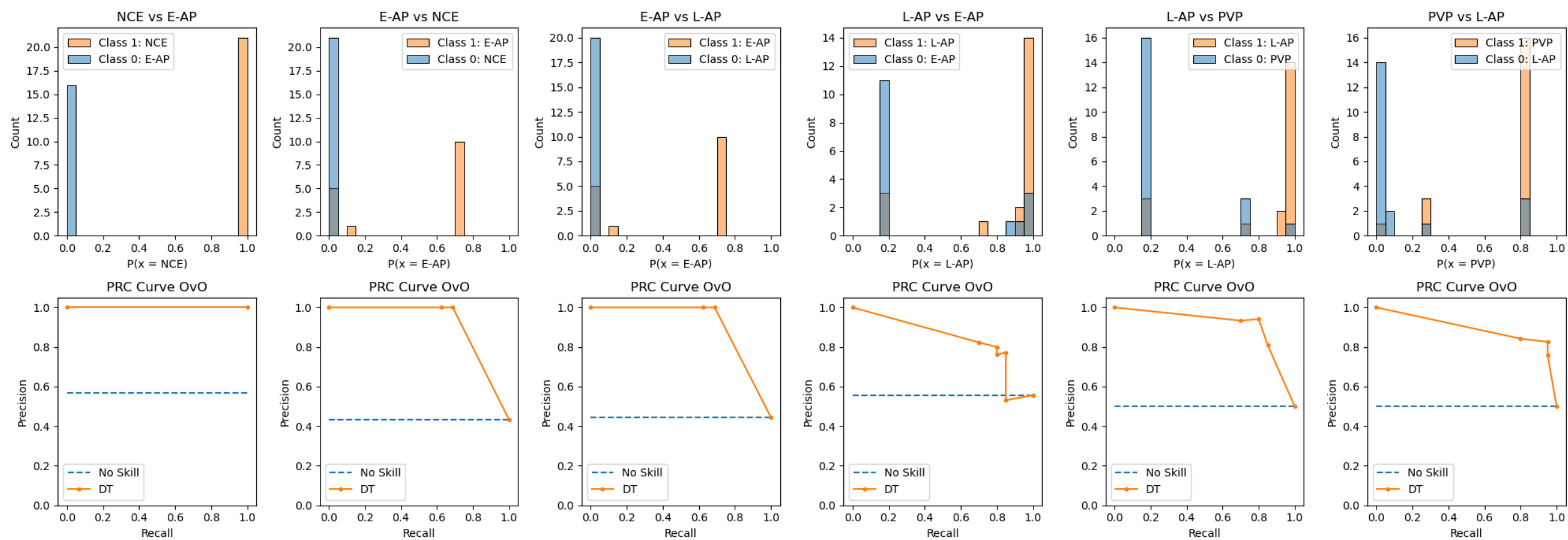

D) RF OvR

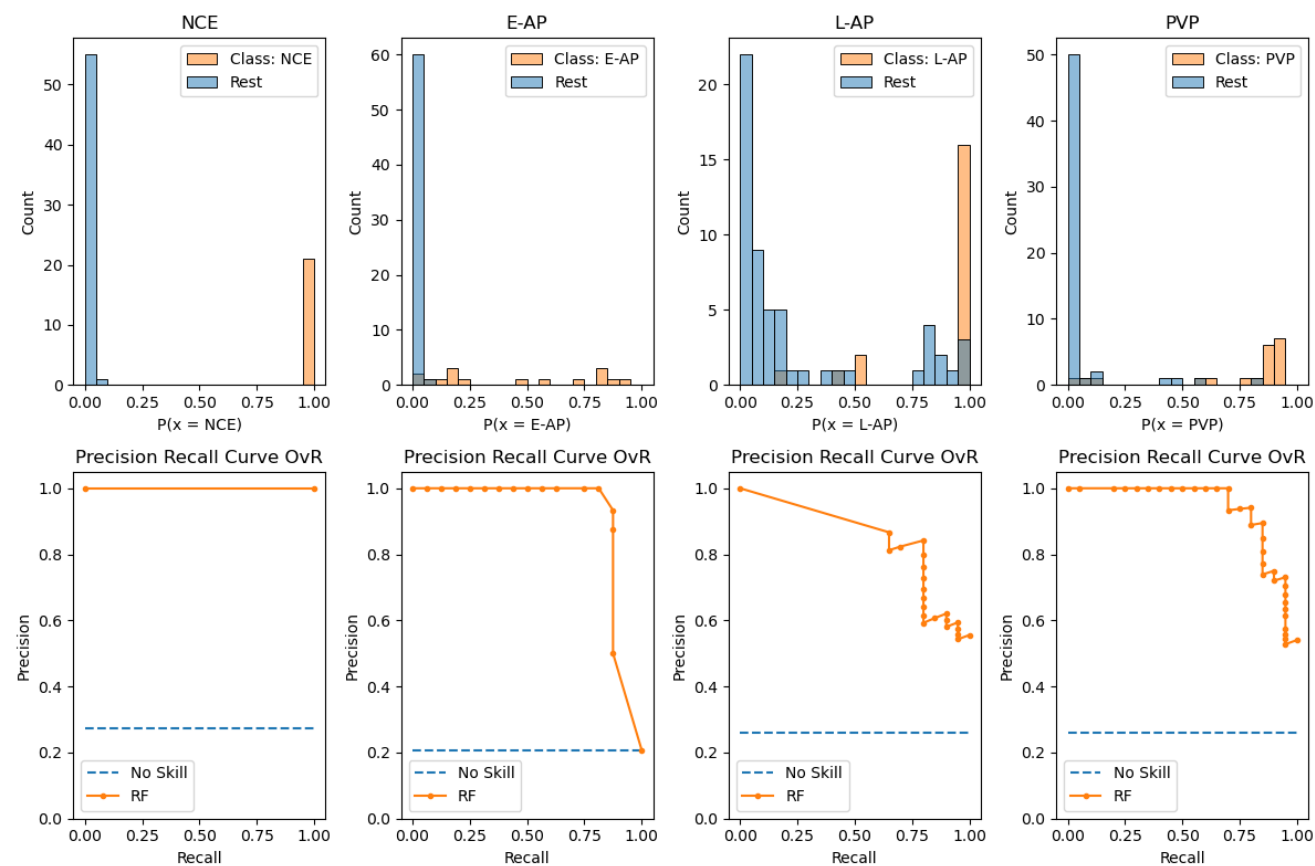

RF OvO

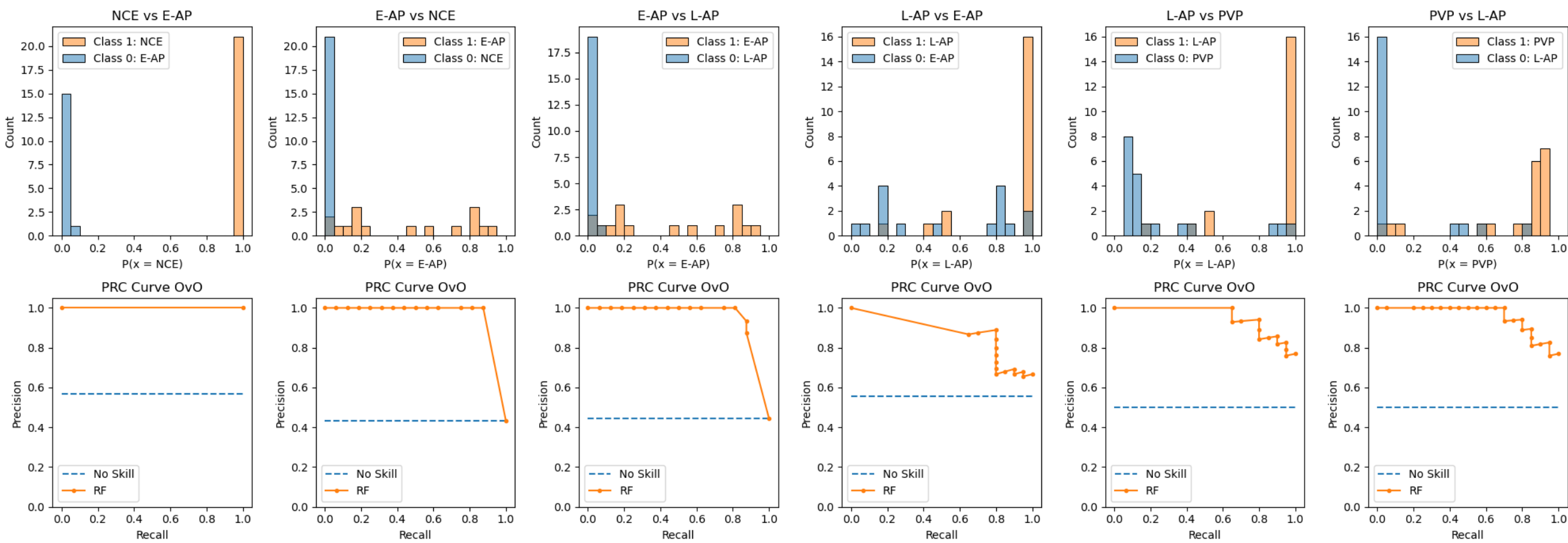

E) GBDT OvR

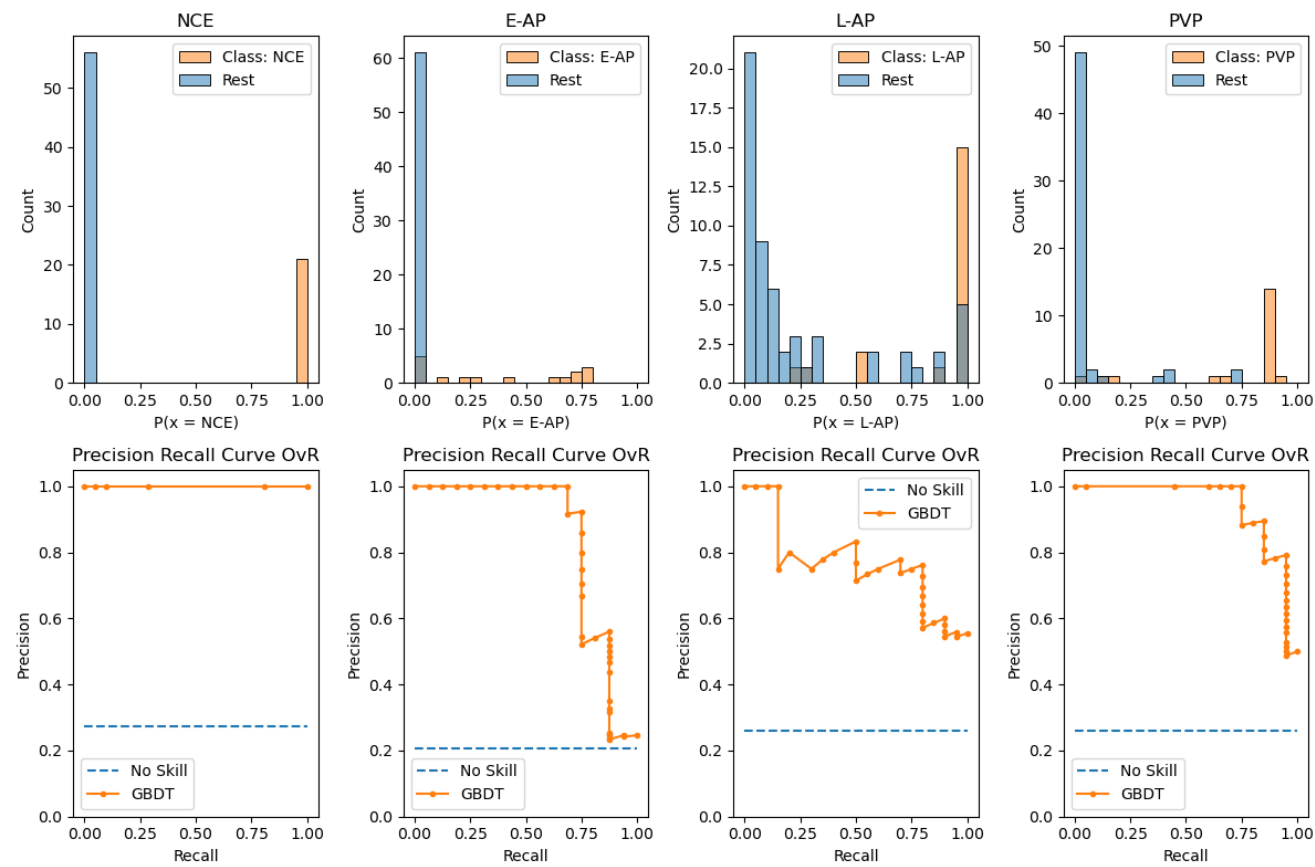

GBDT OvO

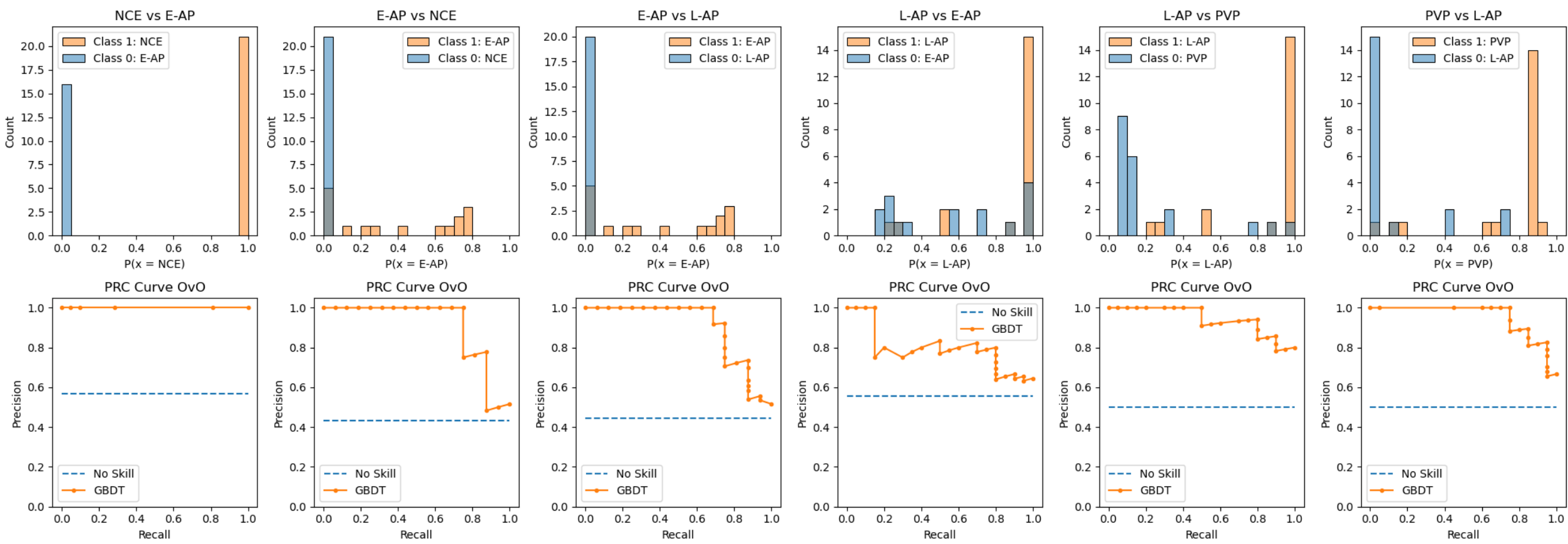

Supplement: S6 Fig — (A), (B), (C), (D) and (E) display the PRCs for the logistic regression (LR), support vector machine (SVM), decision tree (DT), random forest (RF), and gradient-boosted decision tree (GBDT) models, respectively. For each model, the graphs evaluated using a One vs. Rest (OvR) approach are shown on the top and a One vs. One (OvO) approach are shown on the bottom (note that only the OvO PRCs for consecutive phases are shown). See S2 Fig for more details on their interpretation. (PDF) [file pone.0294581.s006.pdf]

# A) LR OvR

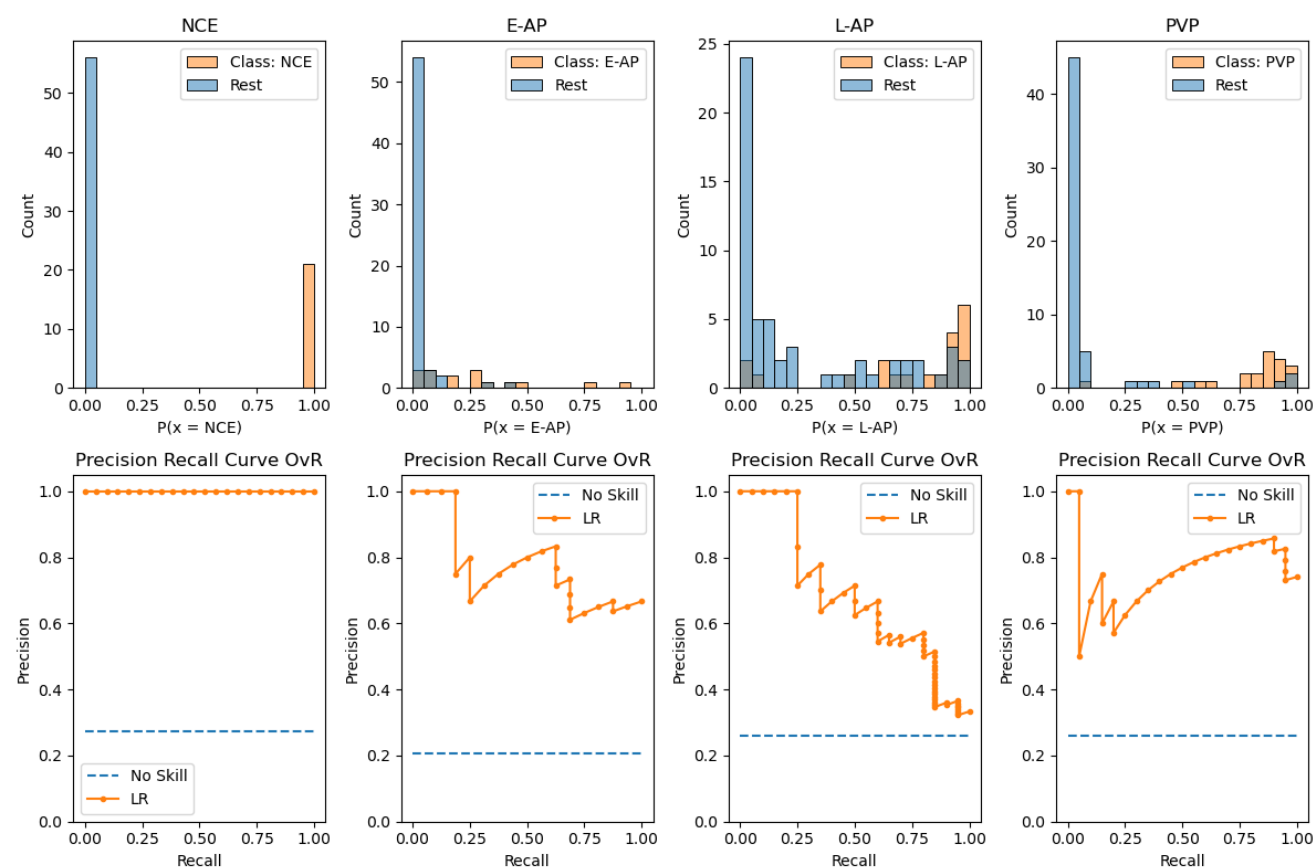

# LR OvO

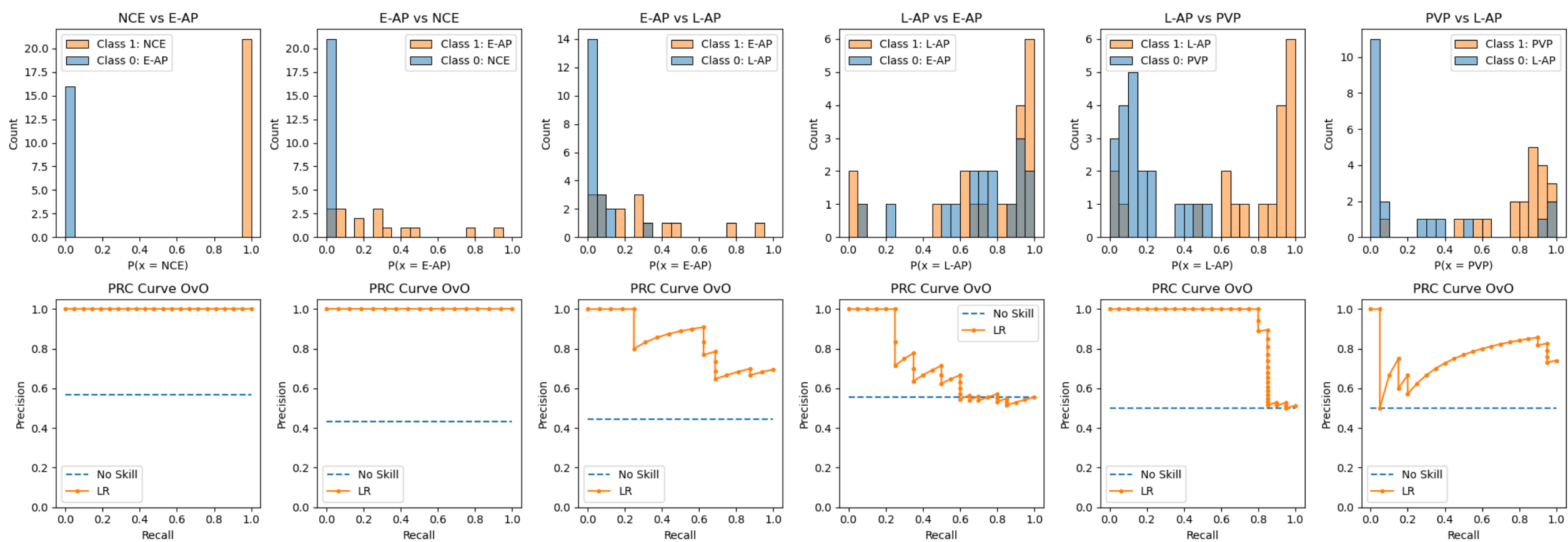

## B) SVM OvR

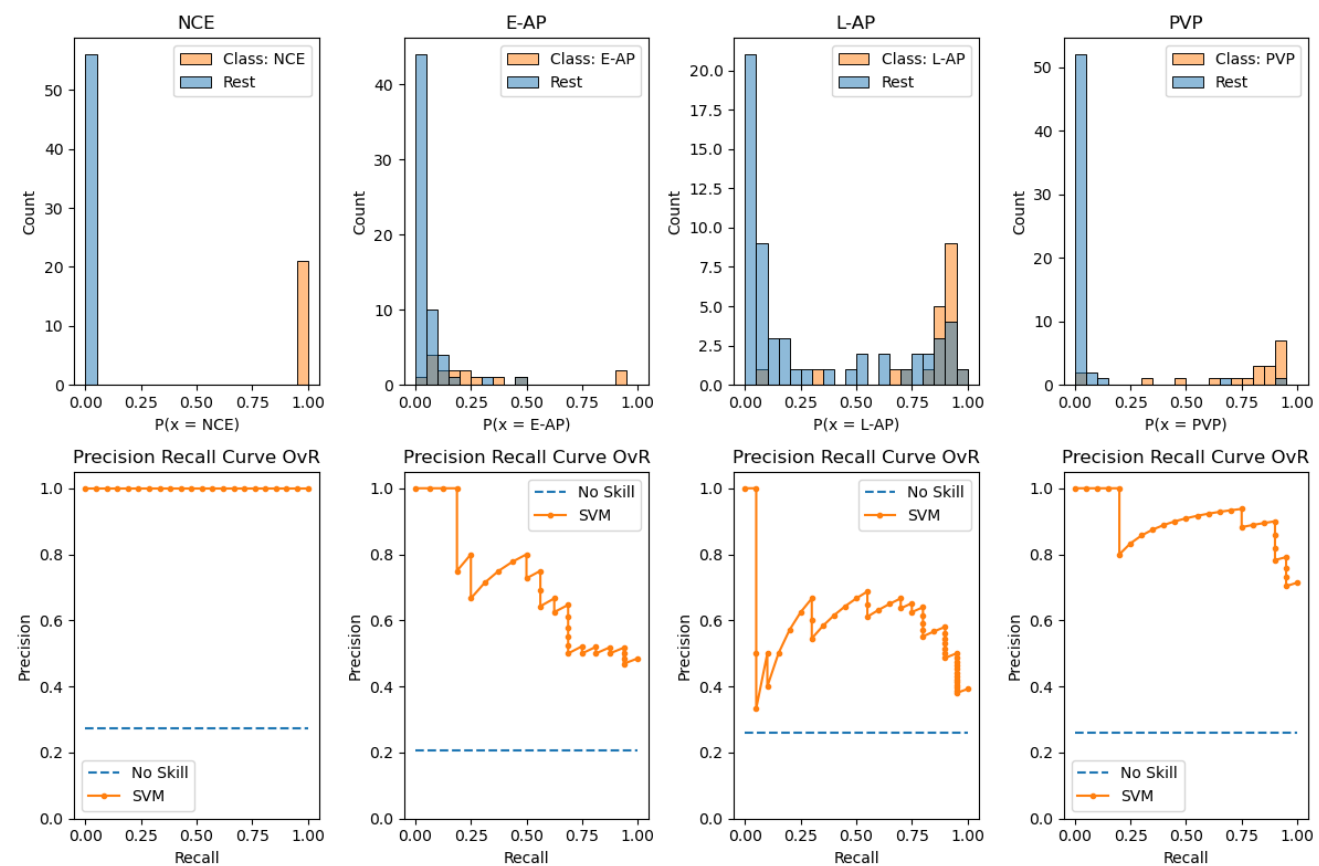

## SVM OvO

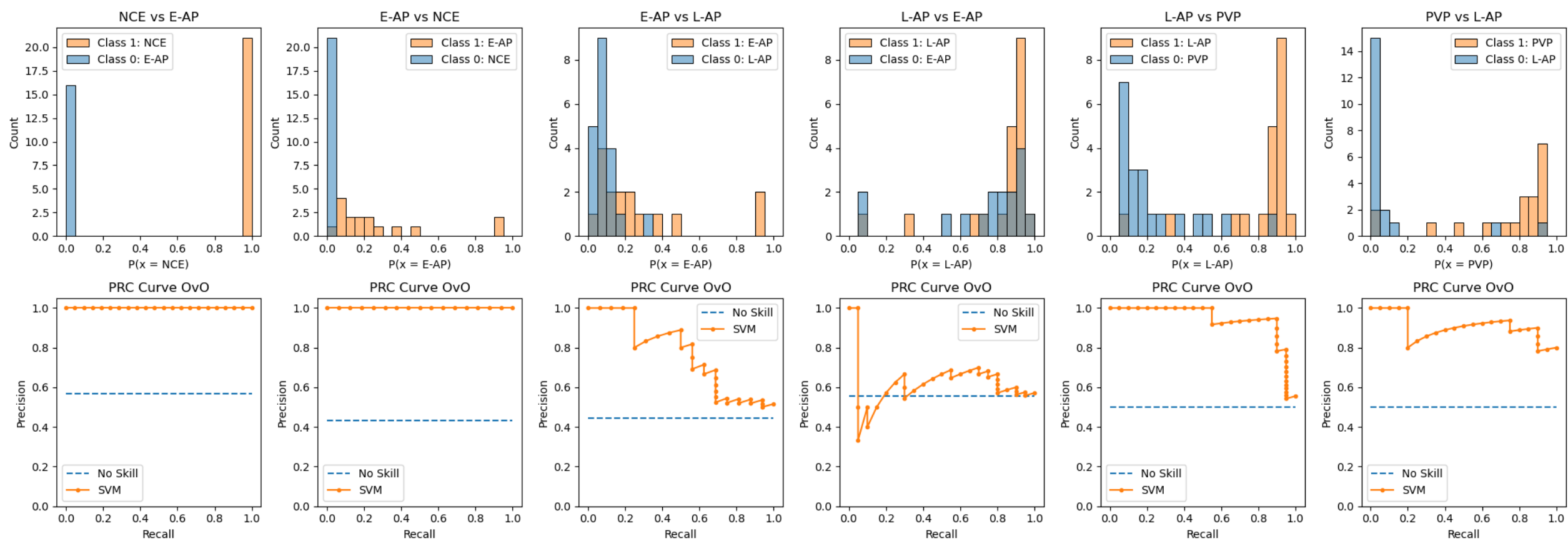

# C) DT OvR

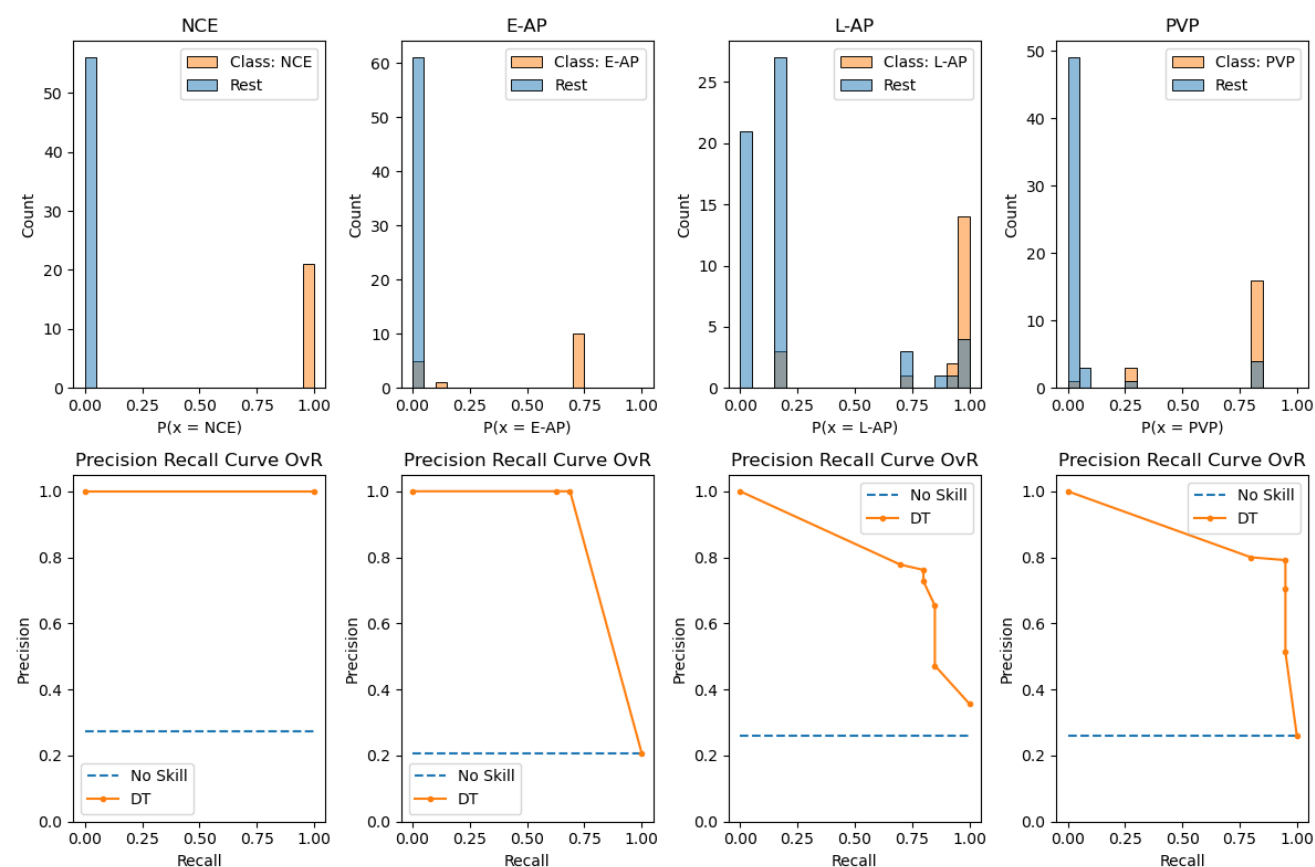

# DT OvO

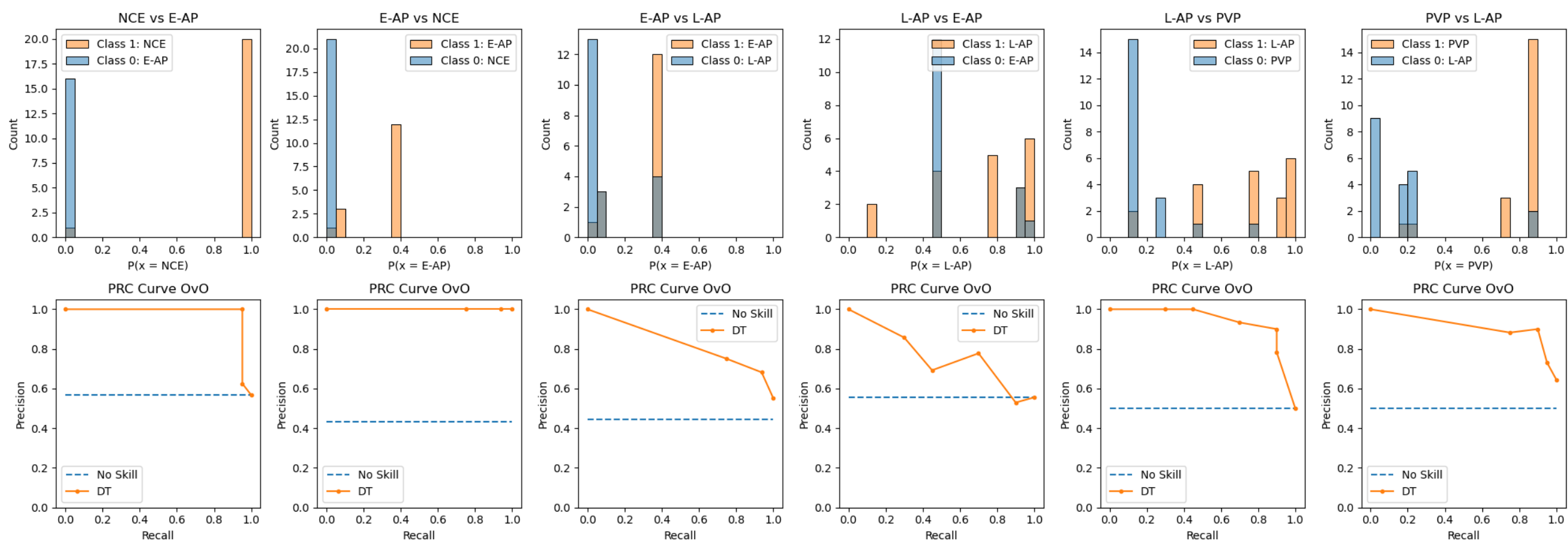

## D) RF OvR

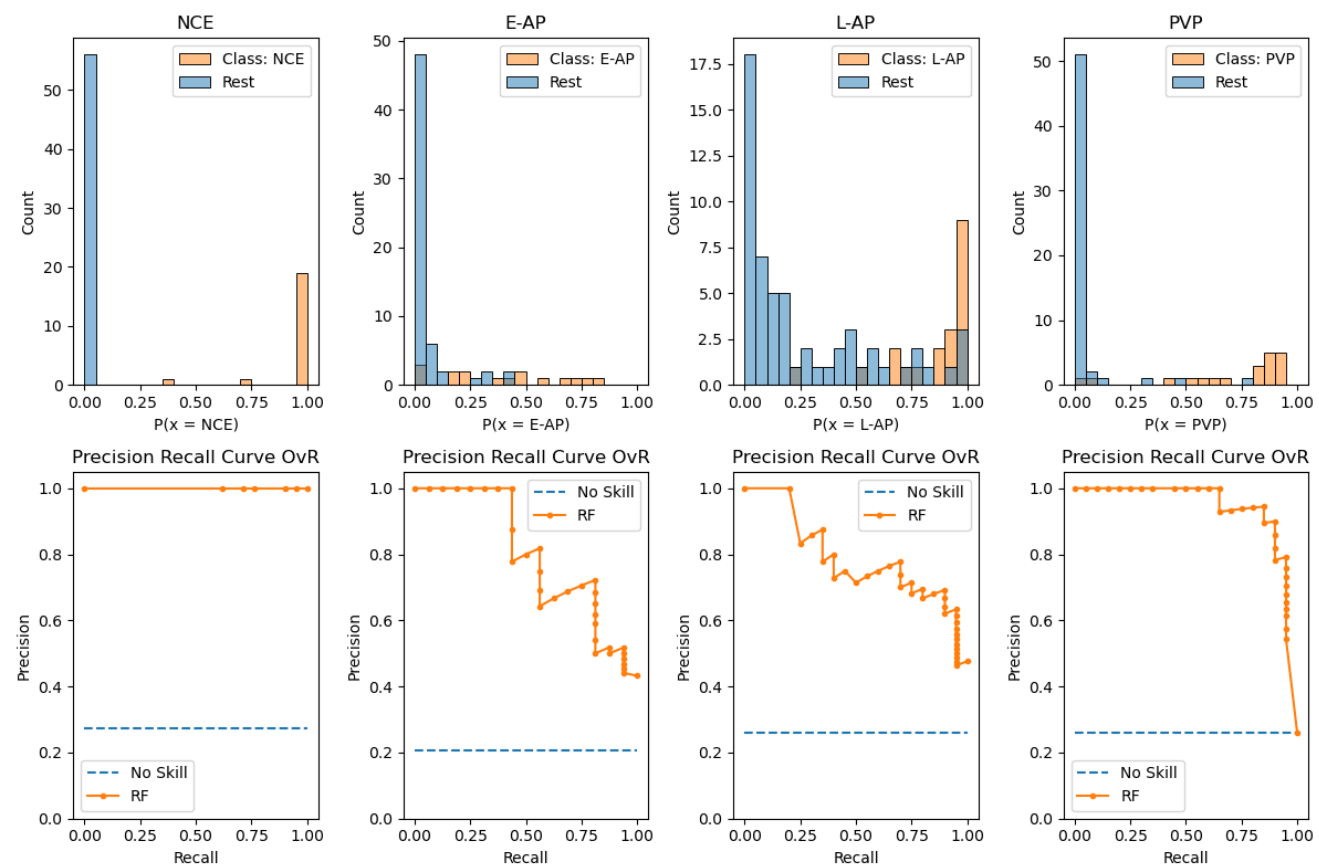

## RF OvO

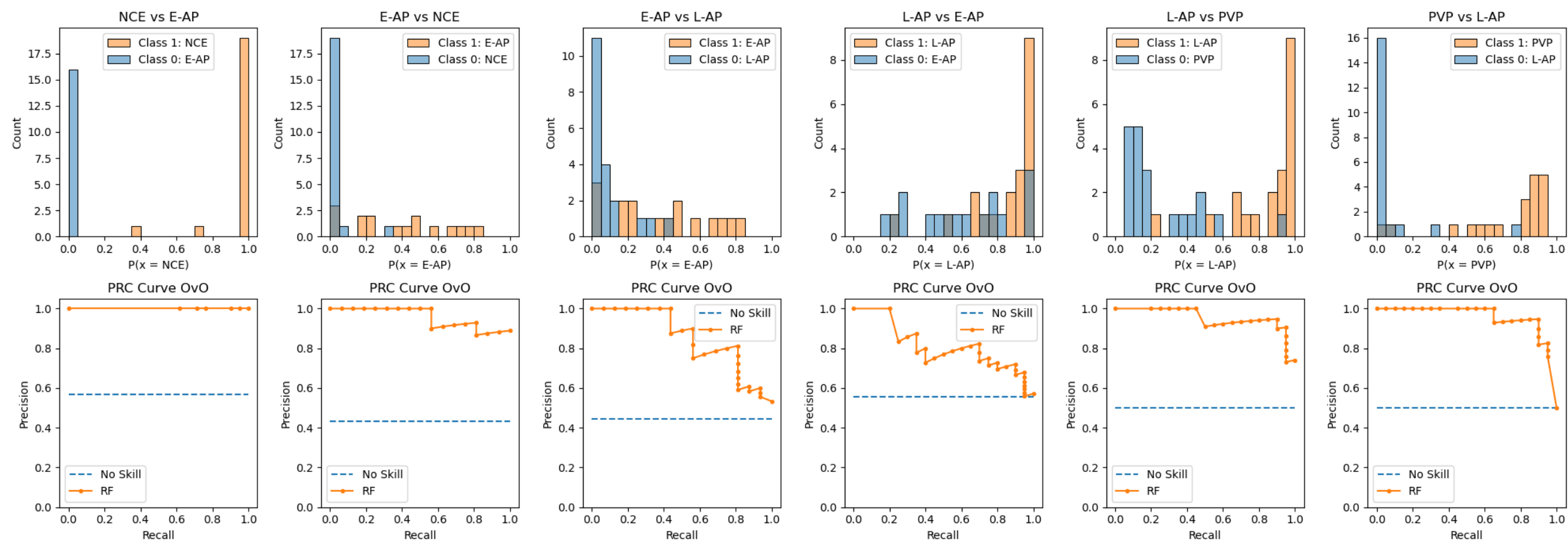

## E) GBDT OvR

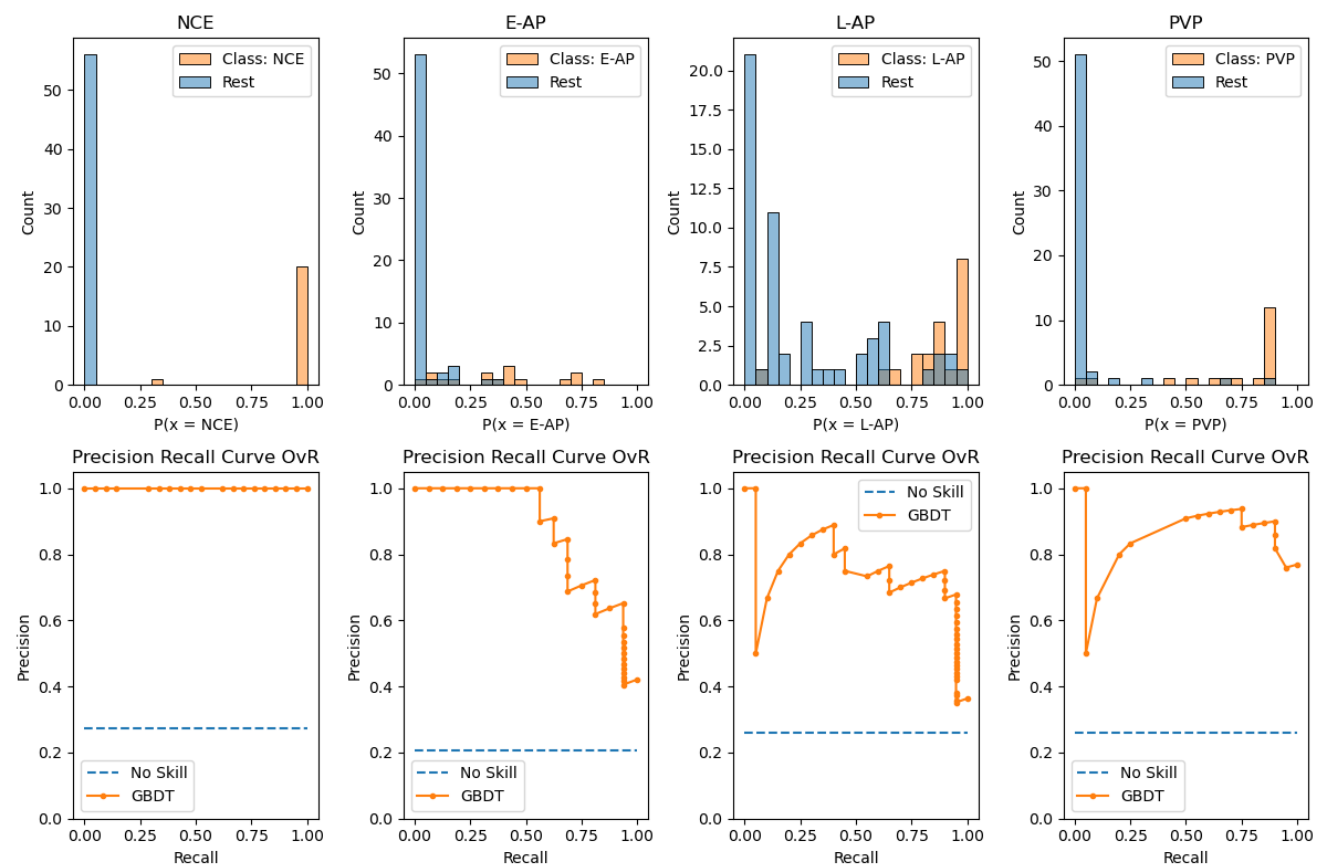

## GBDT OvO

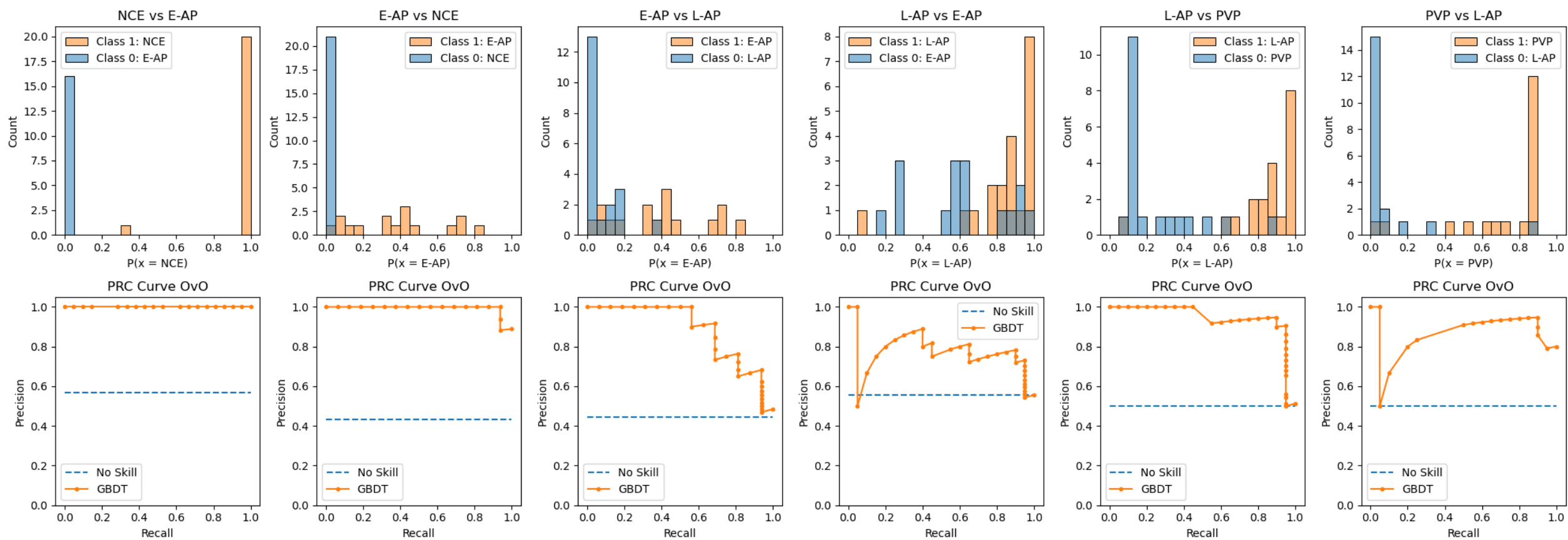

Supplement: S7 Fig — (A), (B), (C), (D) and (E) display the PRCs for the logistic regression (LR), support vector machine (SVM), decision tree (DT), random forest (RF), and gradient-boosted decision tree (GBDT) models, respectively. For each model, the graphs evaluated using a One vs. Rest (OvR) approach are shown on the top and a One vs. One (OvO) approach are shown on the bottom (note that only the OvO PRCs for consecutive phases are shown). See S2 Fig for more details on their interpretation. (PDF) [file pone.0294581.s007.pdf]

# A) LR OvR

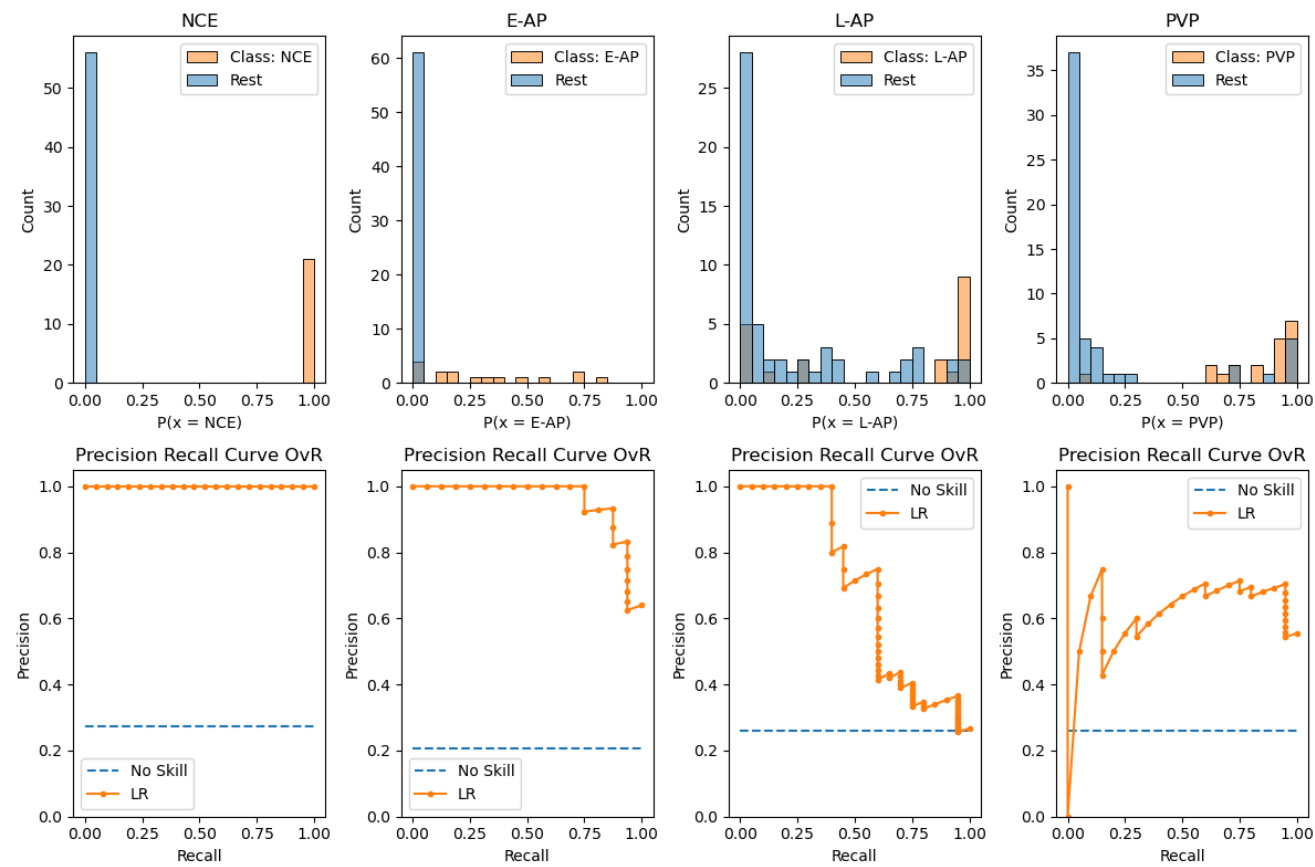

# LR OvO

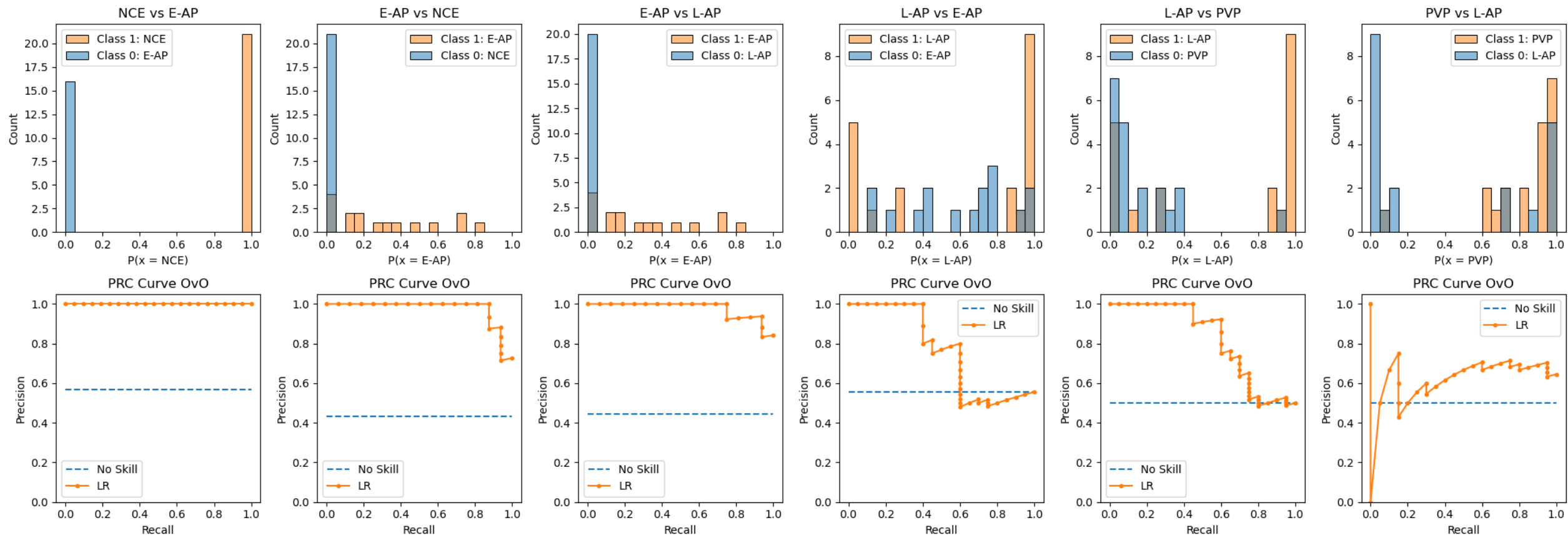

B) SVM OvR

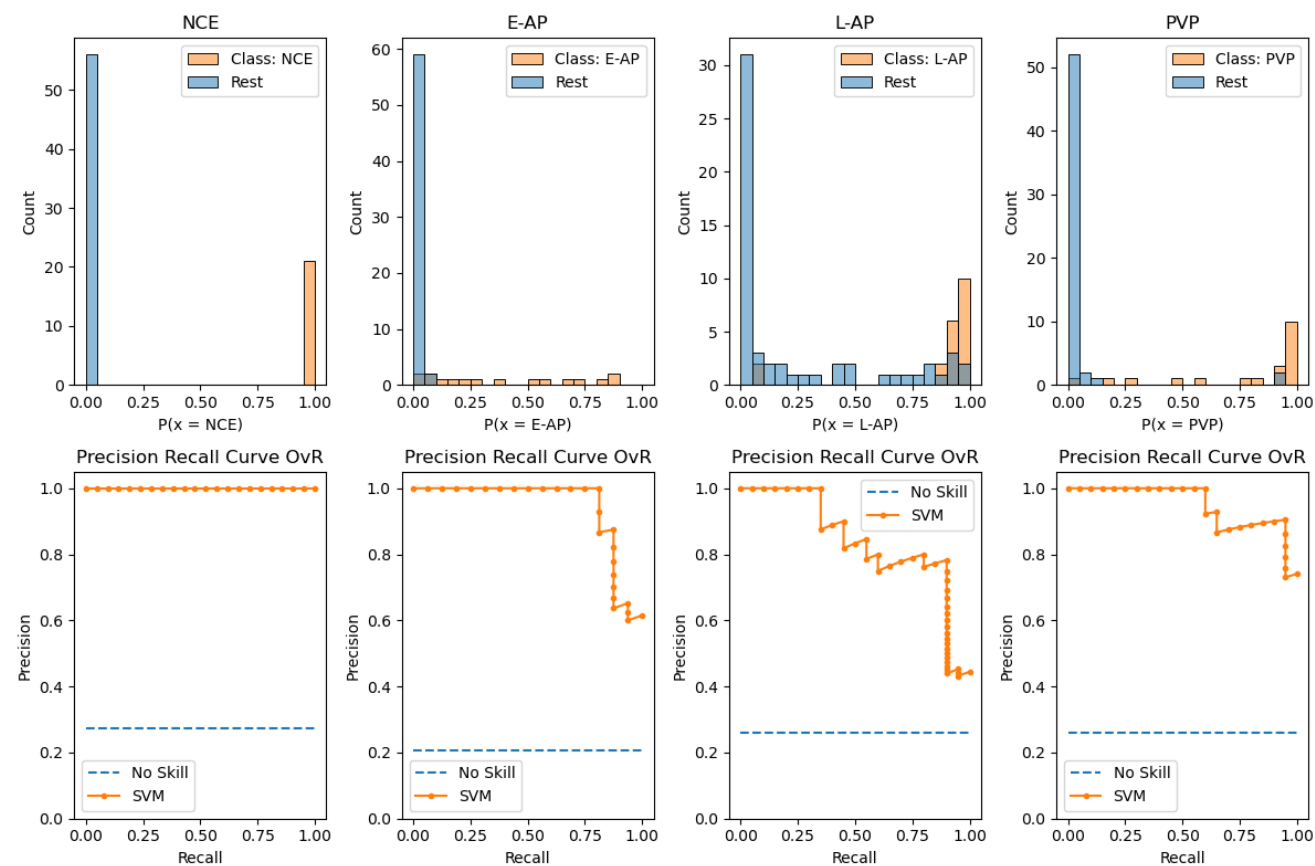

SVM OvO

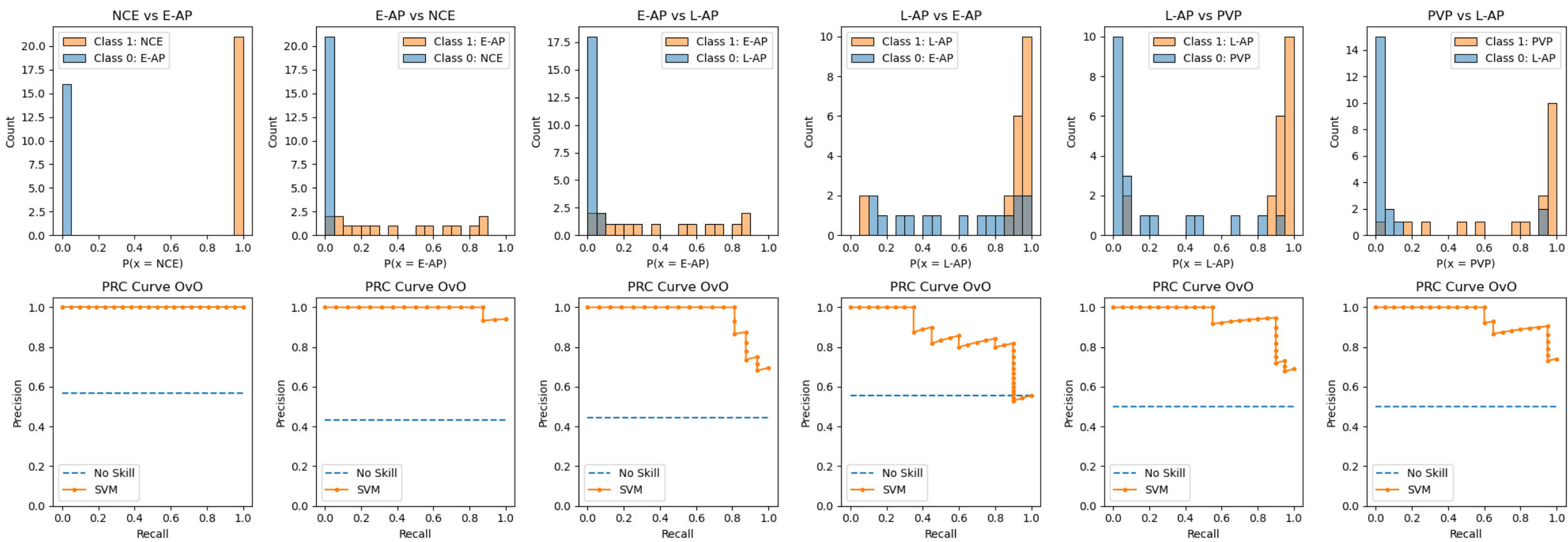

# C) DT OvR

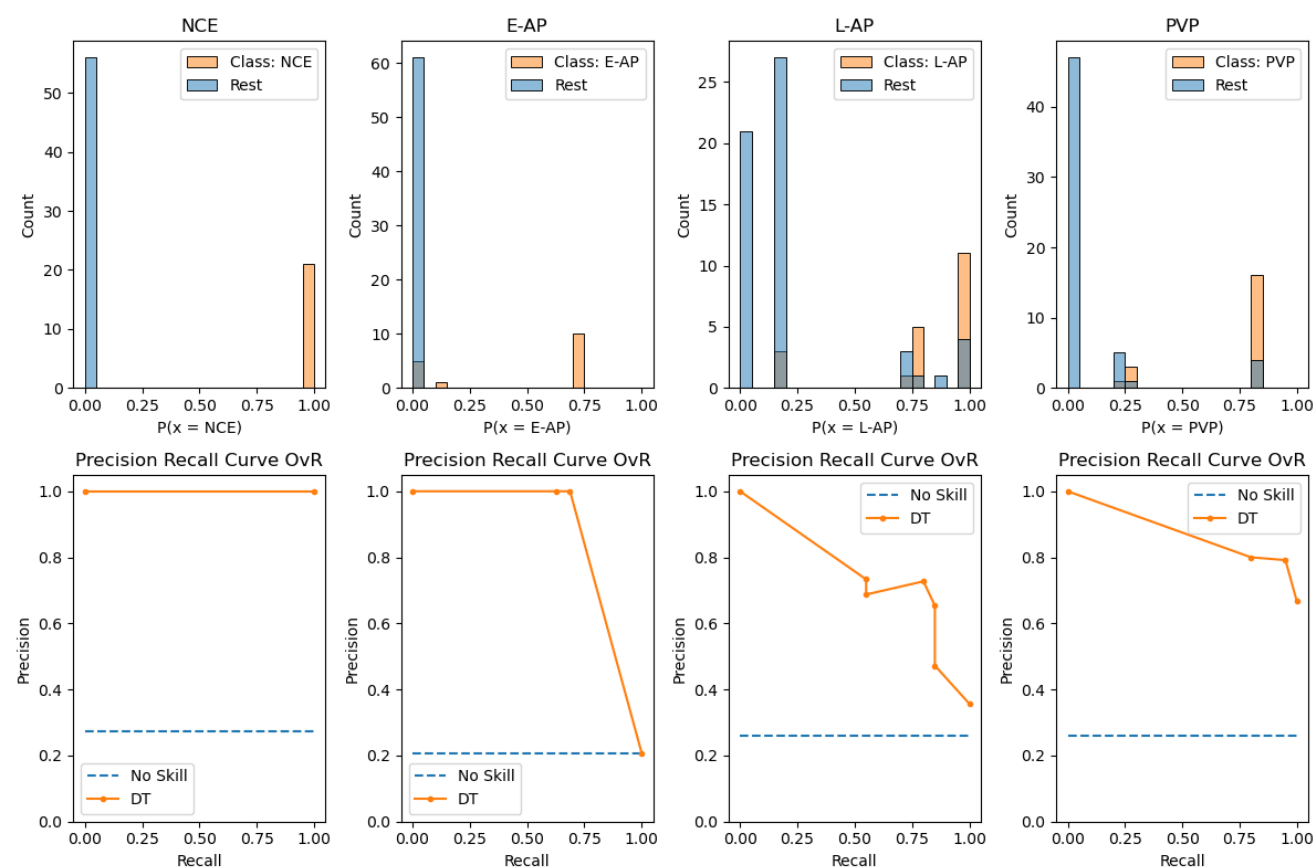

# DT OvO

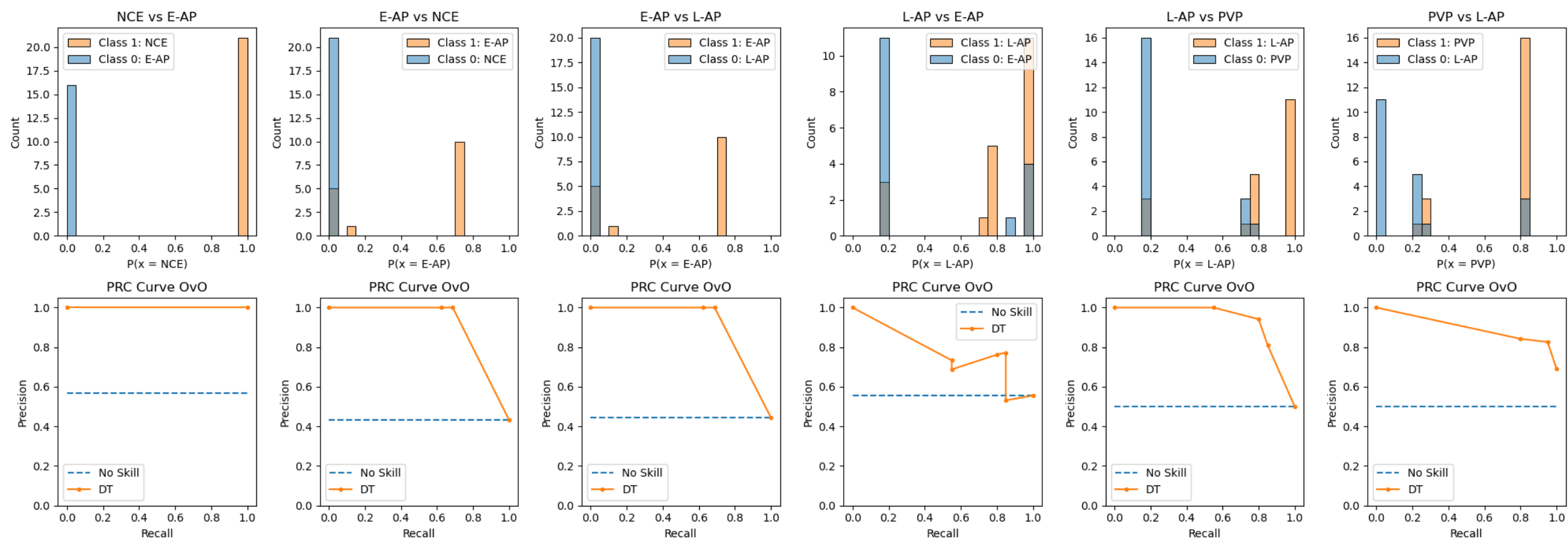

## D) RF OvR

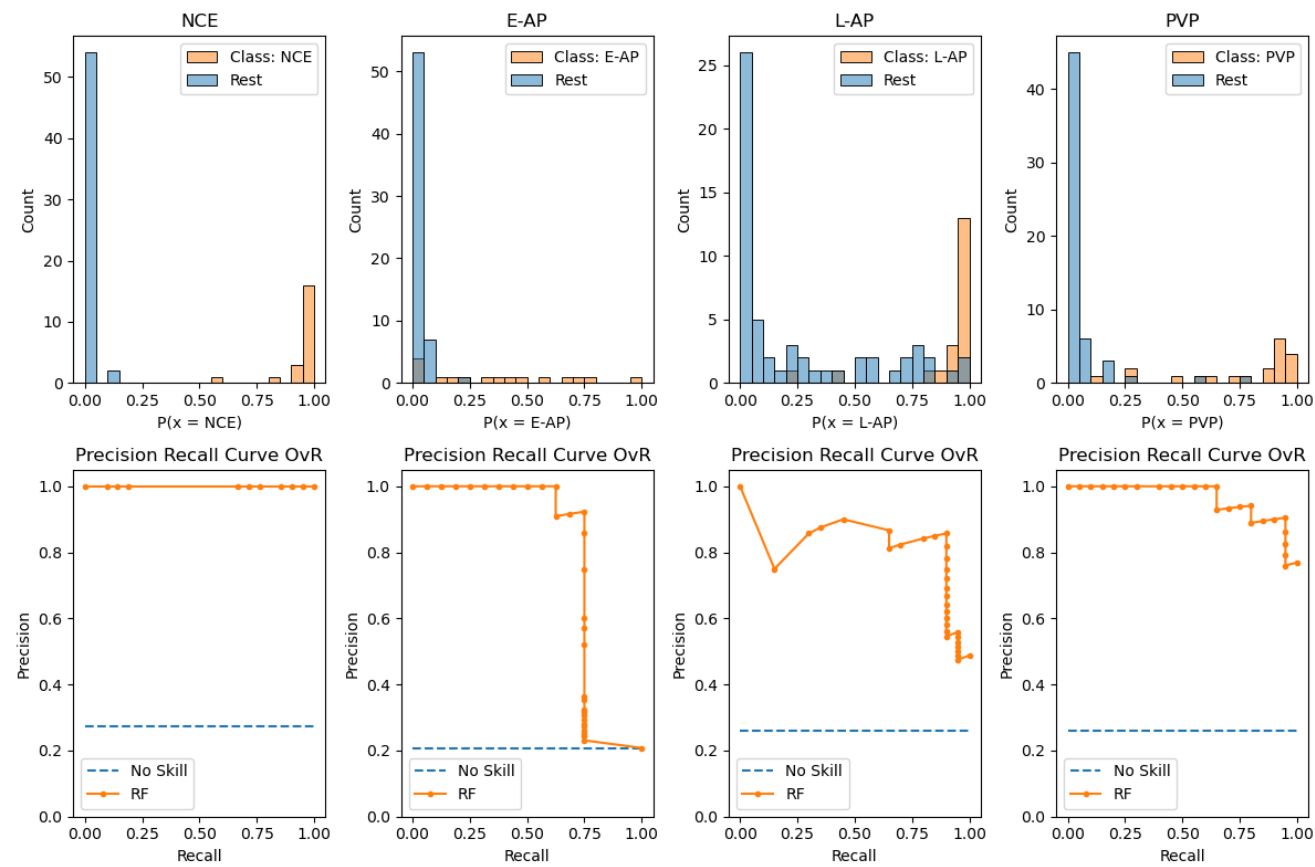

## RF OvO

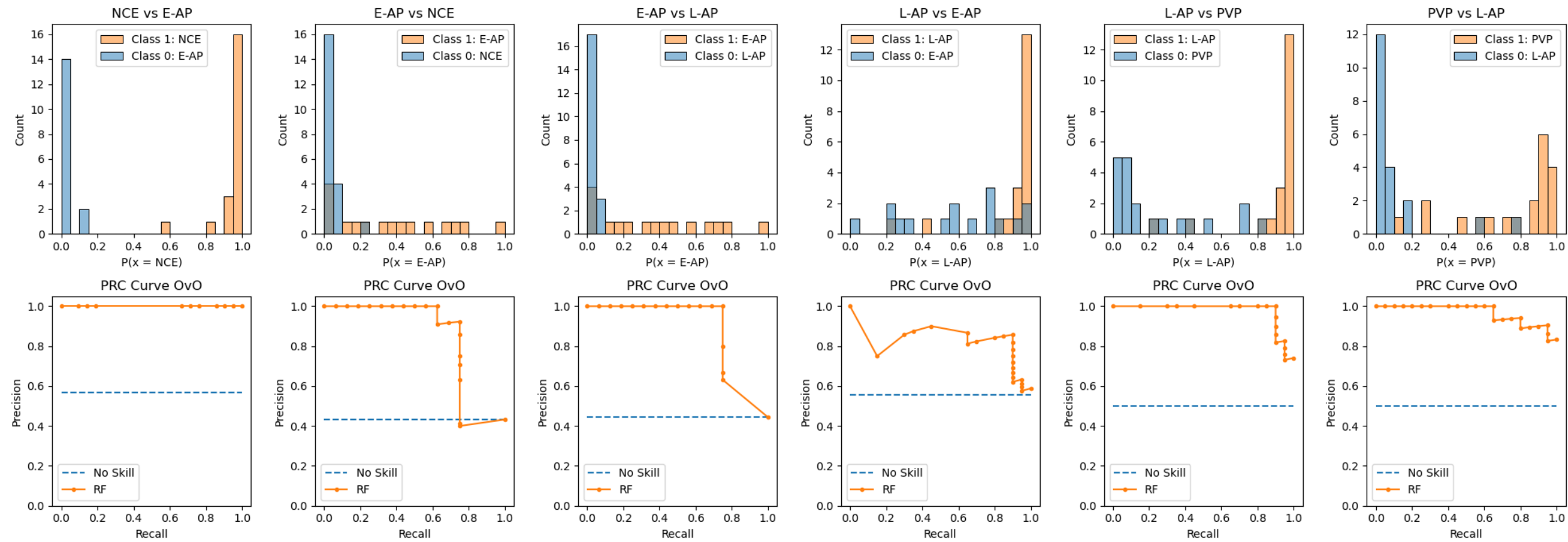

E) GBDT OvR

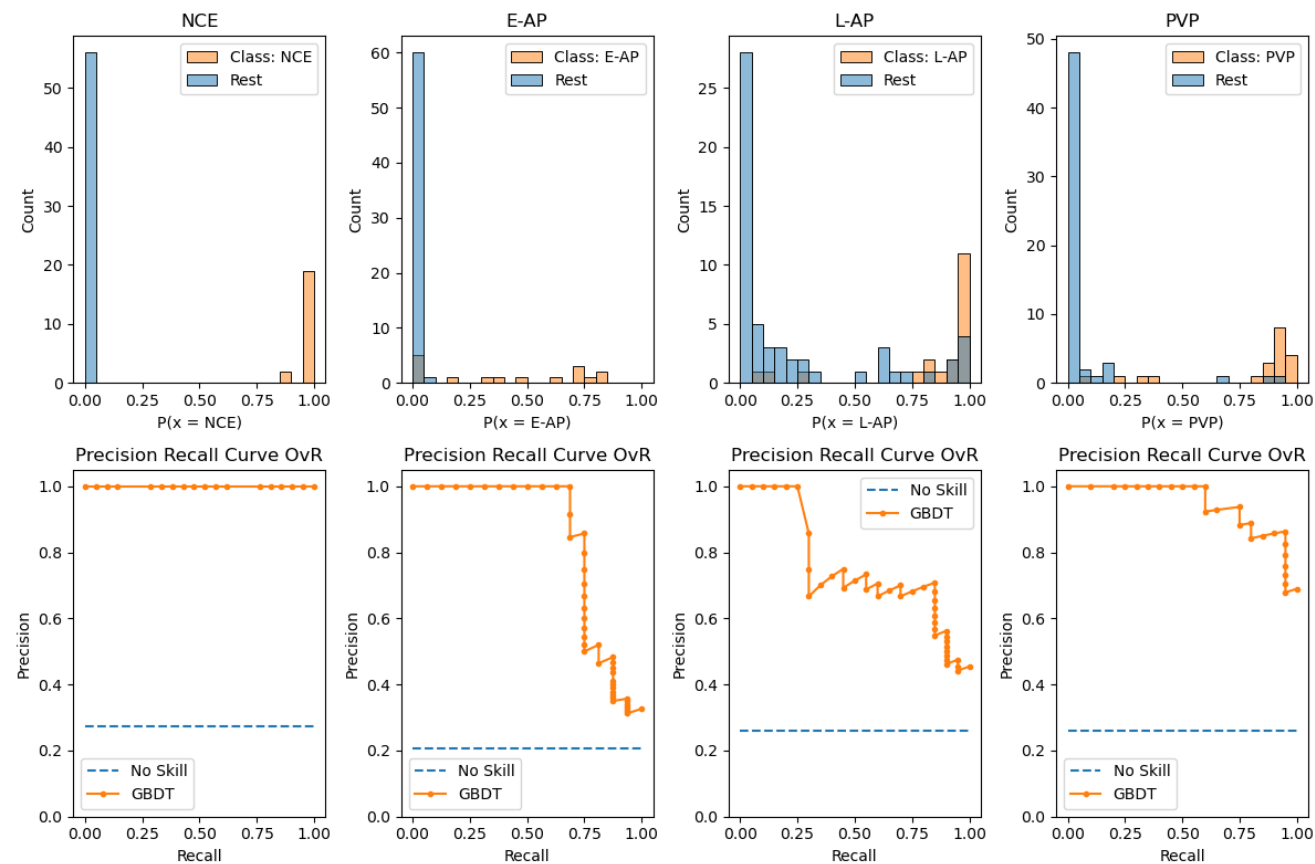

GBDT OvO

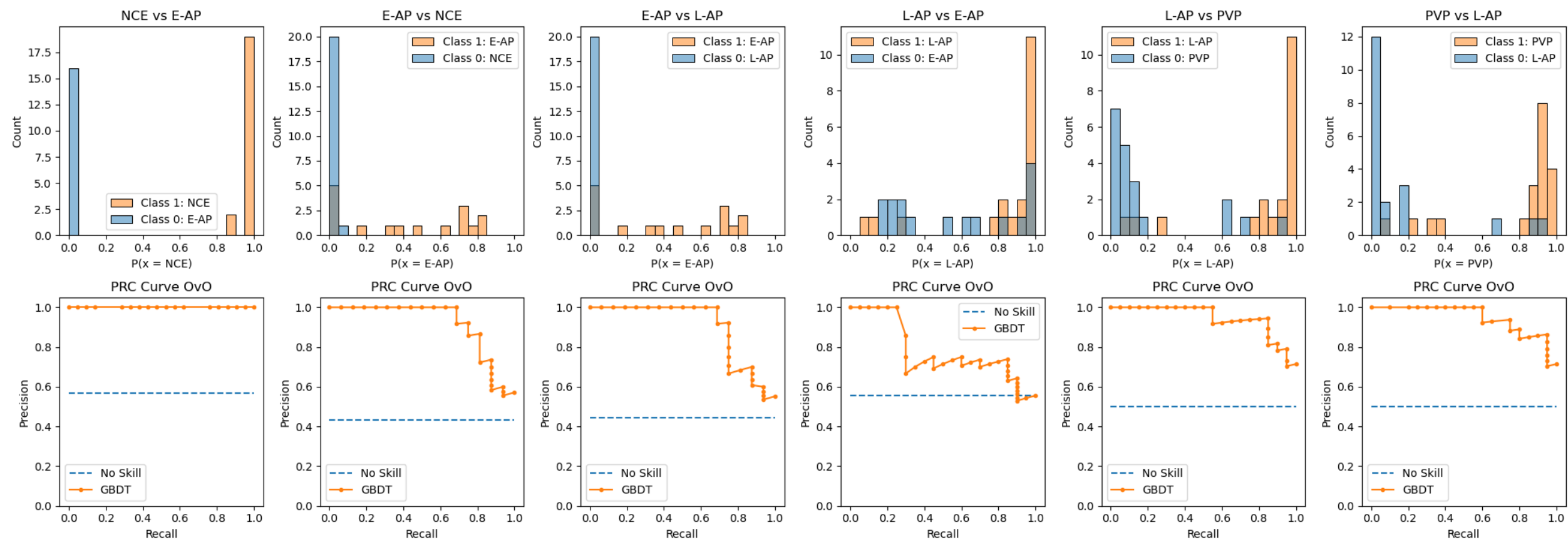

Supplement: S8 Fig — (A), (B), (C), (D) and (E) display the PRCs for the logistic regression (LR), support vector machine (SVM), decision tree (DT), random forest (RF), and gradient-boosted decision tree (GBDT) models, respectively. For each model, the graphs evaluated using a One vs. Rest (OvR) approach are shown on the top and a One vs. One (OvO) approach are shown on the bottom (note that only the OvO PRCs for consecutive phases are shown). See S2 Fig for more details on their interpretation. (PDF) [file pone.0294581.s008.pdf]
